# Inter-household transfers of material goods among Sama “sea nomads” of the Philippines: Reciprocity, helping, signaling, or something else?

**DOI:** 10.1371/journal.pone.0290270

**Published:** 2023-08-24

**Authors:** Julia R. Phelps, Kier Mitchel E. Pitogo, Angelica T. Emit, Kim Hill

**Affiliations:** 1 School of Human Evolution and Social Change, Institute of Human Origins, Arizona State University, Tempe, Arizona, United States of America; 2 Institute of Biological Sciences, University of the Philippines Los Baños, Los Baños, Laguna, Philippines; 3 Department of Marine Biology, Mindanao State University, General Santos, South Cotabato, Philippines; University of Zaragoza: Universidad de Zaragoza, SPAIN

## Abstract

The extent to which humans share with both kin and non-kin is a defining characteristic of our species. Evolutionary research suggests that pervasive reliance on inter-individual transfers of goods and services may have evolved to support a cooperative breeding adaptation in humans. However, while intensive *food* sharing between individuals and families has frequently been investigated in small-scale human societies, a comprehensive analysis of the daily transfers of *all* material goods has not been attempted. Likewise, while much previous research on cooperative transfers focused on terrestrial foraging populations, less attention is paid to other small-scale economic modalities traditionally inhabited by humans. Drawing on over three years’ worth of interviews and observational data from a community of primarily ethnic Sama people residing along the coast of Southern Mindanao Island in the Philippines, this paper examines the overall transfer patterns of material goods in a marine foraging economy. A quantitative description of resource acquisition is followed by an in-depth exploration of the characteristics of individual households and household dyads who gave and/or received more during the study period. Results indicate that a household’s age and income are consistently correlated with increased inflow and outflow of material goods. Results also suggest differential motivations underlie inter-household sharing of food, money, and other goods in the study community. Most importantly, we find that both daily and long-term reciprocity overwhelmingly drive sharing within household dyads in the study community, despite secondary effects of kinship, relative need, and relative household age between household dyads.

## Introduction

Humans are extraordinarily dependent on assistance as juveniles and assistance raising offspring when they become adults. A pattern of net production deficits in early life and net production excess, later in life, appears to be a human universal, from hunter-gatherers to modern industrial states (e.g. [1–3]), and is the consequence of the human pattern of learning-based resource acquisition. As a result, human societies are universally constructed around economies that facilitate production and transfer of goods and services between individuals and families. This cooperative pattern of resource transfers emerges because of asymmetries in labor availability, dependency load, productive efficiency, and in the need for specific resources and services at different times.

The daily transfer of goods and services in human societies is due to both the socio-reproductive system of help-based *cooperative breeding* [4–11] and the complimentary nature of learning-based human economies (e.g. [12]). Age differences in productivity and skills are universal in human societies, and the potential fitness impacts of age-structured transfers are a critical component of human life history (e.g. [13]). Human solutions to these adaptive challenges range from extensive food sharing [14] to behavioral complementarity and divisions of labor across sexes and individuals (e.g. [15]). The pattern of transfers extends to informal exchange of resources and labor in all human societies (e.g. [16, 17]), but also includes transfers of information and knowledge [18]. Finally, community-level cooperation frequently extends to common defense, modification of living space, organization of symbolic displays and ritual activities, and the production of public goods such as utilities and transportation systems in larger-scale societies [19, 20].

*Transfers* of goods and services between individuals and families in human societies is universal. But, the two functional realms of cooperation, *assistance* and *reciprocity*, have rarely been studied simultaneously. Both kin and non-kin assistance in humans is widespread in form, varied in source (kin, non-kin, ritual partners, different age and sex groups), extensive in scope, and deep in quantity of resources transferred over time (e.g. [5]). Systems of “informal trade,” consisting of reciprocal exchanges that are not negotiated or formalized with payment terms (see biological market theory–[15, 21, 22]), appear inevitable given the complexity of the human resource acquisition niche. Finally, the realities of individual comparative advantage, learning-based efficiency, and economies of scale make specialization and exchange optimal within family groups and frequently interacting units (e.g. [23]). In short, human socioeconomic patterns represent a unique socioeconomic niche not seen in other social organisms [24].

Recent research has produced a substantial body of quantitative research on food transfers, both within and between households, in hunter-gatherer and tribal societies over the past three decades (see [17, 25] for reviews). Importantly, however, food sharing may not be representative of other material transfers, because food has special properties, such as low storability and minimum required daily consumption, that incentivize regular transfers to reduce daily acquisition variance. Occasionally, researchers have also examined labor transfers such as garden labor exchange or childcare (e.g. [15, 26, 27]), information transfers [18], the exchange of different utilities such as meat for coalitional support (e.g. [28]), and the effects of sharing generosity on received social support [29]. However, documenting the transfer of all food and non-food material goods in small scale societies, as well as transfer of all services simultaneously in a single study population, has not been attempted.

In this study, we examine the age-sex specific patterns of resource acquisition and non-commercial inter-household transfers in a population of Sama marine foragers, in order to investigate the simultaneous flow of all material goods, both “in-to” and “out-of” all village households, on a day-by-day basis. Services will be examined in a later paper, because the full suite of “helping” behaviors that are provided in a small-scale society has never previously been documented and is extensive and complex in our study population. In subsequent papers, we will also examine whether goods and services are regularly exchanged among reciprocity partners.

A household (HH) is defined here as a group of individuals, usually an extended nuclear family, that sleep together in the same structure and eat most meals together. Individuals within HHs generally pool most of their daily economic production, hence we did not monitor intra-HH transfers in this study. Non-commercial transfers of material goods between HHs included not only food, but also tools, medicines/drugs, adornments, clothing, fuel, bait, other production supplies, and cash that was “shared” between village HHs without explicit agreement or expectation of any specific payment. Hence, recorded transfers did not constitute commercial transactions or formal loans, but are instead best described as *sharing*. The pattern we describe here presents a more complex theoretical challenge to understanding cooperative sharing, because many of the material goods transferred between Sama HHs are both storable and have significant increasing utility value to donor HHs as greater amounts are retained rather than transferred (e.g. cash transfers).

Our descriptive findings appear both unique (no other study that we are aware of reports daily transfers of *all* material goods between HH) and notable: Inter-HH transfer of material goods, on a daily basis, is spectacularly high, with mean daily HH inflows from sharing approaching the equivalent value of the mean daily HH income from all productive activities. In other words, HHs obtain almost as many resources from sharing as they do from their own resource production labor each day. Transfers include an impressive array of items beyond food resources.

In this paper, we develop two classes of analytical models. The first models examine which types of HHs in the village give more to other HHs each day, and which types of HHs receive more. One possibility is that a small subset of HHs are the most important donors, and a different subset are frequent recipients. If the high-donor HHs are wealthy and the high-recipient HHs are needy (in relation to one another), this might indicate that most transfers represent economic assistance. Alternatively, high levels of giving by wealthy HHs might also represent costly signaling, whereby donor HHs gain status or prestige by displaying publicly that they are better off economically than other HHs. But other possibilities can also be considered. For example, if high recipients are mainly wealthy and the donor HHs are mainly poor, transfers might represent exploitation due to power differentials (e.g. political, social, physical threats, etc.). Alternatively, we might find that the same HHs in the village are both the high donors and the high recipients. Such a pattern might suggest that transfers in the village represent some type of balanced reciprocity or exchange. If so, we will need to examine what conditions stabilize and favor reciprocity (variance, specialization?) when transfers are not regulated by formal or enforceable contracts. Lastly, we might find that a few HHs receive resources from a large number of village residents and then redistribute them back out to different village residents (perhaps more needy HHs). Such HHs could be acting in a redistributive role. Indeed, some informants in our study indicated that political or religious leaders might sometimes be expected to play such a role.

Our second class of statistical models aims to determine which kinds of HHs are likely to be the most important donors to, or recipients from, specific other HHs. In particular, we examine how kinship, dyadic reciprocity, differences in need, HH proximity, and age differences between HHs might associate with transfer rates between specific pairs of HHs. The models examine dyadic relationships between families that might influence resource transfer patterns.

This study produced several important findings. First, reciprocity is by far the most important determinant of resource transfers, and reciprocity between households is detected on both short time scales (daily) and long time scales (over the 3-year study period). Second, kinship is an important predictor of resource transfers, and needy kin do receive more help. However, transfers are especially high to kin that reciprocate regularly, suggesting that inclusive fitness gains may not be the main motive of transfers to kin, especially since the study population contains a large collection of distant kin. Third, some resources like food and money are shared very differently than other types of resources, and different age classes typically transfer different types of resources. These differential patterns of resource transfers need to be studied further in conjunction with our future analyses of service transfers between households. It seems likely that cooperative partners regularly transfer different types of resources and services (different *currencies*) back and forth in order to take advantage of specific needs and abilities.

## Materials and methods

### Study population

The study village, called *Linao of the Mangroves* (hereafter, *Linao*) was established progressively in the 1980s on the coast of Southern Mindanao Island (Sarangani Province) by a growing population of predominantly ethnic Sama tribal people. Some descendent members of the community also have mixed Tausug heritage, and many families in the community adhere at least partially to Muslim religious traditions. The Sama traditionally lived on large outrigger canoes equipped with sails and living quarters, and they moved frequently over large areas of SE Asian archipelagos, fishing and trading for carbohydrate plant foods [30]. However, in the past century most Sama populations have settled to live on bamboo or wooden palm thatched houses built above shallow ocean reef flats near the coastal shoreline (see S1 Text for more cultural background).

From June 2015 to November 2018, the Linao study community consisted of 25–40 huts connected by a crude wooden catwalk system built on tree posts about 2 meters above the ocean at high tide. During our study period, 58 invertebrate species were collected, but 84% of the intertidal food-energy/trade-value came from only 5 species. Most commonly harvested were sea slug eggs, medium-sized bivalves, sea urchin eggs, and small gastropods. Beyond the reef, but usually within sight of the community, fishing activities employed both small surface float-net technology (3–5 men in a boat within a couple kilometers of the settlement) and hook-and-line methods (1–3 men per boat, usually within a kilometer or less), with some spearfishing as well. Economically most important fish species included mackerels, sardines, carangids, snappers, and a variety of reef fishes (in that order by weight contribution).

Our sample covered 3 ½ years of monitoring and ~36 *households* (HHs) in most sample months. The entire village was carefully censused approximately once a month. Mean HH size was 4.85 individuals (range 1–12 individuals), with some HHs splitting or fusing during the study and some HHs leaving for extended periods before rejoining the community. HHs were usually composed of extended kin groups (one or two nuclear families plus an aunt, uncle, grandparent, etc.), but a few contained unrelated individuals (all males). The mean village size during the sample period was 113 persons, but ranged from 87–149 persons on monthly census days.

Dyadic (pairwise) relatedness *r* between individuals in a dyad was measured as the probability that a randomly-selected allele would be identical by descent in both individuals in the dyad. Analyses of dyadic relationships between all individuals who lived in Linao during the study period shows that only 9% of all possible dyads of individuals in the village population were close kin (genetic coefficient of relatedness *r* > = 0.125), but that 46% of all possible HH dyads had at least one resident in each HH related as first cousins or closer. In this study, the dyadic coefficient of biological relatedness between HHs (*r*_*HH*_) is calculated as the *r* value between the two residents in the HH pair with the closest genetic relationship. By this criterion, about half the HHs in the village are either distantly related or unrelated to each other.

The Linao community is partially integrated into the local market economy. All resources are initially collected from the environment, but some of the harvested resources are sold or traded outside the village on a daily basis, and a few residents work for wages or engage in small scale commerce (see below). None of the HHs in Linao plant any crops, and farming is nearly absent in the immediate area because local rainfall is not sufficient for most crops. (Linao is located in a coastal mountain rain shadow.) Linao has no running water, no electricity, and no structures for human waste disposal. Approximately 50–70% of children attended school during the study period, but virtually all village children had dropped out of school by age 10–14. Ethnographic studies report that the Sama were traditionally uxorilocal [31], but residence among newly-married adults in Linao was bilocal, with 3 new husbands moving in and 4 new wives moving out of their HH compound during our study period.

Anthropometric data indicate moderate levels of nutritional stress in the study community. This interpretation agrees with income data (see below) and direct measurements of low daily food intake. A sample of 1985 consumer days during our study period showed mean 24-hour food intake valued at just $0.88 per capita per day. The diet consisted mainly of rice (47% of total value), fish and invertebrates (25%), breads and noodles (9%), fruits and vegetables (8%), coffee (5%), and eggs (2%). Only the marine foods in this diet were locally harvested, with the remaining food types traded for marine foods or purchased. Children of both sexes show age-specific weights around the 10^th^ percentile for American children, with mean adult male weight of 54.7 kg (*n* = 81) and mean adult female weight of 47.1 kg (*n* = 75) in the study population. Further ethnographic and demographic details of the study population, including mortality and fertility patterns, are provided in the S1 Text.

### Data collection and analysis

The data used in this study was collected in Linao village between June 2015 and November 2018 as part of an ongoing study. A total of 36 HHs are classified as part of the village study sample, and only individuals who were identified as members of a HH by a head of that HH were included in this study sample. A total of 493 separate *HH days* (i.e., a full day’s worth of data for a particular interviewed HH) were recorded during the sample period, although more than one HH was interviewed on the same day in some cases. Data collected during the sample period include measurements of all daily resource production, resource transfers, and food consumed by members of a HH on a day that they were observed, as well as demographic, genealogical, and village/HH composition data for all Linao residents. An additional interview was conducted after the conclusion of the main data collection period to assess community views surrounding which HHs give and receive more resources.

HH composition and daily resource production, transfers, and consumed food were ascertained via a 24-hour recall interview protocol. Interview participants were a head of HH (usually the female head of HH) for the HH being interviewed. Interviewers first asked the participant to list all members of their HH and all resources produced and consumed by these HH members in the past 24 hours. Participants were then asked to list all resources shared to members of their HH from individuals not residing in their HH, followed by all resources shared by a member of their HH to an individual not residing in their HH. A separate 24-hour recall interview was used to elicit all fish resources captured by Linao men during each fieldwork day (*n* = 5080 man days). In a subset of these interviews (*n* = 4788 man days), time spent fishing and performing fishing-related activities (e.g. maintenance of fishing equipment) was also recorded. Additionally, researchers directly observed 1622 intertidal foraging bouts during the sample period, in which they used a stopwatch and scale to record all time spent and marine resources acquired during the bouts. Detailed demographic and genealogical data was acquired via interviews with all adult residents early on during the sample period. When needed, subsequent interviews were used to validate conflicting information about individual relatives. All HH composition and genealogical/demographic data was verified and updated regularly via collection of monthly village-wide census data. Lastly, a separate interview protocol was administered to *n* = 36 adult participants (24 adult females and 12 adult males) at the conclusion of the main sampling period. This interview elicited informant perceptions of the characteristics of HHs who gave and received more resources, and it also asked residents to freely nominate households that gave/received the most. All interviews were conducted in the Cebuano (Binisaya) language by Filipino student research assistants from Mindanao State University, and responses were recorded directly in English and then coded into a digital database.

Quantification of all resource measurements was performed by first calculating the value of the resource in Filipino pesos (according to local market rates, as elicited from participants), and then converting this value into U.S. dollars (US$ in text; USD in plots) using appropriate time-specific conversion rates. This allowed for different forms of resource production to be compared in terms of individual or HH income. In the case of fishing and intertidal gleaning harvests, weight in grams or kilograms and time spent was either measured or elicited from participants, depending on the method of data collection. The coefficient of relatedness (*r*) between pairs of individuals in the study sample was calculated using the method first proposed by Sewall Wright [32], which approximates the average number of alleles that are identical by descent between the two individuals. HH relatedness between HH dyads (*r*_*HH*_) was then calculated as the maximum pairwise relatedness between all pairs of individuals across the two HHs (i.e., where one individual was in one HH and the other individual was in the other HH).

Except where explicitly noted otherwise, individuals in the population are considered to be *adults* if they are of age 18 years or older, while children are defined as those individuals below 18 years of age. Note that the choice of 18 years of age to delineate these two age categories is somewhat arbitrary and reflects a common convention within recent anthropological literature. However, this cut-off point may also be justified by noting that 18 years of age is at or around the mean age of first reproduction for females in many foraging societies (e.g. [1, 9]) and is the mean age at which Linao women first marry (see S1 Text), suggesting that 18 years of age is a reasonable threshold for determining adulthood.

All data deidentification, cleaning, tabulation, and analyses were performed in the R statistical software package, version 4.0.3. For most analyses, data from all interview and observational sources were combined into one full data sample across the 493 observed HH-days. (An exception to this is the descriptive analyses of fishing and foraging bouts, which use all complete observations made during the sample period.) Descriptive analyses were calculated using the entire 36-HH study sample, including all observations where a HH was present in the community. Except where explicitly noted otherwise, only resource transfers shared between members of the 36-HH study population were included in resource calculations (that is, recorded resource transfers to/from individuals outside of the population are not included). A few individual-level daily observations with missing data (not recorded) were omitted from the analysis, as was one outlier transfer observation in which an unusually large quantity of resources was given as part of a wedding gift. For HH-level analyses, measurements such as resource production and resource transfers were aggregated within HHs by summing the total amount produced, received, or given by members of the HH during a sampled (interview) day. Other metrics (e.g. mean of daily values) were reported in some cases, and are described in the context of their usage. Similarly, population-level and individual-level descriptive analyses were aggregated either at the daily level or across the sample period, and metrics across these two time scales (typically either totals or means) are also indicated in the context of their usage. Where appropriate, locally-estimated scatterplot smoothing (hereafter LOESS) curves were calculated and included in descriptive plots to facilitate interpretation of underlying patterns. Following convention, confidence intervals around LOESS curves were approximated as 95% *t*-distribution intervals. Reported correlations between variables were calculated using Pearson’s formula, and are denoted as “Pearson’s *r*” to avoid confusion with the coefficient of relatedness (*r*). Tests of binomial proportions were performed using 95% Wilson Score intervals with a Yates continuity correction.

All statistical models reported in the main text were constructed as generalized linear regression models on a 32-HH subset of the data for which all HHs were interviewed at least three times and for which all resource production variables (both HH production and HH fishing income) were completely measured across all days. This model subset included *n* = 455 of the total 493 interview observations recorded during the study period. In all models, all explanatory variables were standardized to permit cross-coefficient comparisons of effect sizes. Outcome variables were not standardized, so that effects could be interpreted as changes in the outcome variable (measured in US$) with respect to standardized unit changes in the explanatory variable. Notably, while some HHs moved out of Linao village for weeks or even months at a time (and hence were sampled less frequently), all 32 HHs who met the conditions for inclusion in the models (see above) were included in all models. Nonetheless, to ensure that statistical patterns were not being influenced by outlier behaviors in less-sampled HHs, we repeated all analyses twice: First, using only those HHs who were sampled at least 10 times, and then using only those HHs who were sampled at least 20 times (not shown, but see additional discussion in S1 Text). Results of these robusticity checks were largely similar across all models, with differences in significance resulting mainly from lower sample sizes, which indicates that patterns detected in the full models were representative of all HHs in the model sample.

Significance of model explanatory variables was assessed at an α = 0.05 significance level, using conventional *t*-tests. For some variables, additional marginal effects plots were examined to facilitate understanding of complex trends. In these plots, all other independent variables were fixed at their standardized mean values (approximately zero), and 95% *t*-distribution confidence intervals were reported around predicted marginal effects to give a measure of estimate uncertainty. Overall model fits were assessed using the Wherry/McNemar adjusted *R*^*2*^ (hereafter adjusted *R*^*2*^) statistic provided by default in the R implementation of ordinary linear models, which approximates the variance in the outcome variable that is explained by the regressors in the model. To test for HH-level effects, generalized linear mixed-effects regression and generalized linear fixed-effects regression models were also performed. However, these models proved to be ineffective due to the correlations between HH effect and other important HH-level variables in the model (e.g. age, income, etc.), and are thus only included in the S1 Text.

### Ethics

Permission to access the site and collect data was granted by Linao community leaders in consultation with village residents. All informants provided written informed consent in the local language, and all protocols were approved by the Arizona State University Institutional Review Board (STUDY00001593). Community leaders and representatives repeatedly indicated verbal enthusiasm for the project, and they voted unanimously to renew research collaboration with our team in the summer of 2022. Researchers provided general village access donations (used for community public works) several times per year, paid an hourly interview fee, and paid most resident emergency medical expenses during the study period.

### Inclusivity in global research

Additional information regarding the ethnical, cultural, and scientific considerations specific to inclusivity in global research is included in the S1 Checklist.

## Results

### Food production and daily income

Most members of Linao HHs acquire resources from the environment each day, and some produce additional cash income. Men fish, and to a lesser extent engage in collecting, with about half also engaging in occasional paid labor at least once during the study and about 11% engaging in salaried labor. Women collect terrestrial resources and marine invertebrates, buy-and-sell local resources at a small profit (*resale*), and a few women obtained paid labor at a fish cannery during part of the study period. Children’s resource acquisition was mainly limited to collecting marine invertebrates. Fishing was monitored for all men in the village on each fieldwork day, using 24-hour recall interviews. Men mainly fished in small groups of 3 to 6 men in one hand-made outrigger boat propelled by sail or small motor. Cultural norms of fish-harvest division reduce income variance somewhat between HHs of the village; however, daily variation between fishing groups is still substantial. Two types of division rules apply: 1) *Pahayag*/*Panlamba*, for nearshore daytime (net or line) fishing with a small boat and no motor, stipulates that every fisherman in the boat team gets an equal share (usually 3–5 men); 2) *Palaran*, for far offshore nighttime net fishing in motorized boats, stipulates that 10–20% of harvest goes directly for the gasoline and boat maintenance, with 30% of the remaining income allocated to the net and boat owners, and 70% of the remaining harvest divided equally among all fishermen–with juvenile teens receiving ~90% of the adult share.

**Fig 1:**
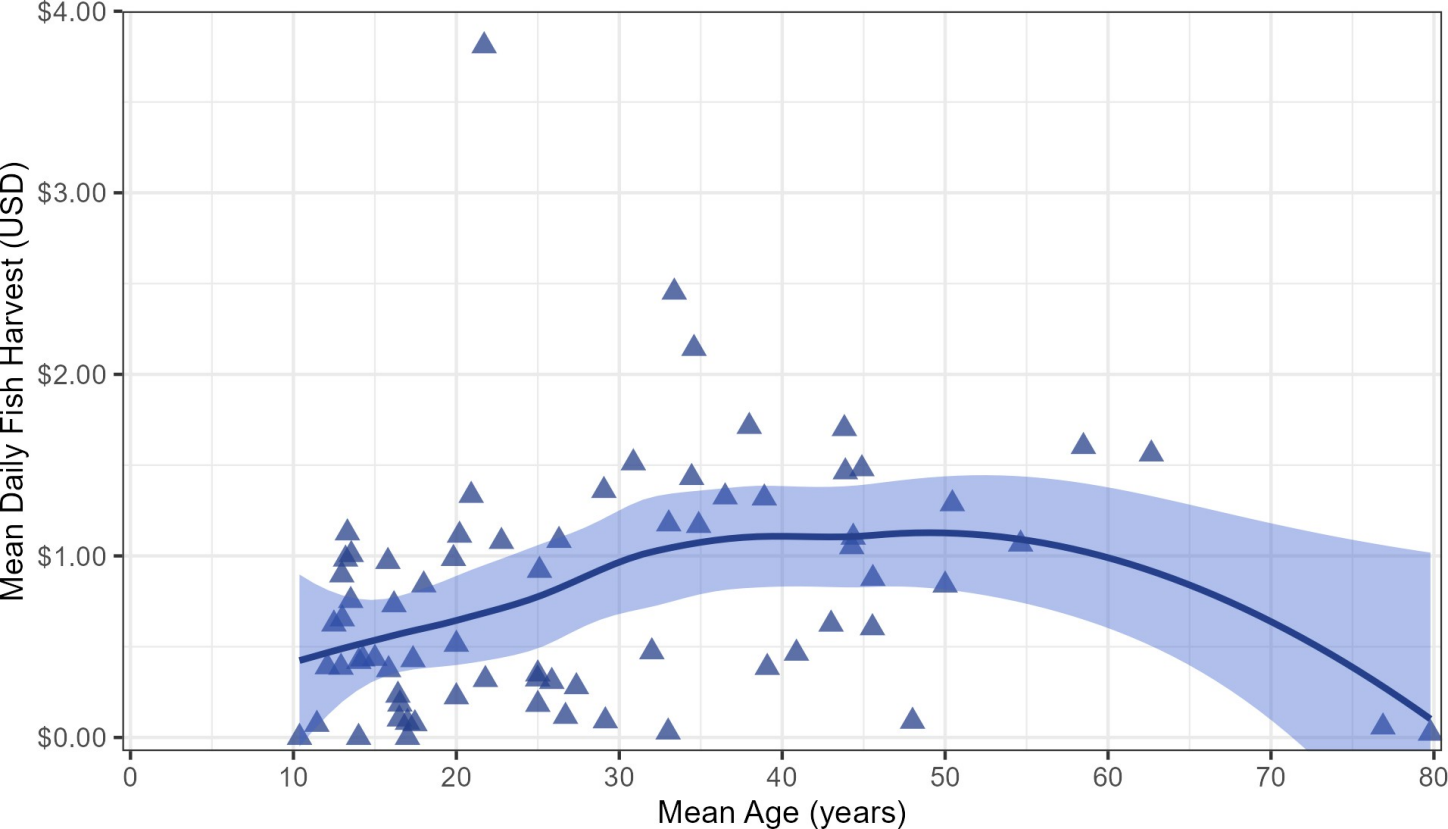
Mean daily fish harvest by age. Mean daily fish harvest by mean age for all males in the study sample, including observations of 52 Linao adult males and 21 juvenile males who are at least 10 mean years of age. (To enable distinct counts of adult and juvenile males, note that adult males are defined here as individuals whose max observed age during the study period was ≥ 18 years old, and juvenile males are defined here at those who were < 18 years old throughout the entirety of the study period.) Sampled males come from 35 of the 36 HHs; one adult male residing in the remaining HH was omitted from the plot because their fishing returns were only sampled twice. The points show mean rate by mean age for individual males, whereas the fitted line is a LOESS smooth of the data. The blue band around the fitted line represents a 95% confidence interval.

Interviews show that approximately 3086 kg of fish were captured by Linao men living in the 36 sample HHs on sample days from June 2016 through November 2018. Fishing harvest value per day increased for males during adolescence years, reaching a plateau by the mid-30s that declined steadily to almost zero production by age 75 (Fig 1). The mean market value of the fish harvest for men 18 years and older was $1.01 (0.61 kg raw whole fish) per day sampled (*n* = 53 men, 5080 man days). Despite group fishing and division rules, fishing harvest income (after division) still showed moderately high daily variance, with 60% of all man days showing $0 harvest (maximum harvest on a single man day was $51.52). A subset of interviews with recorded time in fishing activities (*n* = 4788 man days) showed that men went fishing on 43.6% of all days and spent a mean of 4.81 hrs fishing on those days (mean = 2.1 hrs per sample day when including non-fishing days). Men also spent a mean of 0.68 hrs per day engaged in net repair, preparing bait, and boat repair. Hence, *gross* mean hourly return rate is $0.40 while fishing, or $0.30 (0.19 kg) per hour including both fishing and preparation time. Mean *net* hourly gain is lower still, however, due to costs of equipment, supplies and fuel. Not surprisingly, Linao HHs are very poor, even by Philippine standards (see below).

**Fig 2:**
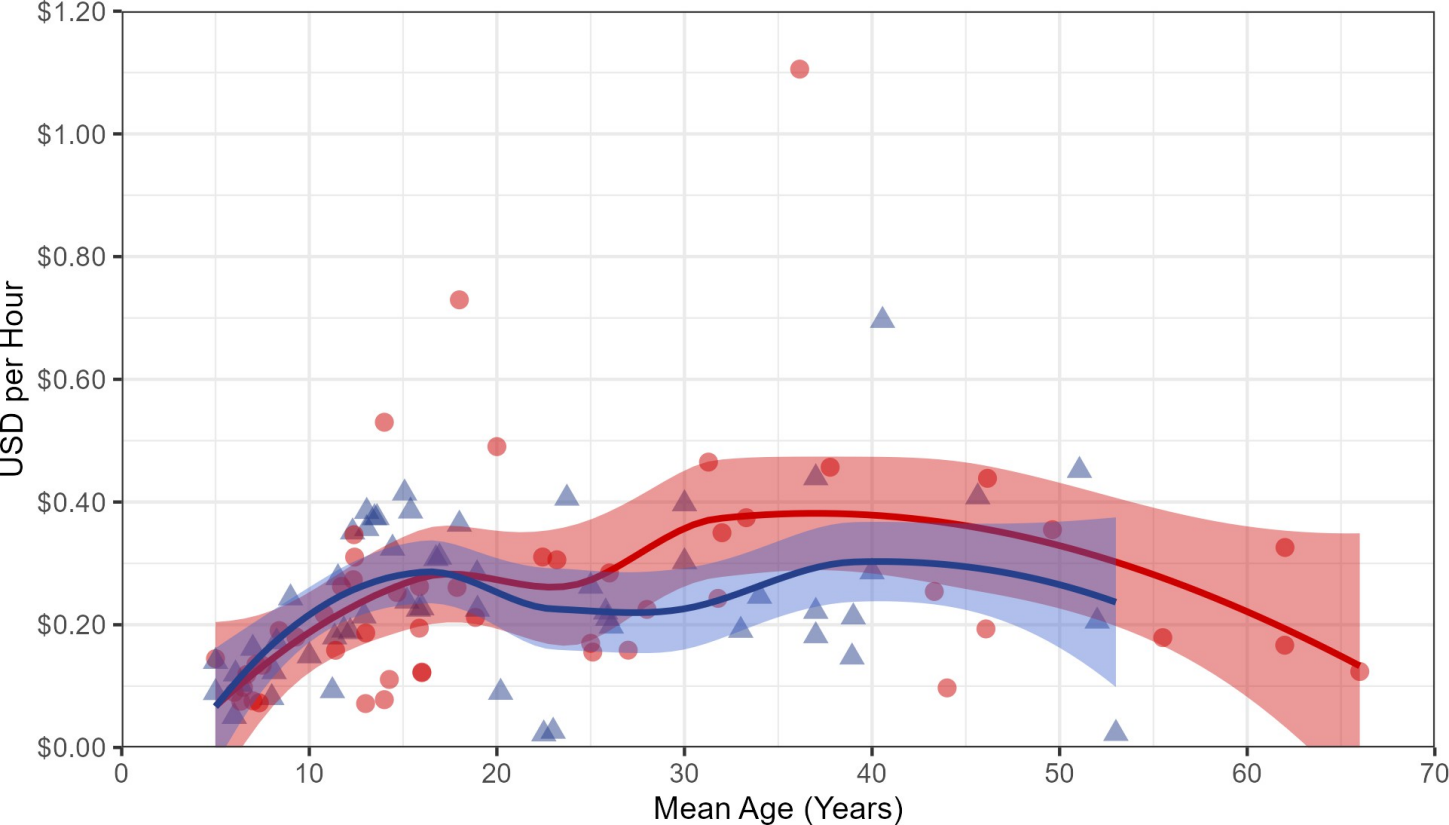
Mean return rate by age. Mean individual return rate (in US dollars per hour) by mean age, calculated from intertidal collection of marine invertebrates by 53 females (red circles) and 56 males (blue triangles). Solid lines show a LOESS smooth of the trend for each sex, and bands around these lines indicate 95% confidence intervals around the LOESS smooths.

Intertidal marine foraging, or *gleaning* (called *panginhas* locally), was also a common economic activity on the 7 days around the spring tides that occur twice each month (Fig B in S1 Text) and included children as well as adults. Resources collected consist of shellfish, crabs, small fish, seaweeds, and other marine invertebrates. Most of the harvest was consumed in-household, but a fraction was also sold in order to purchase rice. Between June 2015 and November 2018, we sampled (with stopwatch and weigh scale) 1622 foraging bouts (2814.8 person hrs) on the reef flat surrounding Linao, and recorded harvest data for 109 foragers of both sexes from 5 to 66 years old. All returns were converted into US$ value at local market prices (elicited from informants). Harvest data shows no sex difference in hourly harvest efficiency, with mean returns increasing steeply during the childhood years (until age 12) and then modestly during early adulthood, peaking at age 30–40 with an old-age decline beginning around age 45 (Fig 2). The mean adult female harvest rate was valued at $0.36/hr, and for adult males it was $0.30/hr according to local market rates. This was estimated to be approximately 300 Kcal per hour, based on published caloric values for similar marine species (Fig C in S1 Text).

Total HH income from all sources was elicited via 24-hour recall, for a sample of 493 HH production days (2232 person days) from June 2015 through November 2018 (Table 1). Female heads of HH were asked: “*List any food and non-food items of value (resources) that your HH produced or harvested yesterday (fish*, *game*, *wild plants*, *money from sale or labor*, *etc*.*) and list any associated costs of production (expenses)*.” All resources acquired were assigned local market value (converted into US$) for direct comparison across six categories of production. Mean daily HH gross income was $6.97 per day. Income is derived mainly from fishing (37%), resale (24%), and collecting (19%—firewood, water, plant foods, recyclable plastic). *Resale*, mainly an activity of adult women, consists of the local purchase of small quantities of harvested wild resources (mainly fish, firewood, and marine invertebrates) from neighbors, followed by travel to a selling location and subsequent price markup for small profit. A few HHs temporarily maintained small “stores,” where they purchased a dozen types of goods (e.g. fruit, snack foods, cleaning supplies) at a nearby town and then resold within the community at a small markup. Other productive activities of lesser importance included paid *labor* (temporary salaried work, providing services like sewing, boat repair, transporting passengers, and government benefits), followed by low-tide reef gleaning of marine invertebrates, and finally, *income* from renting out boats, nets, motors, and large tools.

**Table 1:** Total HH resource production from 493 sample days.

Type	Women Amount	Men Amount	Children Amount	All Individuals
**fishing**	$5.21	$1083.09	$182.02	$1270.32
**resell**	$731.21	$92.40	$1.19	$824.80
**collecting**	$142.93	$457.77	$52.43	$653.12
**labor**	$145.25	$322.54	$13.92	$481.70
**gleaning**	$63.81	$66.79	$56.43	$187.02
**income**	$17.07	$1.60	$0.00	$18.66
**Total**	**$1105.48**	**$2024.19**	**$305.99**	**$3435.62**

**Fig 3:**
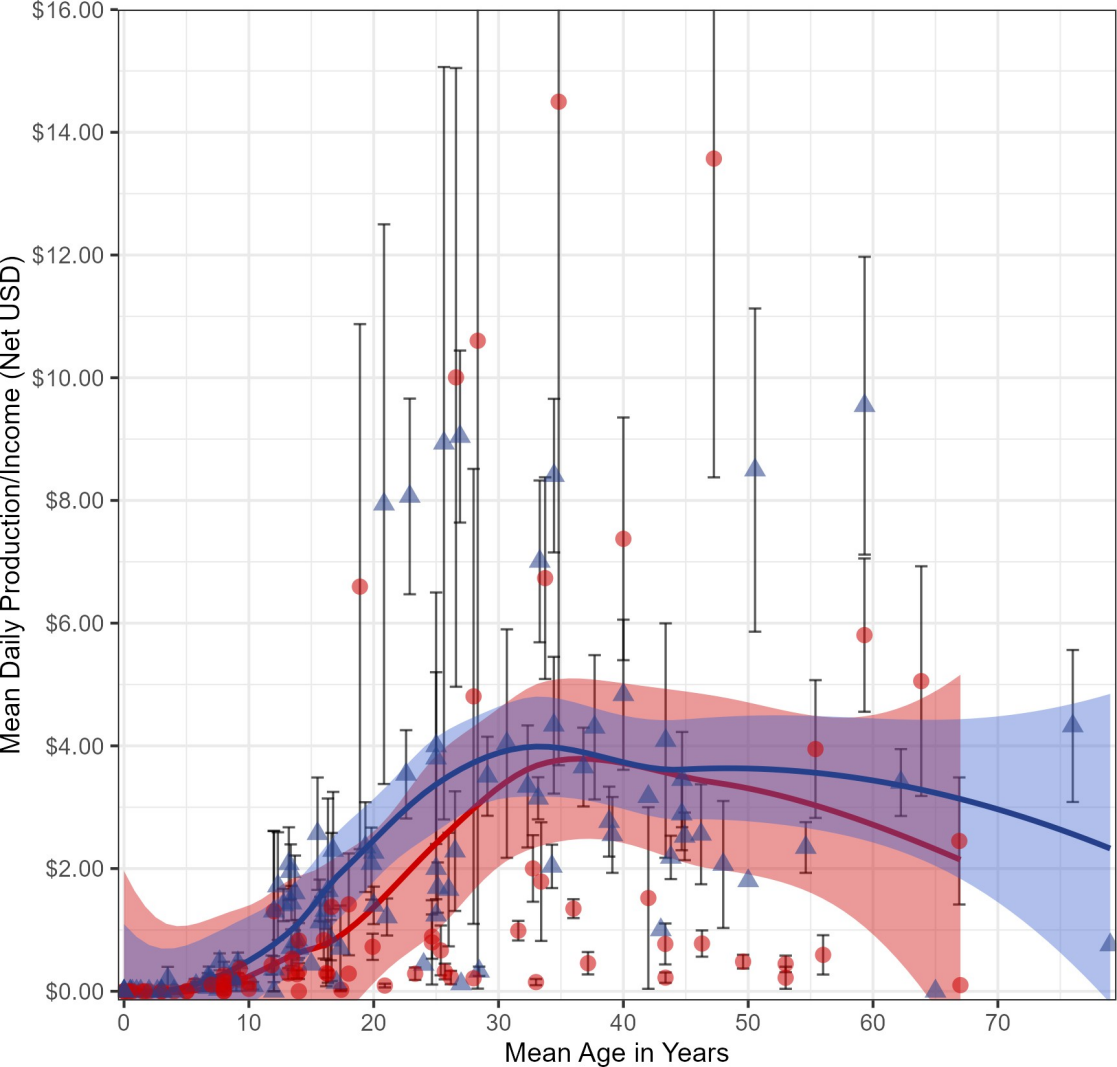
Mean production/income by age. Mean total daily production/income from all sources, by age, for 79 females (red circles) and 101 males (blue triangles). Error bars represent one standard error about the mean for individuals with > 1 observation. Fitted lines corresponding to each sex are LOESS smooths, and bands around these lines indicate 95% confidence intervals. One daily observation of a transgender female resident, equaling $4.08, has been omitted.

The age structure for total daily income/production for both sexes is similar (Fig 3) and typical of the age structure of production found in most small-scale societies (e.g. [1, 3]). Children produce almost nothing. Income increases from early teens up to mid/late 30s, and then begins to decline slowly, with a steeper decline in the 60s. Men and women’s mean daily adult incomes level off at about the same value in our village sample, but interestingly, the top 3 individual earners were all women involved in resale activities.

### Household income variance and inequality

**Fig 4:**
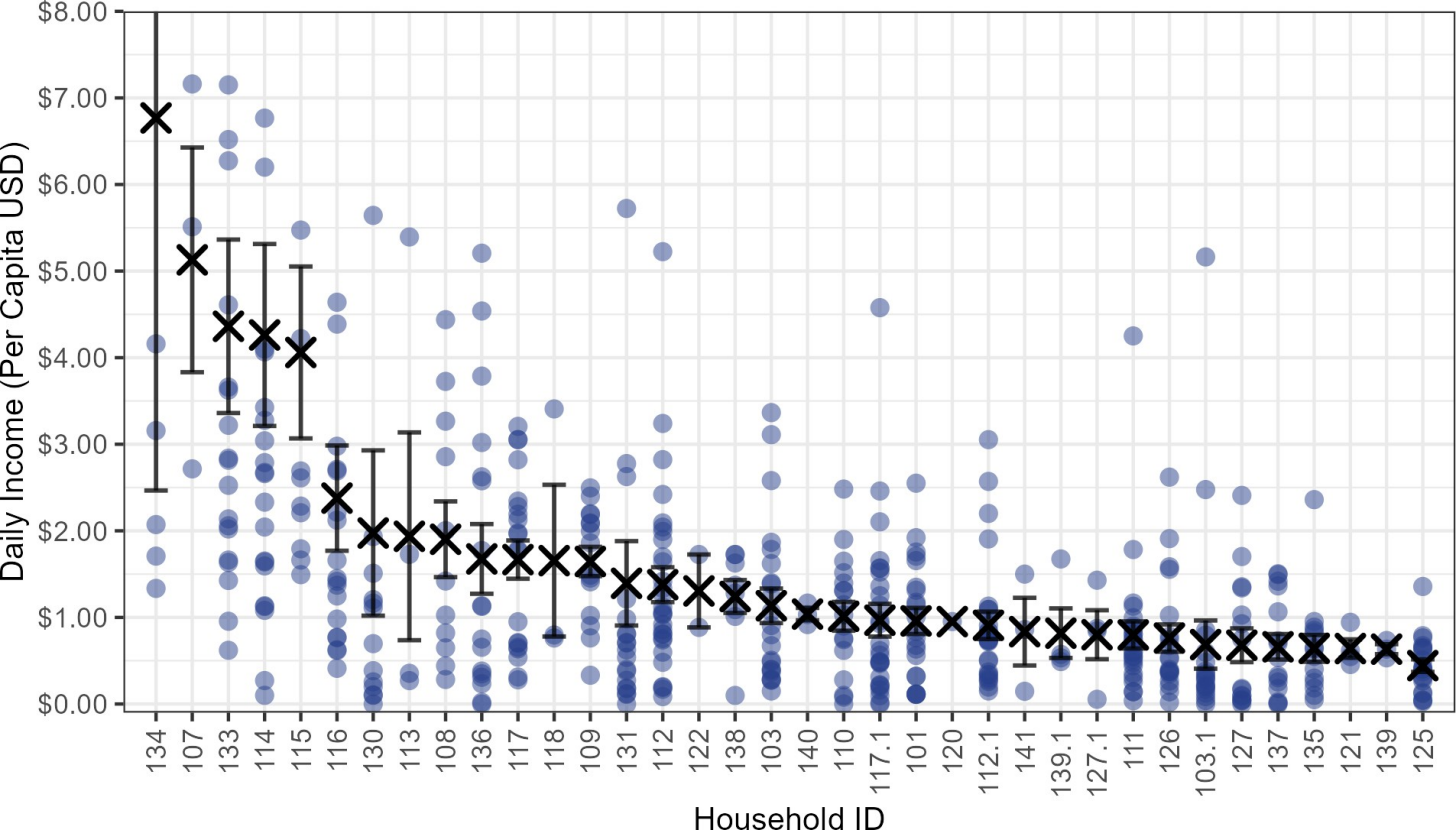
Mean HH daily per capita income. Mean daily per capita income from all sources for 36 HHs in Linao, presented in rank order. Xs are household means, while dots are daily household observations. Error bars represent +/- one standard error about the mean for all HHs with > 1 daily observation. A few large single-day outliers have been excluded in y-axis, but are included in calculations.

HH data illustrate considerable daily income variance, as well as inter-HH inequality. The highest recorded single-day HH incomes were greater than $70 (on 4/493 sample days), and the lowest single-day HH incomes were $0 (on 15/493 sample days). Mean daily HH income ranged from about $25 per day to around $1 per day (Fig D in S1 Text). When family size is taken into account, mean daily per capita HH income was $1.67 per capita/day, and ranged from about $7 per capita/day to around $0.50 per capita/day (Fig 4). The mean per capita HH income is therefore only about 1/5 of the Philippine National mean per capita daily income (2017) of $7.92 per person per day [33].

Daily variation in HH income showed a mean coefficient of variation for the 36 HHs of 0.88. Importantly, 28% of the HH days sampled showed a per capita daily income of less than ½ of the value of mean daily per capita food expenditure in Linao. No HHs monitored had any significant cash savings to buffer this income variability, and many commonly harvested foods do not store well. Hence, daily food sharing was probably critical to nutritional well-being for most HHs.

The degree of income inequality in the Linao settlement can be quantified by calculating the GINI coefficient for mean HH income. The estimated GINI value of 37.7 is somewhat lower than for Philippines as a nation (2015 GINI = 44.4 [34]). In Linao, the daily income of the top 10% of HHs was 10.8 times higher than the daily income of the bottom 10%. Technological *means of production* were also unequally distributed, with only 61% of HHs owning a boat, 43% owning a motor, and only 30% owning a net. In short, Linao HHs experience both asynchronous need, due to daily income variance, and moderate long-term inter-HH income inequality separating “rich” from “poor” HHs.

**Fig 5:**
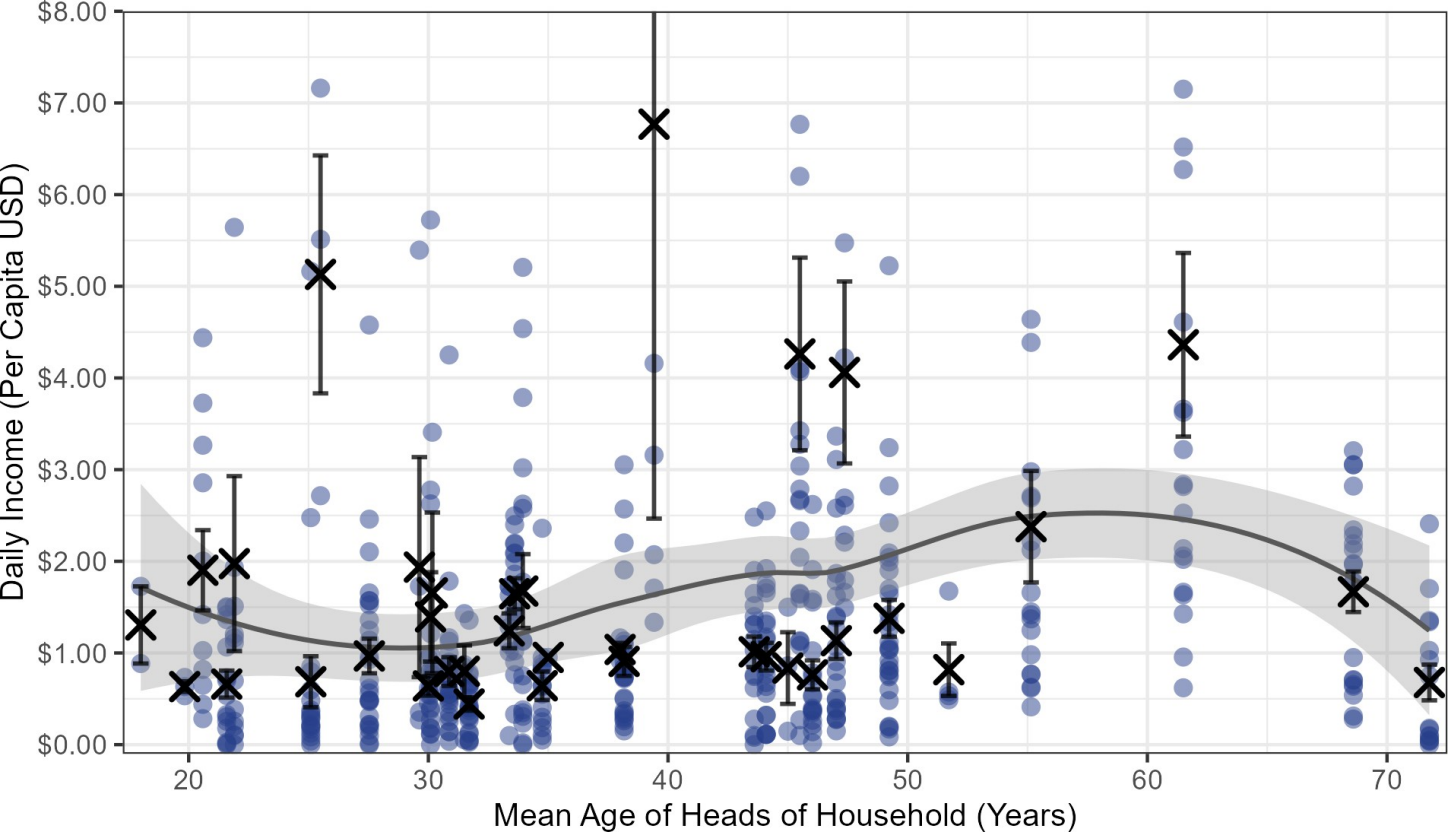
Mean HH daily per capita income by mean age of heads of HH. HH mean daily per capita daily income as a function of mean age of heads of HH for 36 HHs. Xs are household means, while dots are daily HH observations. Error bars represent +/- one standard error about the mean for all HHs with > 1 daily observation. The fitted line shows a LOESS smooth of the data points, and the band around this line indicates a 95% confidence interval. Large daily outliers are not shown, but are included in all calculations.

While the age-sex measures of individual income (Figs 1–3) suggest that we might expect resource flows from middle-aged (high producing) adults to younger and elderly (lower producing) individuals, total HH income is determined by both the production profile of individuals and the age-sex composition of all HH members. Because income peaks in middle age, but number of dependent children also peaks in middle age, it is not simple to predict how per capita HH income (HH *need*) will change across the lifespan of married couples. A simple plot of mean HH net per capita income by mean age of HH heads (Fig 5) ranges just over two-fold and suggests that HHs comprised of young married couples around 30 years of age have slightly lower mean daily per capita income, and HH aged 50–65 years show greatest per capita incomes. It appears that household composition is sometimes adjusted across the lifespan, with related juveniles and adults joining or leaving HHs, such that per capita HH income is relatively steady across the lifespan for much of the community.

### Transfer of material goods in-to and out-of households

In-depth interviews, usually with the female head of a randomly-selected HH, on each day of fieldwork (493 interviews over 41 months) were conducted from 2015–2018 using a 24-hour recall to solicit information on all transferred *inflows* and *outflows* of material goods to/from every individual in each HH sampled. The primary interview question was: *“List any food and non-food items (resources) that you received/shared-out yesterday from/to anyone outside your HH (crops*, *fish*, *medicinal plants*, *money*, *etc*.*) and state the kin relationship if any to the donor/recipient”*. Importantly, the transfers of material goods were not considered “purchases,” which were listed separately in the HH interview. Transfers *in-to*, and *out-of* HHs included categories translated as “gifts” and “sharing,” which had no specified payment agreement or specified repayment date.

Interviews revealed that, on a daily basis, Linao HHs generally received almost 2/3 the value of goods from sharing each day (mean $4.48 per HH day) as the daily value of their own productive labor (mean = $6.97 per HH day). Importantly, however, only 66% of the value of *inflow* goods came from Linao residents (34% came from HHs outside the study village), but 88% of outflow value went to HHs in the Linao village. But, because we only have full information about characteristics of resident households, only the transfers between village co-residents are analyzed in the models presented here. Items received from Linao residents included food (46% of total value), money (43%), drugs and medicines (6%), clothing (3%), tools (1%), as well as smaller amounts of bait, adornments, water, boat fuel, firewood, soap, and dishes. Non-Linao residents mainly transferred money (60%) and food (29%) to Linao residents, but also shared small amounts of all other goods that are shared between co-residents.

Kin bias of inflow donors (including inflow from outside the community) was evident, with 50% of total inflow coming from very closely-related HHs (*r* = 0.5), 15% from closely-related HHs (0.5 > *r* > = 0.25), 4% from moderately-related HHs (0.25 > *r* > = 0.125), 3% from distantly-related HHs (0.125 > *r* > 0), 13% from unrelated individuals (*r* = 0), and 14% from donors whose relationship was unreported. Inflow of shared material goods from other HHs was reported by Linao residents of all ages, even small children. For example, about 16% of the money received, and 15% of the food received, from other HHs was transferred directly to children under the age of 15 years. The frequent transfer of money is both curious and notable, since money is highly storable and often shows no decreasing utility over the range of daily incomes observed here. There were 5,047 events of money sharing reported during the 493 HH sample days, with a mean transferred amount of $0.28 between donor and recipient. This included 2 large single transfers of about $20 (from a sister-in-law, and from a mother), but most transfer events (51%) consisted of $0.10 or less shared from donor to recipient on a single day. These small but frequent cash gifts may serve to renew and re-enforce alliances and cooperative bonds daily, rather than provide critical material support to recipients.

Reported daily *outflow* transfers of material goods to Linao HHs averaged somewhat less than mean daily inflow from Linao HHs. The mean value of items reported given away by HHs was valued at $1.82 per HH day, of which 88% was transferred to other Linao resident HHs. We are unsure why the reported mean value of outflow between co-residents in our sample is about $1.36 per day less than reported mean inflow over the same sample period. Possibly, female heads of households (who contributed the majority of our interviews) keep closer track of gifts received by their children and spouse than sharing out to other HHs. Also, it is possible that men don’t always fully report to their wives everything they share with other individuals each day. In any case, the composition of material goods transferred out to other Linao HHs was similar to the list of items reported received by HHs: food (45% of total value), money (39%), drugs and medicine (9%), clothes (5%), tools (1%), as well as smaller amounts of water, bait, adornments, soap, and firewood. Kin bias in outflow transfers was also evident, with 55% of the total value transferred to HHs related at *r* = 0.5, 22% to HHs related at 0.5 > *r* > = 0.25, 6% to HHs related at 0.25 > *r* > = 0.125, 4% to HHs related at 0.125 > *r* > = 0, 12% to unrelated individuals, and 2% to individuals of unknown relationship.

Prior empirical studies and theory suggest that five HH characteristics might be related to levels of giving and receipt, depending on the benefits that motivate transfers. First, we expect to find that higher income HHs give more if transfers function as assistance, signaling, reciprocity or trade. High-income HHs should receive less than other HHs if giving is mainly assistance, but might receive more if transfers are part of a reciprocity system. Second, higher income variance might be a motivator for greater participation in variance-reducing transfer systems. Third, the number of juveniles in a HH should modify the effect of income and relative need of that HH, because dependent juveniles increase both the costs of giving and the benefits of receipt. Fourth, age patterns of resource flows are likely, not only because age affects income and dependency ratios, but also because changes in reproductive value over the lifespan should motivate older individuals to transfer resources to younger kin with higher reproductive value. Finally, HHs with greater residential stability might more frequently be chosen as partners in reciprocity networks, since their consistent presence provides a better statistical guarantee of future sharing.

#### What types of households give more?

In order to determine what types of HHs give more than others, we analyzed variation in the total value of all goods given to all other HHs *on each interview day* as a function of several different donor HH characteristics, each of which was aggregated at one of three different time scales: 1) prior to day of sampling (6 month mean values); 2) over the whole study (long-term mean values); or 3) on the day of sampling. We developed Generalized Linear Models (GLMs) to test for the statistical effects of six variables with all other variables controlled: 1) income (on the day of the interview, and the six-month-prior mean fishing income); 2) coefficient of variation of daily income; 3) number of juveniles (< 18 yrs.) in the household; 4) mean age of heads of HH (husband and wife); 5) fraction of monthly censuses that HH members were present during study period; and, 6) value of all food and goods received on the same interview day. In the models, HH outflow (giving) on each interview day was the dependent variable. We eliminated all HHs with less than 3 interview days, as well as HH days on which interviews were lacking for one of the two income indicators (daily production, fishing monitoring). This resulted in a sample of 455 HH interview days of *outflow* transfers (giving) across 32 HHs, for which all 6 independent variables could be measured during the 41-month study period. For a daily HH observation *i*, the regression equation for this model is:

$$\mathrm{HH}\;\mathrm{Daily}\;\mathrm{Outflow}{}_{i}\;∼\;\beta_{0}+\beta_{1}(6‐\mathrm{Month}\;\mathrm{Mean}\;\mathrm{Fishing}\;{\mathrm{Income}}_{i})+\beta_{2}(\mathrm{Fishing}\;\mathrm{Income}\;\mathrm{Coefficient}\;\mathrm{of}\;{\mathrm{Variation}}_{i})+\beta_{3}\ln\;\left(\mathrm{HH}\;\mathrm{Daily}\;\mathrm{Production}\;{\mathrm{Income}}_{i}\right)+\beta_{4}(\mathrm{HH}\;\mathrm{Daily}\;{\mathrm{Inflow}}_{i})+\beta_{5}(\mathrm{HH}\;\mathrm{Number}\;\mathrm{of}\;\mathrm{Juvenile}\;{\mathrm{Consumers}}_{i})+\beta_{6}(\mathrm{Mean}\;\mathrm{Age}\;\mathrm{of}\;\mathrm{Heads}\;\mathrm{of}\;{\mathrm{HH}}_{i})+\beta_{7}{\left(\mathrm{Mean}\;\mathrm{Age}\;\mathrm{of}\;\mathrm{Heads}\;\mathrm{of}\;{\mathrm{HH}}_{i}\right)}^{2}+\beta_{8}{\left(\mathrm{Mean}\;\mathrm{Age}\;\mathrm{of}\;\mathrm{Heads}\;\mathrm{of}\;{\mathrm{HH}}_{i}\right)}^{3}+\beta_{9}(\mathrm{HH}\;\mathrm{Fraction}\;\mathrm{of}\;\mathrm{Censuses}\;\mathrm{Present})$$

Importantly, note that mean age of both heads of HH (husband and wife) is used here, rather than the age of either a male or female head of HH. Justification for this decision comes from the fact that there is no clear ethnographic reason for selecting one sex over the other to characterize age of head of HH. Using mean age of heads of HH also accounted for cases in which one spouse was deceased. Moreover, while male heads of HH are often older than female heads of HH in Linao, it is the female head of HH’s age which limits offspring reproduction due to cessation of reproductive capability at menopause. With these points in mind, we felt that mean age of both heads of HH best characterized overall age of a HH.

All variable values were standardized and measured in standard deviation units above or below the sample mean value for that variable. Our GLM models mainly presume linear associations (positive or negative slopes), but age and daily income are modeled as non-linear effects. Prior work, and human life tables, imply that age-specific family dependency ratios are inverse U shaped (e.g., [5]). However, resource production always falls in old age. Hence, we included second- and third-order polynomial age terms in our model to account for the influence of age on both dependency ratios and production, both of which may impact resource transfers. Likewise, we allowed for a marginally decreasing impact of greater income by taking the natural log of the daily income variable. LOESS smooths of these two variables plotted by daily HH outflow confirmed our speculation about the shape of these relationships (Fig J in S1 Text).

To control for HH-level variation, random and fixed effects for HH were also tested. However, these proved to be ineffective when controlling for long-term HH characteristics such as 6-month mean fishing income, mean age of heads of HH, and residential permanence (Table B(a), B(b) in S1 Text), and were therefore excluded from the final models. Similarly, we also explored using cross-lagged panel regression models (not shown) to account for temporal correlations between HH outflows; however, the frequent migration of HHs to locations outside of the study community for weeks or months at a time required that we aggregate data informing the cross-lagged panel models over long time spans (e.g. years). Given that the results of the cross-lagged panel models were consistent with long-term mean models of HH outflows (see below), we chose to include only the more parsimonious long-term mean versions of the models in this paper.

Initial exploration suggested that high day-to-day variation in HH giving cannot be fully explained unless *daily variation* in resource access (income and inflow from others) is considered in addition to long-term characteristics of the HH (Table A in S1 Text). Results (Table 2) showed that short-term (daily) income and receipt of goods on the interview day, as well as long-term fishing income and age, were indeed all significant predictors of daily amounts given. Number of juveniles in the HH was not significant; however, this variable is moderately colinear with mean age of heads of HH and is possibly not significant for that reason. The GLM accounts for about 35% of the variation in daily amount transferred out of a HH on any specific interview day (Table 2, adjusted *R*^*2*^ = 0.35). Plots derived from regression coefficients confirm that the age and daily income effects are significantly nonlinear (Fig K in S1 Text).

**Table 2:** GLM of daily giving (outflow) of *all goods* by HH characteristics.

Response variable: HH Daily Outflow of All Goods					
	**Estimate**	**SE**	***t*-value**	**P(>|*t*|)**	
(Intercept)	1.334	0.101	13.210	<0.00001	***
6-Month Mean Fishing Income	0.411	0.077	5.335	<0.00001	***
Fishing Income Coefficient of Variation	-0.051	0.073	-0.696	0.48659	
ln(Daily Production Income)	0.301	0.077	3.905	0.00011	***
HH Daily Inflow of All Goods	0.667	0.076	8.826	<0.00001	***
HH Number of Juvenile Consumers	0.056	0.083	0.674	0.50038	
Mean Age of Heads of HH	0.544	0.188	2.899	0.00393	**
(Mean Age of Heads of HH)^2^	0.380	0.107	3.563	0.00041	***
(Mean Age of Heads of HH)^3^	-0.201	0.073	-2.740	0.00640	**
HH Fraction of Censuses Present	0.111	0.078	1.413	0.15829	
*n* = 455 observations					
Residual *SE* = 1.486 with *df* = 445					
Adjusted *R*^*2*^ = 0.354					

Significance levels: 0 < *** < 0.001 < ** < 0.01 < * < 0.05 <. < 0.1. All independent variables are standardized. *SE* is standard error, and *df* is degrees of freedom.

In order to eliminate high day-to-day fluctuation in giving, we developed a GLM model to examine which types of HHs gave more on average across the entire sample period. For this analysis, we used a single measure of mean daily giving for each of the 32 HHs that were monitored for at least 3 sample days for all variables. The regression equation is equivalent to that of the daily model, except that it instead includes HH mean versions of all variables. Using the *mean* of fishing income and *mean* production income over the entire sample period (and coefficient of variation of fishing income), as well as *mean* daily receipt from others, *mean* number juvenile consumers, *mean* age of HH heads, and permanence of residence (% all censuses present), this model accounts for about 67% of the observed variation in mean HH giving over the sample period (Table 3, adjusted *R*^*2*^ = 0.67). However, mean amount received from other HHs, and one mean income measure (fish production), are the only significant predictors of mean amount transferred over the entire sample period. (The two income measures are moderately colinear (Fig G in S1 Text), hence only one is significant.) The large, significant intercept coefficient of this model implies that all HH gave a considerable amount on average, regardless of specific HH characteristics.

**Table 3:** GLM of HH *mean* daily outflow of *all goods* by HH characteristics.

Response variable: Mean HH Daily Outflow of All Goods					
	**Estimate**	**SE**	***t*-value**	**P(>|*t*|)**	
(Intercept)	1.527	0.175	8.735	<0.00001	***
Mean 6-Month Fishing Income	0.396	0.163	2.428	0.02380	*
Mean Fishing Income Coefficient of Variation	-0.015	0.106	-0.146	0.88553	
Mean ln(Daily Production Income)	0.577	0.354	1.630	0.11738	
Mean HH Daily Inflow of All Goods	0.868	0.232	3.738	0.00114	**
Mean HH Number of Juvenile Consumers	-0.156	0.165	-0.945	0.35493	
Mean Age of Heads of HH	0.149	0.348	0.429	0.67178	
(Mean Age of Heads of HH)^2^	0.108	0.156	0.693	0.49525	
(Mean Age of Heads of HH)^3^	-0.040	0.118	-0.336	0.73983	
Mean HH Fraction of Censuses Present	0.133	0.100	1.321	0.20018	
*n* = 32 observations					
Residual *SE* = 0.628 with *df* = 22					
Adjusted *R*^*2*^ = 0.665					

Significance levels: 0 < *** < 0.001 < ** < 0.01 < * < 0.05 <. < 0.1. All independent variables are standardized. *SE* is standard error, and *df* is degrees of freedom.

Since prior studies in small scale societies have focused mainly on food giving, we next examined whether patterns of food outflows are different from outflow of *money* and *other goods*. Resource-specific models were specified in exactly the same manner as the total resource models described above, except that the HH outflow and HH inflow variables were restricted to daily transfers of the resource of interest; for example, in the *food-only* model, the dependent variable was HH outflow of food resources, and the independent HH inflow variable was restricted to only inflows of food resources. The *food only* daily outflow transfers GLM (Table 4) shows the same significant predictor variables as the daily total outflow model (Table 2) and accounts for about the same amount of variance (adjusted *R*^*2*^ = 0.39 vs. 0.35). When we examine mean food outflow over the entire sample period for each HH, the food model explains more variance than the mean total resource outflow model, but fishing income is no longer a significant predictor of mean daily food outflow (Table C in S1 Text vs. Table 3, adjusted *R*^*2*^ = 0.85 vs. 0.67). HHs with higher total income, older HHs, and HHs that received more food over the sample period generally gave more food.

**Table 4:** GLM of daily outflow of *food* by HH characteristics.

Response variable: HH Daily Outflow of Food					
	**Estimate**	**SE**	***t*-value**	**P(>|*t*|)**	
(Intercept)	0.581	0.052	11.077	<0.00001	***
6-Month Mean Fishing Income	0.169	0.040	4.166	0.00004	***
Fishing Income Coefficient of Variation	-0.008	0.038	-0.204	0.83875	
ln(Daily Production Income)	0.095	0.040	2.387	0.01742	*
HH Daily Inflow of Food	0.280	0.041	6.846	<0.00001	***
HH Number of Juvenile Consumers	0.071	0.043	1.647	0.10034	
Mean Age of Heads of HH	0.352	0.097	3.612	0.00034	***
(Mean Age of Heads of HH)^2^	0.202	0.055	3.649	0.00029	***
(Mean Age of Heads of HH)^3^	-0.066	0.038	-1.725	0.08518	.
HH Fraction of Censuses Present	0.072	0.041	1.767	0.07799	.
*n* = 455 observations					
Residual *SE* = 0.772 with *df* = 445					
Adjusted *R*^*2*^ = 0.385					

Significance levels: 0 < *** < 0.001 < ** < 0.01 < * < 0.05 <. < 0.1. All independent variables are standardized. *SE* is standard error, and *df* is degrees of freedom.

When we examine daily transfers of *money* (Table 5), we find mostly the same significant predictor variables; however, age is not significant, and the money transfers model explains less variance than does the food sharing model (20% vs. 39%). Interestingly, the standardized coefficients of the two income measures are around 50% higher for giving money than for giving food (0.24 vs. 0.17, and 0.16 vs. 0.10). The model of mean HH outflow of money over the entire sample period accounts for considerably less variance than does the *food only* model (Table D in S1 Text, adjusted *R*^*2*^ = 0.53 vs. 0.85) but once again the standardized coefficient of total income on amount of money given is much higher than for food given (0.55 vs. 0.26). Apparently, household income has a stronger influence on who gives money than who gives food.

**Table 5:** GLM of daily outflow of *money* by HH characteristics.

Response variable: HH Daily Outflow of Money					
	**Estimate**	**SE**	***t*-value**	**P(>|*t*|)**	
(Intercept)	0.634	0.054	11.755	<0.00001	***
6-Month Mean Fishing Income	0.236	0.040	5.850	<0.00001	***
Fishing Income Coefficient of Variation	-0.082	0.039	-2.094	0.03683	*
ln(Daily Production Income)	0.157	0.041	3.773	0.00018	***
HH Daily Inflow of Money	0.201	0.039	5.169	<0.00001	***
HH Number of Juvenile Consumers	-0.037	0.045	-0.828	0.40791	
Mean Age of Heads of HH	0.094	0.101	0.938	0.34893	
(Mean Age of Heads of HH)^2^	-0.033	0.057	-0.577	0.56419	
(Mean Age of Heads of HH)^3^	-0.033	0.039	-0.832	0.40572	
HH Fraction of Censuses Present	0.068	0.042	1.619	0.10608	
*n* = 455 observations					
Residual *SE* = 0.796 with *df* = 445					
Adjusted *R*^*2*^ = 0.198					

Significance levels: 0 < *** < 0.001 < ** < 0.01 < * < 0.05 <. < 0.1. All independent variables are standardized. *SE* is standard error, and *df* is degrees of freedom.

Finally, when we examine daily transfers of *other goods* (clothes, medicine, tools, adornments, etc.), income measures are no longer important predictors of outflow. Instead, only age and value of other goods received from other HHs on the same interview day are associated with greater amounts of other goods” given away on a daily basis (Table 6). The model of daily sharing of other goods accounts for more variance than does the model of daily sharing of money (Table 5 vs. Table 6, adjusted *R*^*2*^ = 0.20 vs. 0.33), but the corresponding model of mean sharing of other goods over the entire sample period accounts for the least variance (only 29%) of any of our long-term models (Table E in S1 Text).

**Table 6:** GLM of daily outflow of *other goods* by HH characteristics.

Response variable: HH Daily Outflow of Other Goods					
	**Estimate**	**SE**	***t*-value**	**P(>|*t*|)**	
(Intercept)	0.103	0.047	2.186	0.02930	*
6-Month Mean Fishing Income	-0.019	0.036	-0.524	0.60030	
Fishing Income Coefficient of Variation	0.015	0.034	0.448	0.65447	
ln(Daily Production Income)	0.009	0.036	0.261	0.79421	
HH Daily Inflow of Other Goods	0.472	0.034	14.034	<0.00001	***
HH Number of Juvenile Consumers	0.062	0.039	1.581	0.11463	
Mean Age of Heads of HH	0.135	0.088	1.530	0.12666	
(Mean Age of Heads of HH)^2^	0.218	0.050	4.359	0.00002	***
(Mean Age of Heads of HH)^3^	-0.092	0.034	-2.673	0.00780	**
HH Fraction of Censuses Present	-0.048	0.037	-1.300	0.19431	
*n* = 455 observations					
Residual *SE* = 0.669 with *df* = 445					
Adjusted *R*^*2*^ = 0.331					

Significance levels: 0 < *** < 0.001 < ** < 0.01 < * < 0.05 <. < 0.1. All independent variables are standardized. *SE* is standard error, and *df* is degrees of freedom.

**Fig 6:**
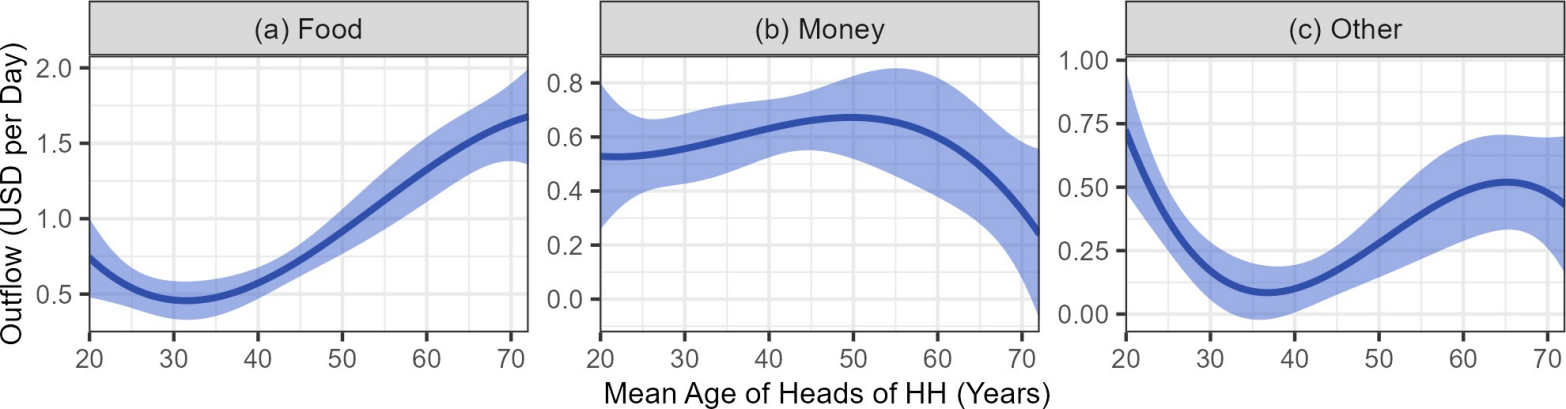
Outflow of food, money, and other goods by mean age of heads of HH. The shape (and 95% confidence interval) of the non-linear effect of mean age of HH heads on daily HH outflow for food (a), money (b), and other goods (c) according to the daily giving model. Independent age variables were converted back from standardized to raw variable values after prediction, and all other variables were set to the sample mean value.

Our analysis shows that age was only a significant predictor of amount given for food (*H*_0_: all age coefficients equal zero; partial *F* = 21.051, *p* < 0.00001) and other goods (*H*_0_: all age coefficients equal zero; partial *F* = 7.1577, *p* = 0.0001). However, the shape of the relationship appears different for food, money, and other goods (Fig 6A–6C). Plots of the polynomial age coefficients suggest that older HH give more food, but they are least likely to give money to others. Other goods were mainly given by younger and older HHs, but not by families of middle age. The giving patterns suggest a complex economic system with flows of different resource types between HHs of different ages, despite the fact that total giving is generally greater for younger and older HHs (Fig K in S1 Text). Indeed, the shape of per capita income by age (Fig 5) and outflow by age (Fig K in S1 Text) are strikingly similar.

#### What types of households receive more?

Total daily *inflow* was analyzed in a similar fashion to daily outflow, first examining effects of long-term HH traits and daily juvenile dependents (Table F in S1 Text), and then adding in short-term income and outflow variables to help account for some of the day-to-day fluctuation in receipt. Unlike outflow transfers, however, we had no *a priori* expectation about the shape of the relationship between income and daily receipt of material goods. If inflows are mainly economic assistance, they should be negatively associated with income. However, if inflows are mostly returned goods from reciprocity, then high-income HHs should receive more from others. Likewise, the relationship between inflow transfers and mean age of the heads of HH is unpredictable, because middle-aged parents have the highest dependent child burdens, but also higher incomes than younger or older parents. Because these relationships may be non-linear, we first performed a LOESS smooth of inflow by income and by age (Fig L in S1 Text). The results suggest that the income effect is generally positive, but the age relationship is U shaped with middle-aged HH receiving the least from others.

Our final GLM model of daily resource transfers into HHs includes mean fishing income over the past 6 months, variance in fishing income, mean age of HH heads (3^rd^ order polynomial; see previous section for a justification), and fraction of days present over the study period. We also control for daily fluctuation by adding in daily income (natural log of production on the day of the interview), number of juvenile consumers residing in HH on the interview day, and value of resources given away (outflow) on the day of the interview. The regression equation of the daily HH inflow model for each daily HH observation *i* was:

$$\mathrm{HH}\;\mathrm{Daily}\;\mathrm{Inflow}{}_{i}\;∼\;\beta_{0}+\beta_{1}(6‐\mathrm{Month}\;\mathrm{Mean}\;\mathrm{Fishing}\;{\mathrm{Income}}_{i})+\beta_{2}(\mathrm{Fishing}\;\mathrm{Income}\;\mathrm{Coefficient}\;\mathrm{of}\;{\mathrm{Variation}}_{i})+\beta_{3}\ln\;\left(\mathrm{HH}\;\mathrm{Daily}\;\mathrm{Production}\;{\mathrm{Income}}_{i}\right)+\beta_{4}(\mathrm{HH}\;\mathrm{Daily}\;{\mathrm{Outflow}}_{i})+\beta_{5}(\mathrm{HH}\;\mathrm{Number}\;\mathrm{of}\;\mathrm{Juvenile}\;{\mathrm{Consumers}}_{i})+\beta_{6}(\mathrm{Mean}\;\mathrm{Age}\;\mathrm{of}\;\mathrm{Heads}\;\mathrm{of}\;{\mathrm{HH}}_{i})+\beta_{7}{\left(\mathrm{Mean}\;\mathrm{Age}\;\mathrm{of}\;\mathrm{Heads}\;\mathrm{of}\;{\mathrm{HH}}_{i}\right)}^{2}+\beta_{8}{\left(\mathrm{Mean}\;\mathrm{Age}\;\mathrm{of}\;\mathrm{Heads}\;\mathrm{of}\;{\mathrm{HH}}_{i}\right)}^{3}+\beta_{9}(\mathrm{HH}\;\mathrm{Fraction}\;\mathrm{of}\;\mathrm{Censuses}\;\mathrm{Present})$$

Results (Table 7) show that long-term fishing income and age are positively associated with daily receipt, whereas income on interview day is negatively associated with daily receipt. Amount given to other HHs on the interview day is also positively associated with amount received on that day. Finally, the age effect appears complex, with daily receipt lowest in for couples in their 40-50s and higher for younger or older HHs (Fig M in S1 Text). The GLM model accounts for about 26% of the variation in amount received on any specific interview day (Table 7, adjusted *R*^*2*^ = 0.26).

**Table 7:** GLM of daily receiving (inflow) of *all goods* by HH characteristics.

Response variable: HH Daily Inflow of All Goods					
	**Estimate**	**SE**	***t*-value**	**P(>|*t*|)**	
(Intercept)	2.667	0.156	17.118	<0.00001	***
6-Month Mean Fishing Income	0.274	0.121	2.261	0.02425	*
Fishing Income Coefficient of Variation	-0.122	0.113	-1.080	0.28064	
ln(Daily Production Income)	-0.435	0.118	-3.673	0.00027	***
HH Daily Outflow of All Goods	1.096	0.124	8.826	<0.00001	***
HH Number of Juvenile Consumers	0.059	0.128	0.462	0.64409	
Mean Age of Heads of HH	-0.524	0.290	-1.807	0.07147	.
(Mean Age of Heads of HH)^2^	-0.001	0.166	-0.008	0.99337	
(Mean Age of Heads of HH)^3^	0.269	0.113	2.392	0.01718	*
HH Fraction of Censuses Present	0.033	0.121	0.276	0.78279	
*n* = 455 observations					
Residual *SE* = 2.282 with *df* = 445					
Adjusted *R*^*2*^ = 0.259					

Significance levels: 0 < *** < 0.001 < ** < 0.01 < * < 0.05 <. < 0.1. All independent variables are standardized. *SE* is standard error, and *df* is degrees of freedom.

As we did for outflow, we also examined which HH types show highest mean daily inflow amount over the entire sample period. Using the mean of production income and fishing income over the entire sample period (and coefficient of variation of fishing income), as well as mean daily shared outflow from the HH, mean number of juvenile consumers, mean age of HH heads, and permanence of residence, we find a moderate model fit (Table 8; adjusted *R*^*2*^ = 0.43). Mean daily sharing outflow was the only significant predictor of mean amount received by each HH over the entire sample period. In short, HHs that gave more over the sample period also received more on average over the entire sample period. This is congruent with scatterplots of inflow and outflow for each family (Fig H in S1 Text) and supports a reciprocity view of transfers.

**Table 8:** GLM of HH *mean* daily inflow of *all goods* by HH characteristics.

Response variable: Mean HH Daily Inflow of All Goods					
	**Estimate**	**SE**	***t*-value**	**P(>|*t*|)**	
(Intercept)	2.693	0.332	8.112	<0.00001	***
Mean 6-Month Fishing Income	-0.289	0.345	-0.839	0.41069	
Mean Fishing Income Coefficient of Variation	-0.114	0.201	-0.566	0.57709	
Mean ln(Daily Production Income)	-0.581	0.703	-0.826	0.41779	
Mean HH Daily Outflow of All Goods	2.196	0.587	3.738	0.00114	**
Mean HH Number of Juvenile Consumers	0.120	0.320	0.376	0.71040	
Mean Age of Heads of HH	-0.421	0.659	-0.639	0.52949	
(Mean Age of Heads of HH)^2^	0.043	0.301	0.143	0.88771	
(Mean Age of Heads of HH)^3^	0.173	0.222	0.780	0.44366	
Mean HH Fraction of Censuses Present	-0.084	0.198	-0.426	0.67422	
*n* = 32 observations					
Residual *SE* = 1.195 with *df* = 22					
Adjusted *R*^*2*^ = 0.434					

Significance levels: 0 < *** < 0.001 < ** < 0.01 < * < 0.05 <. < 0.1. All independent variables are standardized. *SE* is standard error, and *df* is degrees of freedom.

As before, we also divided inflow of resources into three sub-categories (food, money, other goods) in order to determine if they were transferred differently. Models within specific resource types are again equivalent to the previously-described models of total resource inflows, except that HH inflow and outflow variables are restricted to within resource type. The model of *food* receipt shows nearly the same set of significant predictor variables as the model including all resources (Table 9), and accounts for about the same amount of variance in daily receipt (adjusted *R*^*2*^ = 0.28 vs. 0.26). Importantly, HHs with higher long-term (but not daily) income, and HHs who share more food out on the interview day also received more food each interview day. The model of mean daily food receipt over the entire sample period (Table H in S1 Text) accounts for considerably more variance (adjusted *R*^*2*^ = 0.62), but with mean daily HH outflow of food as the only significant predictor of mean daily food inflow. This suggests that reciprocity, rather than assistance to needy HHs, is the main reason that Linao HHs transfer food.

**Table 9:** GLM of daily inflow of *food* by HH characteristics.

Response variable: HH Daily Inflow of Food					
	**Estimate**	**SE**	***t*-value**	**P(>|*t*|)**	
(Intercept)	1.189	0.071	16.711	<0.00001	***
6-Month Mean Fishing Income	0.221	0.054	4.063	0.00006	***
Fishing Income Coefficient of Variation	-0.057	0.051	-1.117	0.26450	
ln(Daily Production Income)	-0.072	0.054	-1.332	0.18351	
HH Daily Outflow of Food	0.409	0.060	6.846	<0.00001	***
HH Number of Juvenile Consumers	0.017	0.058	0.299	0.76498	
Mean Age of Heads of HH	-0.186	0.133	-1.404	0.16116	
(Mean Age of Heads of HH)^2^	0.025	0.076	0.336	0.73715	
(Mean Age of Heads of HH)^3^	0.115	0.051	2.252	0.02479	*
HH Fraction of Censuses Present	0.043	0.055	0.777	0.43757	
*n* = 455 observations					
Residual *SE* = 1.038 with *df* = 445					
Adjusted *R*^*2*^ = 0.277					

Significance levels: 0 < *** < 0.001 < ** < 0.01 < * < 0.05 <. < 0.1. All independent variables are standardized. *SE* is standard error, and *df* is degrees of freedom.

In contrast to the food model, when we examine daily inflow of *money*, we find that daily income is *negatively* associated with daily receipt of cash (Table 10). But, total value of goods transferred out on the day of the interview is also associated with higher daily HH receipt of money. Hence, these two patterns suggests that both helping and reciprocity are involved in the patterns of money transfers in Linao. Again, the long-term model shows that only mean daily HH outflow of money is statistically associated to mean receipt of money over the whole sample period (Table I in S1 Text). Importantly, however, mediocre model fits for both the daily and long-term models (adjusted *R*^*2*^ = 0.11 and *R*^*2*^ = 0.31, respectively) suggest that we have not clearly identified the factors lead to variation in how much money is received by different HHs in Linao.

**Table 10:** GLM of daily inflow of *money* by HH characteristics.

Response variable: HH Daily Inflow of Money					
	**Estimate**	**SE**	***t*-value**	**P(>|*t*|)**	
(Intercept)	1.038	0.108	9.571	<0.00001	***
6-Month Mean Fishing Income	0.018	0.084	0.213	0.83120	
Fishing Income Coefficient of Variation	-0.078	0.080	-0.983	0.32603	
ln(Daily Production Income)	-0.333	0.083	-3.986	0.00008	***
HH Daily Outflow of Money	0.427	0.083	5.169	<0.00001	***
HH Number of Juvenile Consumers	0.131	0.090	1.459	0.14521	
Mean Age of Heads of HH	-0.106	0.203	-0.524	0.60025	
(Mean Age of Heads of HH)^2^	0.120	0.115	1.046	0.29602	
(Mean Age of Heads of HH)^3^	0.088	0.079	1.118	0.26415	
HH Fraction of Censuses Present	-0.048	0.085	-0.567	0.57094	
*n* = 455 observations					
Residual *SE* = 1.606 with *df* = 445					
Adjusted *R*^*2*^ = 0.109					

Significance levels: 0 < *** < 0.001 < ** < 0.01 < * < 0.05 <. < 0.1. All independent variables are standardized. *SE* is standard error, and *df* is degrees of freedom.

Finally, when we examine inflow of *other goods*, several variables are associated with receiving more (Table 11). As we found for food, greater long-term income and daily giving of other goods is also associated with greater daily receipt of other goods. Number of juvenile consumers is also a significant predictor, while mean age of HH heads is marginally significant. In the long-term model, number of juvenile consumers is statistically associated with receipt of other goods over the sample period (Table J in S1 Text), while mean daily giving is only marginally significant; but, surprisingly, both daily and mean number of juvenile consumers are negatively associated with amount received (daily and over the sample period).

**Table 11:** GLM of daily inflow of *other goods* by HH characteristics.

Response variable: HH Daily Inflow of Other Goods					
	**Estimate**	**SE**	***t*-value**	**P(>|*t*|)**	
(Intercept)	0.395	0.044	8.919	<0.00001	***
6-Month Mean Fishing Income	0.112	0.033	3.409	0.00071	***
Fishing Income Coefficient of Variation	0.001	0.032	0.035	0.97205	
ln(Daily Production Income)	<0.001	0.033	-0.006	0.99505	
HH Daily Outflow of Other Goods	0.439	0.031	14.034	<0.00001	***
HH Number of Juvenile Consumers	-0.076	0.036	-2.092	0.03702	*
Mean Age of Heads of HH	-0.158	0.082	-1.927	0.05459	.
(Mean Age of Heads of HH)^2^	-0.090	0.047	-1.912	0.05647	.
(Mean Age of Heads of HH)^3^	0.049	0.032	1.536	0.12534	
HH Fraction of Censuses Present	0.061	0.034	1.785	0.07500	.
*n* = 455 observations					
Residual *SE* = 0.649 with *df* = 445					
Adjusted *R*^*2*^ = 0.328					

Significance levels: 0 < *** < 0.001 < ** < 0.01 < * < 0.05 <. < 0.1. All independent variables are standardized. *SE* is standard error, and *df* is degrees of freedom.

**Fig 7:**
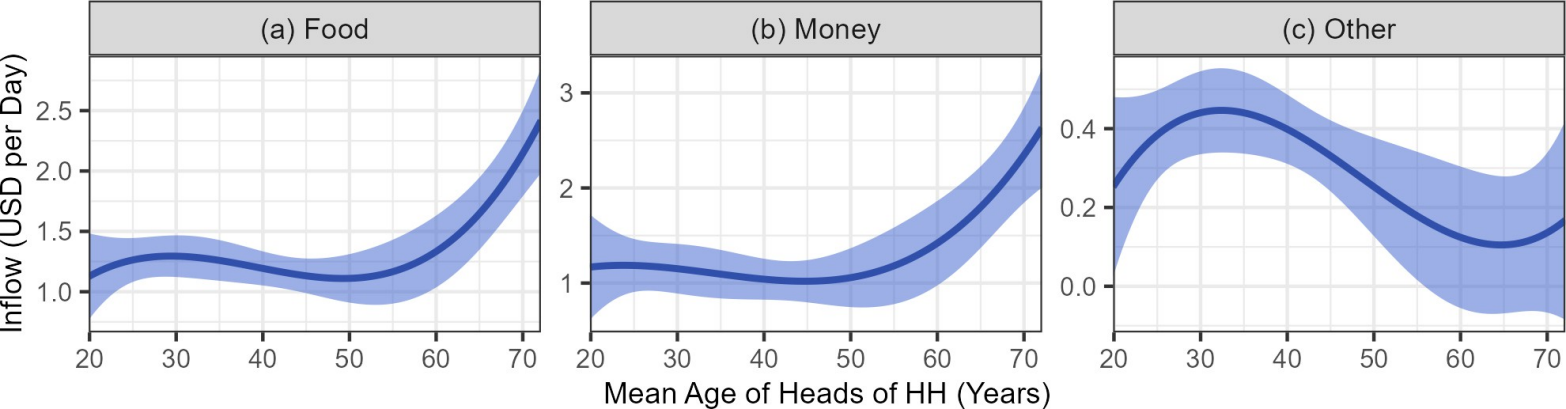
Inflow of food, money, and other goods by mean age of heads of HH. The shape (and 95% confidence interval) of the non-linear effect of mean age of HH heads on daily HH inflow for food (a), money (b), and other goods (c) according to the daily receipt model. Independent age variables were converted back from standardized to raw variable values after prediction, and all other variables were set to the sample mean value.

When the results of the food, money and other goods models are compared, it becomes clear that, just as we found for giving patterns, the age patterns of receipt vary considerably depending on resource type (Fig 7A–7C) and are only significantly associated with food and other goods. Older HH heads appear to receive more food (and money, albeit not significant), but young/middle aged HH heads received more of the other goods each day.

### Sharing between specific household dyads

Despite the fact that goods are transferred between many different HHs every day in the Linao community, some HHs appear to share more with specific other HHs in the village. Here, we develop two sets of models from our independently collected inflow and outflow databases to determine which types of HH Y give more goods to focal HH X, and which types of HH Y receive more goods from focal HH X. Because we are interested in kinship, reciprocity, and “need” as likely motivators for transfers, our models include the following variables: 1) genetic relatedness between HH X and HH Y (*r*_*HH*_, i.e. the coefficient of genetic relatedness for the two most closely related adults in each HH pair); 2) reciprocal inflow/outflow, to or from HH X and to or from HH Y, over the entire sample period and on the interview day; 3) approximate physical distance (meters) between HH X and HH Y; 4) per capita mean daily income *difference* between HH X and HH Y (rich vs. poor) over the whole sample period; 5) difference in mean age of HH heads between HH X and HH Y; and 6) the interaction term between genetic relatedness and income difference between HH X and HH Y (needy kin vs. not needy or not kin).

#### What are characteristics of households Y that gave more to household X?

The regression equation for the model of daily dyadic inflow observations *i* from a given HH Y to a focal HH X was as follows:

$$\mathrm{Daily}\;\mathrm{Inflow}\;\mathrm{to}\;X\;\mathrm{from}\;Y_{i}\;∼\;\beta_{0}+\;\beta_{1}(\mathrm{Mean}\;\mathrm{Daily}\;\mathrm{Inflow}\;\mathrm{into}\;X)+\;\beta_{2}(\mathrm{HH}\;\mathrm{Coefficient}\;\mathrm{of}Relatedness,X\&Y)+\;\beta_{3}(\mathrm{Daily}\;\mathrm{Outflow}\;\mathrm{from}\;X\;\mathrm{to}\;Y_{i})+\;\beta_{4}(\mathrm{Mean}\;\mathrm{Daily}\;\mathrm{Outflow}\;\mathrm{from}\;X\;\mathrm{to}\;Y)+\;\beta_{5}(\mathrm{Distance}\;\mathrm{Between}\;\mathrm{Houses},X\;\mathrm{to}\;Y_{i})+\;\beta_{6}(\mathrm{Difference}\;\mathrm{in}\;\mathrm{Mean}\;\mathrm{Production}\;\mathrm{Income},Y‐X)\;+\;\beta_{7}(\mathrm{Difference}\;\mathrm{in}\;\mathrm{Mean}\;\mathrm{Age}\;\mathrm{of}\;\mathrm{Heads}\;\mathrm{of}\;\mathrm{HH},Y‐X)+\;\beta_{8}(\mathrm{HH}\;\mathrm{Relatedness},X\&Y)\times (\mathrm{Mean}\;\mathrm{Production}\;\mathrm{Income}\;\mathrm{Difference},Y‐X)$$

Results suggest that focal HH X received more from any HH Y on a specific day if HH Y was more closely related, if HH X also gave more to HH Y on the interview day or HH X gave more to HH Y on average over the entire sample period (reciprocity), if HH Y had a larger mean per capita income than HH X over the sample period, if the two HH were closer together in space, and if HH Y was older than HH X (Table 12A). The effect of income difference (need) is greater when HH X and HH Y are also more closely related (interaction term). This model accounts for about 12% of the variance in daily transfers from different village HHs to focal HH X on any single interview day (adjusted *R*^*2*^ = 0.12). Importantly, even though giving by HH X to HH Y on the interview day and over the whole sample period are correlated (Pearson’s *r* = 0.52), the model allows us to detect the effect of sharing today vs. long-term sharing with sharing on each time scale statistically controlled. Similar standardized coefficients (Table 12A, 0.075 vs. 0.091) suggest that giving to HH Y today and giving to HH Y over the long run equally influence whether HH X will receive something from HH Y on any specific interview day. Finally, when we model the mean daily transfer amount from HH Y to HH X over the entire sample period (one data point per dyad), we eliminate the effect of daily reciprocity, but still discover the same set of significant independent variables (other than HH distance; Table 12B, adjusted *R*^*2*^ = 0.38). Since the standardized coefficients of short-term giving on daily receipt and long-term giving on long-term receipt are similar in the two models (Table 12A vs. 12B), we conclude that HHs Y give more to HHs X that give more to them today, and also give more to HHs X that have given more to them on average over long time periods.

**Table 12:** GLM of daily inflow of *all goods* to HH X from HH Y. **a. b.** GLM of mean inflow of *all goods* over sample period to HH X from HH Y.

Response variable: Daily Inflow of All Goods to X from Y					
	**Estimate**	**SE**	***t*-value**	**P(>|*t*|)**	
(Intercept)	0.108	0.005	23.095	<0.00001	***
Mean Daily Inflow of HH X	0.031	0.005	6.315	<0.00001	***
Coefficient of Relatedness, XY	0.062	0.005	11.310	<0.00001	***
Daily Outflow of All Goods, X to Y	0.075	0.005	13.698	<0.00001	***
Mean Daily Outflow of All Goods, X to Y	0.091	0.006	14.531	<0.00001	***
Distance Between Houses, X to Y	-0.016	0.005	-3.276	0.00106	**
Difference in Mean Production Income, Y-X	0.016	0.005	3.263	0.00111	**
Difference in Mean Age of Heads of HH, Y-X	0.001	<0.001	4.535	0.00001	***
Relatedness × Income Difference, Y—X	0.026	0.005	5.264	<0.00001	***
*n* = 12123 observations					
Residual *SE* = 0.514 with *df* = 12114					
Adjusted *R*^*2*^ = 0.121					
**Response variable: Mean Daily Inflow of All Goods to X from Y**					
	**Estimate**	**SE**	***t*-value**	**P(>|*t*|)**	
(Intercept)	0.093	0.007	13.781	<0.00001	***
Mean Daily Inflow of HH X	0.035	0.007	4.758	<0.00001	***
Coefficient of Relatedness, XY	0.064	0.008	8.181	<0.00001	***
Mean Daily Outflow of All Goods, X to Y	0.111	0.008	13.908	<0.00001	***
Mean Distance Between Houses, X to Y	-0.009	0.007	-1.346	0.17862	
Difference in Mean Production Income, Y-X	0.021	0.007	2.946	0.00330	**
Difference in Mean Age of Heads of HH, Y-X	0.001	<0.001	2.957	0.00318	**
Relatedness × Mean Income Difference, Y-X	0.030	0.007	4.319	0.00002	***
*n* = 965 observations					
Residual *SE* = 0.211 with *df* = 957					
Adjusted *R*^*2*^ = 0.375					

Significance levels: 0 < *** < 0.001 < ** < 0.01 < * < 0.05 <. < 0.1. All independent variables are standardized. *SE* is standard error, and *df* is degrees of freedom.

#### What are characteristics of households Y that received more from household X?

The model of daily dyadic outflow observations *i* from HH X to HH Y was specified as follows:

$$\mathrm{Daily}\;\mathrm{Outflow}\;\mathrm{from}\;X\;\mathrm{to}Y_{i}\;∼\;\beta_{0}+\;\beta_{1}(\mathrm{Mean}\;\mathrm{Daily}\;\mathrm{Inflow}\;\mathrm{into}\;Y)+\;\beta_{2}(\mathrm{HH}\;\mathrm{Coefficient}\;\mathrm{of}\;\mathrm{Relatedness},X\&Y)+\;\beta_{3}(\mathrm{Daily}\;\mathrm{Inflow}\;\mathrm{to}\;X\;\mathrm{from}\;Y_{i})+\;\beta_{4}(\mathrm{Mean}\;\mathrm{Daily}\;\mathrm{Inflow}\;\mathrm{to}\;X\;\mathrm{from}\;Y)+\;\beta_{5}(\mathrm{Distance}\;\mathrm{Between}\;\mathrm{Houses},X\;\mathrm{to}Y_{i})+\;\beta_{6}(\mathrm{Difference}\;\mathrm{in}\;\mathrm{Mean}\;\mathrm{Production}\;\mathrm{Income},X‐Y)\;+\;\beta_{7}(\mathrm{Difference}\;\mathrm{in}\;\mathrm{Mean}\;\mathrm{Age}\;\mathrm{of}\;\mathrm{Heads}\;\mathrm{of}\;\mathrm{HH},X‐Y)+\;\beta_{8}(\mathrm{HH}\;\mathrm{Relatedness},X\&Y)\times (\mathrm{Mean}\;\mathrm{Production}\;\mathrm{Income}\;\mathrm{Difference},X‐Y)$$

Results from the analysis of giving by HH X confirm the same trends that we saw in the receiving patterns above. There is a significant tendency for interview HH X to give more to HH Y if HH Y is more closely related, if HH Y gives more to HH X on the interview day or on average over the entire sample period, and if HH Y has lower income than HH X (Table 13A). Once again, the effect of income difference is greater when the HHs are more closely related (interaction term). The daily GLM with standardized variables accounts for about 14% of the variance in which types of HH Y are more likely to receive goods from the focal HH X on any single interview day (Table 13A, adjusted *R*^*2*^ = 0.14) and 39% of the variance in how much HH X gave on average to specific types of HH Y over the entire sample period (Table 13B, adjusted *R*^*2*^ = 0.39).

**Table 13:** GLM of daily outflow of *all goods* from HH X to HH Y. **a. b.** GLM of mean outflow of *all goods* over sample period from HH X to HH Y.

Response variable: Daily Outflow of All Goods from X to Y					
	**Estimate**	**SE**	***t*-value**	**P(>|*t*|)**	
(Intercept)	0.059	0.002	27.112	<0.00001	***
Mean Daily Inflow of HH Y	0.004	0.002	1.574	0.11546	
Coefficient of Relatedness, XY	0.028	0.003	10.822	<0.00001	***
Daily Inflow of All Goods, X from Y	0.033	0.002	13.412	<0.00001	***
Mean Daily Inflow of All Goods, X from Y	0.053	0.003	19.200	<0.00001	***
Distance Between Houses, X to Y	-0.008	0.002	-3.698	0.00022	***
Difference in Mean Production Income, X-Y	0.011	0.002	5.017	<0.00001	***
Difference in Mean Age of Heads of HH, X-Y	0.001	<0.001	4.300	0.00002	***
Relatedness × Income Difference, X-Y	0.020	0.002	8.671	<0.00001	***
*n* = 12123 observations					
Residual *SE* = 0.237 with *df* = 12114					
Adjusted *R*^*2*^ = 0.141					
**Response variable: Mean Daily Outflow of All Goods from X to Y**					
	**Estimate**	**SE**	***t*-value**	**P(>|*t*|)**	
(Intercept)	0.053	0.003	15.924	<0.00001	***
Mean Daily Inflow of HH Y	0.007	0.004	2.133	0.03320	*
Coefficient of Relatedness, XY	0.034	0.004	9.116	<0.00001	***
Mean Daily Inflow of All Goods, X from Y	0.054	0.004	14.455	<0.00001	***
Mean Distance Between Houses, X to Y	-0.009	0.003	-2.766	0.00578	**
Difference in Mean Production Income, X-Y	0.013	0.003	3.806	0.00015	***
Difference in Mean Age of Heads of HH, X-Y	<0.001	<0.001	1.516	0.12976	
Relatedness × Mean Income Difference, X-Y	0.022	0.003	6.421	<0.00001	***
*n* = 965 observations					
Residual *SE* = 0.103 with *df* = 957					
Adjusted *R*^*2*^ = 0.394					

Significance levels: 0 < *** < 0.001 < ** < 0.01 < * < 0.05 <. < 0.1. All independent variables are standardized. *SE* is standard error, and *df* is degrees of freedom.

### Linao beliefs about givers and receivers

After data collection for the study was complete, we interviewed 12 men and 24 women concerning what types of HHs they believed gave more or received more material goods during our study period. Questions were phrased to elicit a yes/no answer, and we calculated the proportion of *yes* answers for each trait. Traits included distance of HH from shore, HH age, HH income, size of boat, number of children, residential permanence, income variance, and whether the informant believed that high givers also received more (reciprocity), or that high givers received less (helping). Finally, we asked whether HHs that reported high or low inflows in the community were simply less truthful than other HHs. Results are shown in Table 14, where we present the proportion of *yes* answers for each question, as well as Wilson Score 95% confidence intervals with the Yates continuity correction for each proportion. Interestingly, a high proportion (around 80%) of informants believed that HHs who reported giving or receiving more than other HHs in our interviews were being less truthful than other HHs. This is something that has not been considered in prior studies of sharing based on informant reports. For each question, if the lower 95% confidence interval for a *yes* answer was >50%, we considered the community answer to be YES, and if the upper 95% confidence interval was <50%, we considered the community answer to be NO. Questions for which the confidence intervals overlapped the 50^th^ percentile were considered to indicate lack of community agreement (shown in Table M in S1 Text).

**Table 14:** Linao opinions about types of HHs that give or receive more.

Which HHs Give More?				
**Question**	**N**	**Proportion Yes**	**95% Lower CI**	**95% Upper CI**
HHs who receive the most	36	0.861	0.697	0.948
HHs being less truthful	36	0.778	0.604	0.893
HHs who reside more permanently	36	0.708	0.531	0.842
HHs with older heads of household	36	0.681	0.503	0.820
HHs who receive less on average	35	0.271	0.142	0.451
HHs with younger heads of household	36	0.250	0.127	0.425
HHs with variable daily income	36	0.236	0.117	0.411
HHs with many small children	36	0.222	0.107	0.396
HHs who reside less permanently	36	0.153	0.061	0.319
**Which HHs Receive More?**				
**Question**	**N**	**Proportion Yes**	**95% Lower CI**	**95% Upper CI**
HHs with older heads of household	36	0.889	0.730	0.964
HHs who reside more permanently	36	0.861	0.697	0.948
HHs with smaller boats	36	0.806	0.634	0.912
HHs with lower average income	36	0.792	0.619	0.902
HHs with variable daily income	36	0.778	0.604	0.893
HHs being less truthful	35	0.743	0.564	0.869
HHs with few small children	36	0.292	0.158	0.469
HHs who give the most receive less	36	0.278	0.148	0.454
HHs who reside less permanently	36	0.181	0.079	0.350
HHs with higher average income	36	0.111	0.036	0.270
HHs with larger boats	36	0.111	0.036	0.270
HHs with steady daily income	36	0.111	0.036	0.270
HHs with younger heads of household	36	0.083	0.022	0.236

Upper and lower 95% confidence intervals bounds are calculated as Wilson Score intervals with the Yates continuity correction.

Interview results suggest that Linao informants believe that high receiving, more permanent, and older HHs with fewer children generally give more goods than other HHs. They also believe that low receiving, younger HHs, HHs with higher income variance, HHs with more children, and less permanent HHs generally give less than other HHs.

Likewise, the opinions about receiving indicate that Linao informants believe that older HHs, more permanent HHs, HHs with smaller boats, many children, or high income variance receive the most from HH transfers. Village HHs that are less permanent, with higher income, fewer children, lower income variance, younger heads of HH, and larger boats are believed to not receive as much as others.

Finally, we asked informants to freely nominate traits of high givers and high receivers and to identify them in the community. Most commonly listed traits for high-giving HHs included *generous* (18 mentions), *few children* (9 mentions), and *older* (5 mentions). The most frequently mentioned high-giving HH (64% of all interviews) were, in order: a) A childless couple in their early 50s, with modest income; b) an elderly couple in their early 70s who are raising two orphaned teen grandchildren, have an unmarried middle-aged son, and own of a small store; c) the daughter of the original village chief, who became chief mid-study after her father’s death and is 40 years old, married with two teenage children, and has a permanent salaried job in a nearby tuna factory.

Most commonly listed traits for high-receiving HHs included *low income* (15 mentions), *many children* (5 mentions), and *shares a lot with others* (3 mentions). The most frequently listed highest recipients (72% of all interviews) were, in order: a) a middle-aged couple in their 50s with a very low income, who have 10 born children (only five surviving), with three living at home including one handicapped teen; b) the 70-year-old blind and partially-disabled widowed wife of the first village chief; c) a single 35-year-old widowed mother of two older children; d) a couple in their mid-30s with 7 dependent children (one disabled) living at home.

The Linao beliefs about sharing therefore emphasize the relative wealth (per capita income) and poverty of HHs as major determinants of high giving and high receiving, rather than focusing on HHs involved in high reciprocal flows of material goods. Perhaps Linao residents don’t really consider HH to be *givers* when they frequently receive back goods in return, and they don’t consider HH to be *receivers* when they also give away large quantities of goods.

## Discussion and conclusion

Our 26 statistical models (Table N in S1 Text) of resource transfers suggest a variety of factors that lead to increased giving or receiving. First, we can explain who gives better than we can explain who receives (models of giving always have higher adjusted *R*^*2*^ values than do similar models of receiving). Second, models of food transfers explain considerably more than do models of money transfer or other goods. Particularly puzzling is the almost daily transfer of very small sums of cash money to and from individuals of all age and sex groups. In general, income and age are consistent predictors of daily outflows and inflows. Importantly, high income predicts both increased outflow and increased inflow. Levels of daily outflow and inflow are also significantly correlated in all models. These two results suggest that reciprocity is the major incentive driving resource transfer and differences in outflows and inflows between study HHs. Age effects are also frequently significant in our models, but non-linear and complicated (Figs K, M, P in S1 Text). We suggest that HHs can be divided into 4 groups: young couples, middle-aged couples, old couples, and elderly. Outflows are highest from young and old HHs, and inflows are highest to middle-aged and elderly HHs. Age patterns also vary by resource type, with old and elderly HHs giving more food, but giving less money. This is not surprising, since younger individuals are more likely to be involved in commerce or wage labor. Finally, reciprocity in the Linao village can be detected at different time scales. HH X tends to receive more from HH Y today if HH X gave more to HH Y today, but also if HH X gives more HH Y during the entire 3-year study period. But, the daily reciprocal transfers do not appear to be trade or commercial exchange, since the value given and received by HH dyads on most interview days is highly disparate (Fig R in S1 Text) and only tends to balance out over longer time periods (Fig S in S1 Text).

This is the first study we are aware of that tabulates the transfer of **all** material goods between all households in a small-scale society (village) over a fairly long observation period (> 3 years). The major findings are that: 1) Daily resource transfers between HHs are diverse and extensive, representing a value equal to about 2/3 of the daily income of most households. Food transfers constitute by far the most valuable category of sharing. 2) The interaction term between income differences and coefficient of relatedness shows that HHs are particularly likely to give to needy kin (rather than needy non-kin). This is the best evidence that we have of transfers that might be considered “helping,” rather than reciprocity. However, the standardized coefficients of the dyadic transfers models suggest that reciprocity is more important than income differences or even kinship in determining who gives and gets shares from the focal HH. 3) Kinship is important, not only for assistance (see kinship x need interaction effects), but especially for establishing reciprocal cooperation. Indeed, the effects (standardized coefficients) of reciprocity on giving and receiving are almost as high for close and distant kin subsets as they are for the non-kin subset of HH in our study (see Tables O-T in S1 Text). This resembles results reported by Allen-Arave, Gurven, and Hill [35], in which food transfers among Ache villagers in Paraguay were more reciprocal among kin than among non-kin. These two findings appear to contradict the evolutionary view that giving to kin is mainly motivated by inclusive fitness, whereas non-kin giving is mainly explained by reciprocal altruism. Instead, most kin giving also appears to constitute reciprocity. Importantly, we note that kin terms were frequently used to justify reciprocal transfers reported in our interviews. For example, recipient HHs reported donors from 68 different types of *distant kin* (with kinship specified up to 7 links away from ego; Table U in S1 Text) and from 146 types of *distant affines* (not including close affines, with relationships specified up to 7 links from the interview subject; Table V in S1 Text). This appears consistent with ethnographic reports that the Sama recognize an extensive group of personal kindred called “*dakampungan*,” with whom they are supposed to behave equally cooperatively even though they are not closely genetically-related [30 pp127]. Cooperation between distant kin is an important theoretical issue in human cooperation. Recognized kinship, with specified but very distant relationships, may stabilize reciprocity (lowering rates of defection?) even when genetic coefficients of relatedness between interactants are near zero.

Our study suggests that reciprocal sharing is overwhelmingly the most important factor in determining how much different HHs transfer out or receive as *inflows* on a daily basis. Likewise, when we examine dyadic relationships, reciprocity appears more important than either kinship or need differences in determining flows. This is congruent with recent reviews of primate and human food sharing patterns in other small-scale societies [16], which also suggest that people are more motivated by gains from cooperative reciprocity than by other factors. It will be important to determine if this view holds up as more empirical studies of all resource transfers between HHs in small scale societies become available.

## Supporting information

S1 Checklist Inclusivity in global research. (DOCX)

S1 Text Supplementary online information. (DOCX)
