# Supplementary material for: Inter-household transfers of material goods among Sama “sea nomads” of the Philippines: Reciprocity, helping, signaling, or something else?
Source: PLoS One. 2023 Aug 24;18(8):e0290270. doi: 10.1371/journal.pone.0290270 (PMC10449209; doi:10.1371/journal.pone.0290270)
Supplement: S1 Text — (DOCX) [file pone.0290270.s002.docx]

**Supplementary Online Materials**

*Note that references cited in this SOM are given at the end of the document and use a different numbering scheme from that of the main text.*

**Ethnographic Background**

The study community, called *Linao of the Mangroves* was established progressively in the 1980s-90s on the coast of Southern Mindanao (Sarangani Province), by a growing community of predominantly ethnic Sama tribal people (called *Badjau* or “sea nomads” by Filipinos). The Sama ethnic population now contains about 1.1 million individuals that inhabit regions of coastal and shallow reef habitat in the Celebes Sea (Borneo, Mindanao, Suluwesi, West Papua), and south to the Timor Island region [1,2]. The Sama that live in Mindanao and the Sulu Archipelago converted to Islam prior to European colonization of the Philippines, and have intermarried with members of the Tausug (farmer) ethnic group of the same region. Families in our study community came originally from Kabinga’an Island in the Siasi island group (located in the Sulu Archipelago), Sabah (Malaysia), and the western coast of Mindanao, and moved to southern Mindanao during the civil unrest period of the 1970s. The Sama people of Southwestern Mindanao were observed and described by Magellan’s crew in 1521 as nomadic sea-dwellers, living on houseboats. They fished and collected sea cucumbers for trade, but had no crops or domestic animals.

Our study population all spoke Sama as their first language, with Tausug also spoken by a minority of adults (4 of the adults in the study population primarily spoke Tausug as a child), and Binisaya, the Southern Philippine national language, is spoken fluently by most adults and children. 10% of the parents of the adults now living in Linao self-identified as mainly Tausug, and 40% were reportedly culturally mixed (from prior generations) Sama/Tausug. Interviews for this study were conducted in Binisaya, but answers were written directly in English and then coded into a digital database by Filipino student research assistants from Mindanao State University (co-authors here).

The Linao study population in Sarangani Bay is surrounded directly by ethnic Blaan and Maguindanaon tribal people, as well as Filipino nationals (Binisaya speakers). Slightly inland, there are also T’boli and Manobo tribal peoples, and the location of the famed “Tasaday” tribe of isolated hunter-gatherers is approximately 50 km to the northwest. Other ethnic Sama speakers are located in multiple discrete settlements at least 10 km from Linao, and across a broad range of the Sarangani coast. The most commonly-visited communities of Linao relatives were located approximately 30 km (Lumatil), 50 km (Silway), 100 km (Digos), and 200 km (Bgy. Tibunco, Davao) away. Linao inhabitants interact to a moderate extent with non-Sama ethnic groups, but rarely intermarry with them (there were only 3 male, and 0 female, non-Sama spouses of the 70 or so married families resident in Linao during our study period).

The Linao village is built on a reef flat just off the coast in Sarangani Bay, near the highway that runs from General Santos City to Kiamba. The coastal land area near Linao rises steeply to form a first ridge of dry tree-scrub hills inland about 1 km. Further inland and crossing a series of ridges parallel to the coast, the mountains rise and eventually turn tropical and humid, covered with rainforest. But, the immediate coast and hillside adjacent to Linao are situated in a rain shadow that is too dry for farming (e.g. rice, bananas, root crops) or even planting coconuts. Hence, no farming villages are located nearby and Linao residents plant nothing, instead trading marine resources for edible carbohydrates. Weather patterns fluctuate slightly during the year, with March-May as the driest, “calm” season of the year, while July-October are marked by heavy rainfall and tropical typhoon-like storms that can create dangerous waves which occasionally damage community living structures.

From June 2015 to November 2018, the Linao study community consisted of 25-40 thatched huts connected by a crude wooden walkway system built on tree posts about 2 meters above the ocean. Houses were built out over mangrove/reef flats (submerged except during lowest tides) in Sarangani Bay, in order to gain quick access to nearby fishing sites, reefs, and intertidal foraging grounds. The community did not have legal land title (it is technically on the ocean), but its long-term presence has politically legitimized the settlement (the coastal beachfront is titled to the Blaan tribal population, the original inhabitants of Sarangani Bay). The intertidal reef flats contain about 5 hectares of exploitable coral reef, mangrove mud flats, and seagrass beds that contain a large variety of edible marine invertebrate species. During our study period, 58 invertebrate species were collected, but 84% of the intertidal food-energy/trade-value came from only 5 species. Most commonly harvested are sea slug eggs, medium-sized bivalves, sea urchin eggs, and small gastropods. In nearby communities, sea cucumbers are harvested for trade, but these are not common in Linao. Beyond the reef, but usually within sight of the community, fishing activities employ both small surface float-net technology (3-5 men in a boat within a couple kilometers of the settlement) and hook-and-line methods (1-3 men per boat, usually within a kilometer or less).

The mean village size of Linao during the sample period was 113 persons, but ranged from 87-149 persons on monthly census days. Some village families left Linao for weeks or even months at a time, and then returned at a later date, whereas about half of the HHs were permanent residents throughout the entire study period (i.e., present on at least 80% of census days). From census data we estimate that adults (>=18 yr) make up 52% of the village, juveniles (> 3 yrs < 18 yrs) make up 35%, and infants <= 3 years old make up 13%. The genealogical database constructed for all village members shows a male-biased sex ratio at birth for residents and their close relatives (127/100, *n* = 602 births), which becomes less pronounced by age 20 (118/100) and is close to parity by age 53, due to excess male mortality through the lifespan. However, female-biased outmigration, along with immigration of distantly-related males, led to males comprising 54% of person days monitored during our study period.

Linao has no running water, no electricity, and no structures for human waste disposal. (Residents defecate directly into the ocean, from discretely located holes in the village pier structure). Drinking water comes from a neighbor’s well-pump ~2 km from the village, and washing water from small open wells that belong to neighboring Blaan tribal people. Approximately 50-70% of children attended school on any weekday, but virtually all village children had dropped out of school by age 10-14 (5^th^ to 8^th^ grade). The nearest small rural health clinic is ~10 km away, and the nearest hospital is ~30 km away, but these are not considered affordable unless a life-or-death emergency arises. (Our research team paid all emergency medical bills for village residents during the study period.) The life history parameters of Linao residents and their close kin are typical of semi-acculturated small-scale societies. In the past half century, 85% of children born (*n*=602) survived to adulthood (18 yrs) and about 63% survived to age 60. Causes of death among children under 18 years (*n*=89 deaths) are infectious/parasitic (55%), congenital (21%), accidents (8%), homicide (4%), childbirth (2%) and unknown (9%). (Note that percentages for causes of death in childhood do not add up to 100% due to rounding.) Adult deaths (*n*=193 deaths) were due to infectious/parasitic (26%), congenital/senescence (35%), accidents (14%), homicide (6%), childbirth (4%) and unknown (15%).

Female mean age at menarche was 14.0 years (*n*= 43), mean age at first marriage for women was 18.1 (*n*=26) and for men was 23.4 years (*n*=8). A small minority of the first marriages for older women had been arranged (in the distant past), and all marriages observed or reported in genealogical interviews were monogamous. Sama marriage is traditionally quite stable, with only 2 women over age 30 reporting more than one husband in a lifetime (*n*=25). Birth usually took place at home, although women in the study community began to make use of birthing clinics after one resident woman died in childbirth during our study period (2017).

Evidence of a fertility transition is apparent in our interview data, even though we discovered no women in the community using any type of contraception (or even having much knowledge of contraceptive methods). Completed fertility for the most recent cohort of women (under age 45) plateaus at about 4 live births, but the plateau for women over age 60 is about 7 live births per woman (Figure A). We are not sure how the recent lower fertility levels are being achieved, and we observed considerable heterogeneity, with one 34-year-old woman already pregnant with her 9^th^ child in 2019, whereas 1 married woman in her mid-40s and one in her 50s had experienced only 1 pregnancy in their lifetimes (and reported no contraceptive use).


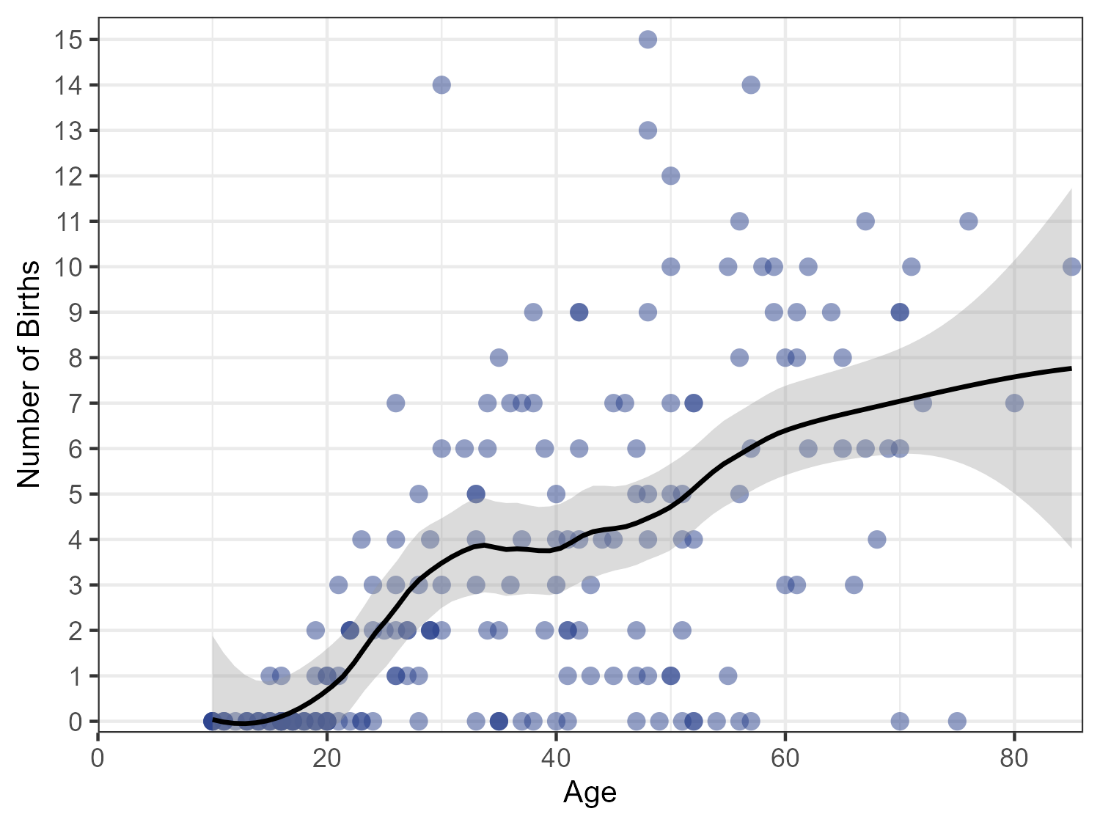


**Figure A.** *Cumulative live births by age of woman. Sample includes all women in the Linao community, as well as all sisters, daughters, mothers, aunts, and grandmothers of adults living in the Linao community.*

**Food production and income**

Interviews show that approximately 3086 kg of fish were captured by Linao men on sample days from June 2016 through November 2018. Reported mean market value of those fish was $1.01/kg, although much of the fish harvested was consumed. Economically most important fish species included mackerels, sardines, carangids, snappers, and a variety of reef fishes (in that order by weight contribution). Intertidal marine foraging was also a common economic activity, that included children as well as men and women. The majority of intertidal foraging took place on the day of spring low tide, and the three days following the spring tides of each lunar cycle. (Figure B). Mean caloric acquisition rates of adult foragers were approximately 300 Kcal per hour of collecting (Figure C).


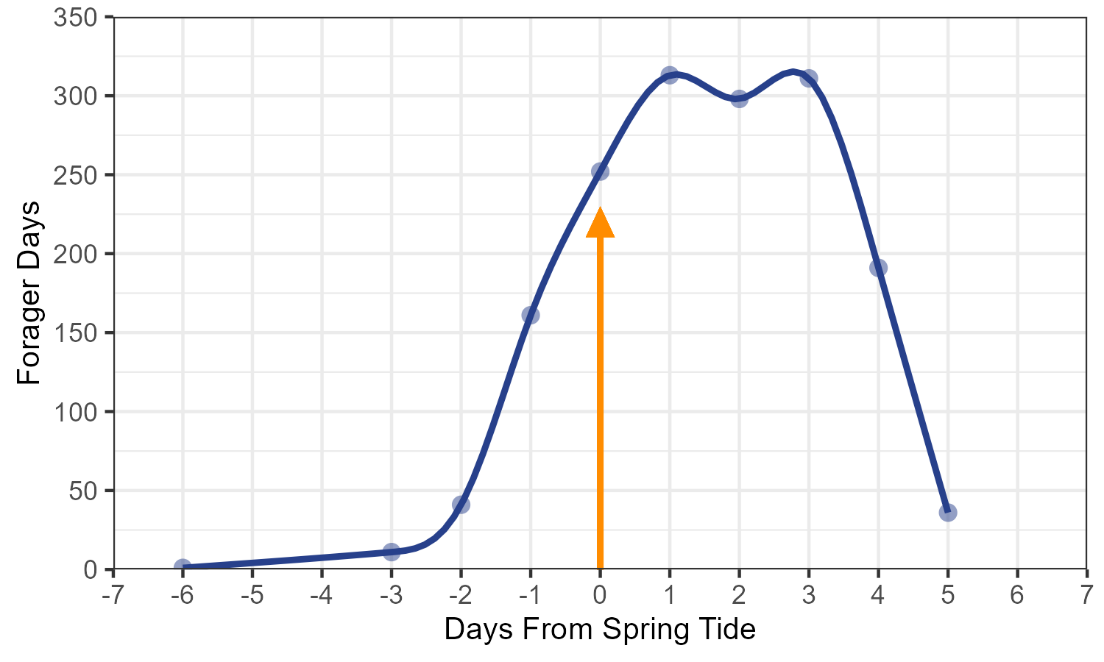


**Figure B.** *Number of observed foraging days (blue dots) by tidal cycle (days before and after spring tide) for Linao foragers. Blue line is calculated as a B-spline with 9 knots in order to demonstrate the general pattern of the data, and orange arrow indicates spring tide. Lowest tides in Sarangani usually occur on the day following spring tide (+1).*


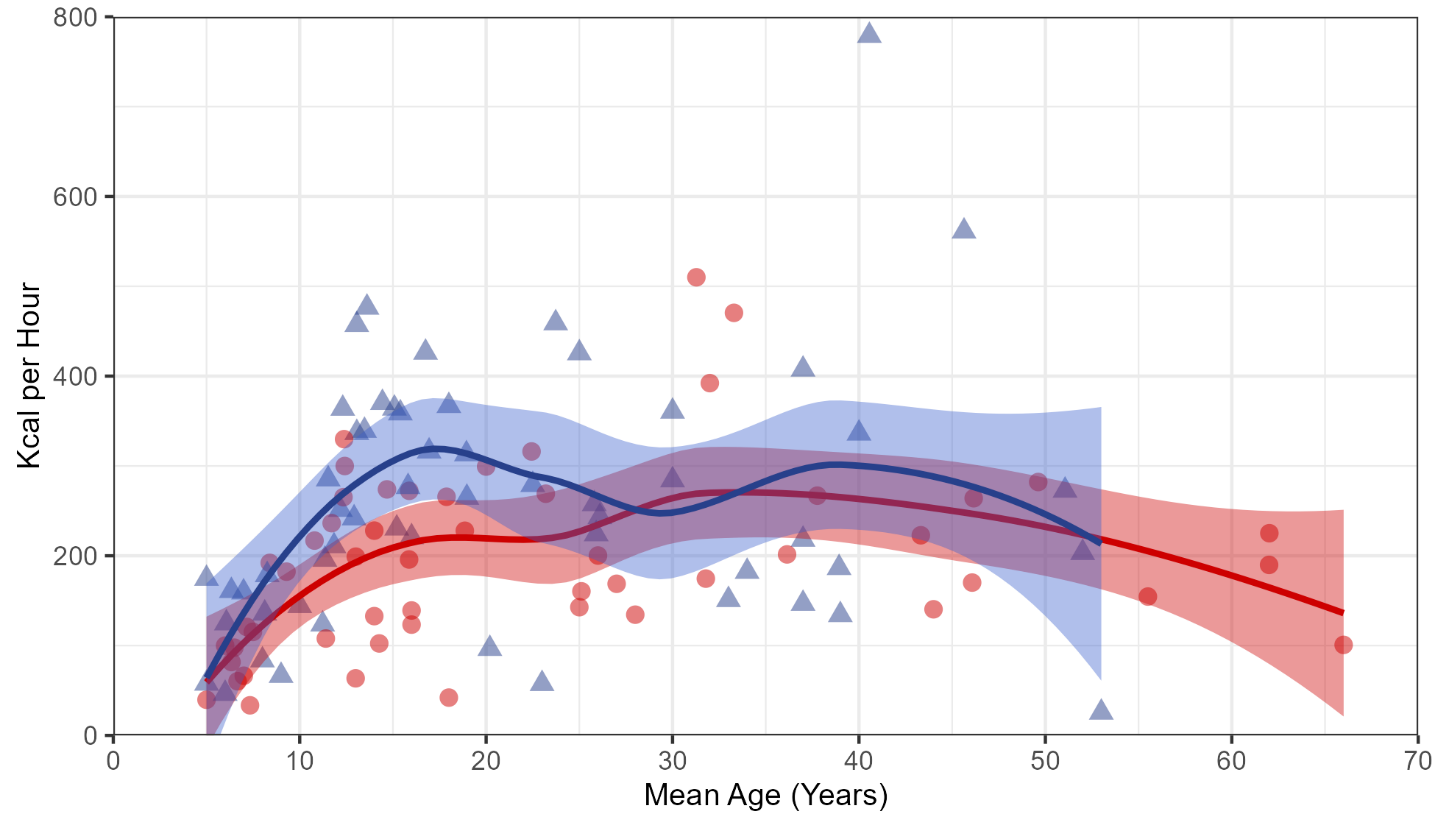


**Figure C.** *LOESS smooth of mean caloric return rate from intertidal collection of marine invertebrates by 53 females (red circles) and 56 males (blue triangles). Approximate caloric values of foods were estimated by assigning the values from similar resources in other published studies.*

Total HH income from all sources was elicited via 24-hour recall, for a sample of 493 HH production days (2232 person days) from June 2015 through November 2018 (Table 1, main text). Income is derived mainly from fishing (37%) *resale* (24%) and collecting (19% - firewood, water, plant foods, recyclable plastic). The highest recorded single day incomes were greater than $70 per day (4 sample days), and the lowest daily incomes were $0 (15 sample days). Mean daily HH income over the whole study period for 36 HHs was $6.97 per day, and ranged from about $25 per day for the highest mean HH income, to around $1 per day for the lowest mean HH income (both female headed HHs, Figure D). When family size is taken into account, mean daily *per capita* HH income was $1.67 per capita/day and ranged from about $7 per capita/day to around $0.50 per capita/day. The mean per capita HH income is therefore only about 1/5 of the Philippine National mean per capita daily income (2017) of $7.92 per person per day [3].


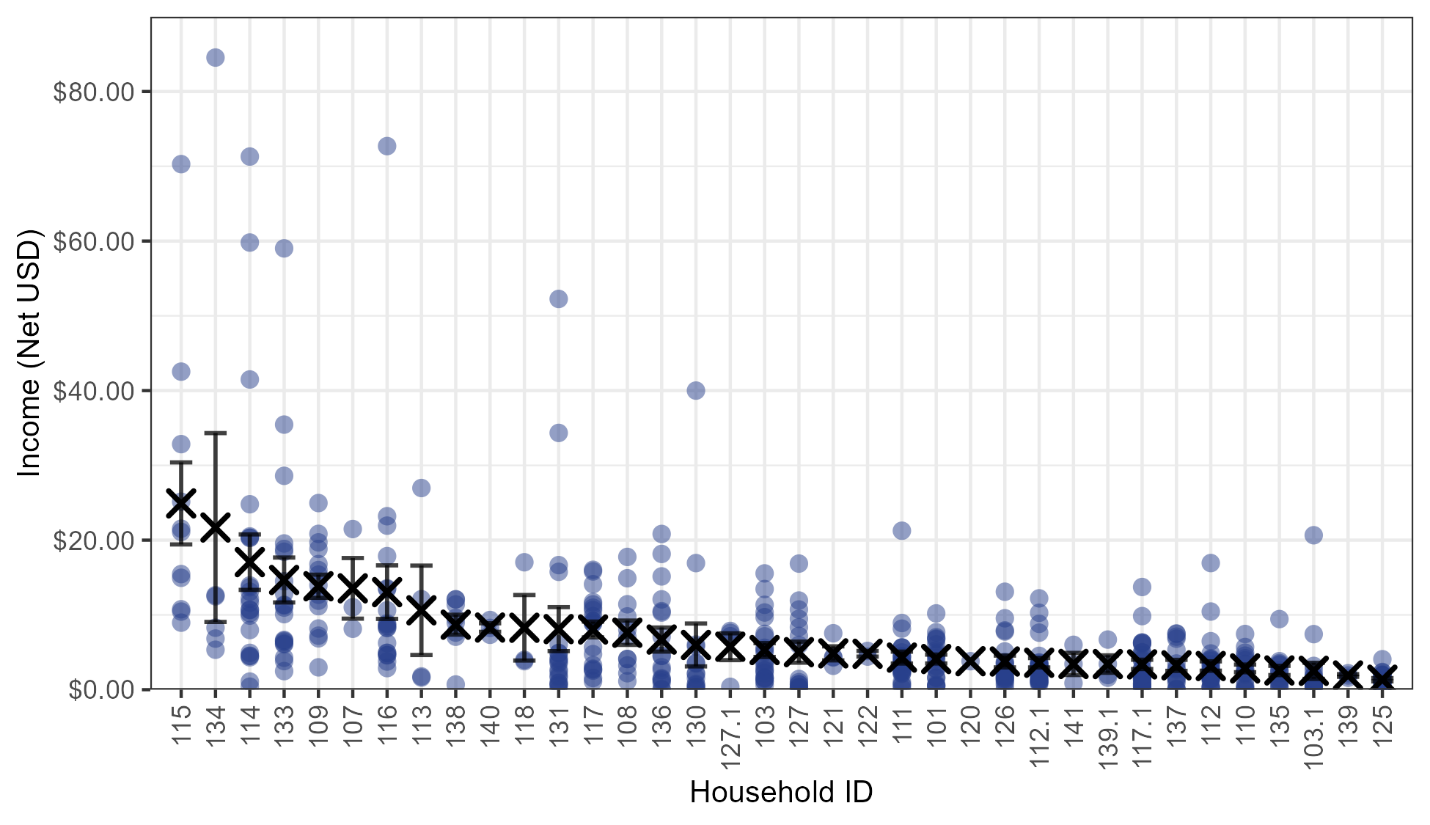


**Figure D.** *Mean daily net HH income for 36 HH in Linao. Xs are HH means, while dots are daily HH observations. Error bars represent +/- one standard error about the mean for all HHs with >1 daily observation.*

Daily variation in HH income was quite high, with a mean coefficient of variation for the 36 HH of 0.88. Importantly, 28% of the HH days sampled showed a per capita daily income of less than ½ the value of mean daily per capita food intake expenditure. This implies that food needs were probably not met by daily HH income on at least 28% days. No HH monitored had any significant cash savings to buffer this income variability, hence food sharing was probably critical to nutritional well-being and health for most HHs.

The degree of income inequality in the Linao settlement can be quantified by calculating the GINI coefficient for mean HH income (GINI = 37.7) and per capita income (GINI = 39) across the 36 HHs. The numbers indicate that intra-village economic inequality is somewhat lower than for Philippines as a nation (2015 GINI = 44.4) In Linao, the daily income of the top 10% of HHs was 10.8 times higher than the daily income of the bottom 10%. Means of production were also unequally distributed, with only 61% of HH owning a boat, 43% owning a motor, and only 30% owning a net. Those without nets, motors, or boats, worked in cooperative teams with “owner” HH, but received a lesser share of the fish harvest under *Palaran* sharing rules. In short, Linao HHs experience both frequent asynchronous *need* due to daily income variance and long-term inter-HH income inequality that implies greater need by those HHs with lower mean per capita productivity.

We further divided the HH sample into five categories (Figure E) for some analyses presented later. These are: 1) *rich*, > ½ s.d. above mean total HH income AND > ½ s.d. above mean per capita daily HH income; 2) *high producing*, > ½ s.d. above mean total HH income BUT < ½ s.d. above mean per capita daily HH income; 3) *low producing*, > ½ s.d. below mean total HH income BUT < ½ s.d. below mean per capita daily HH income; 4) *poor* > ½ s.d. below mean total HH income AND > ½ s.d. below mean per capita daily HH income. All the remaining families were considered *middle* *income*, and neither poor nor rich. These distinctions matter, because *rich* HHs experience lowest marginal fitness costs when they transfer resource to others and *poor* HHs achieve highest marginal fitness gains when they receive material goods.


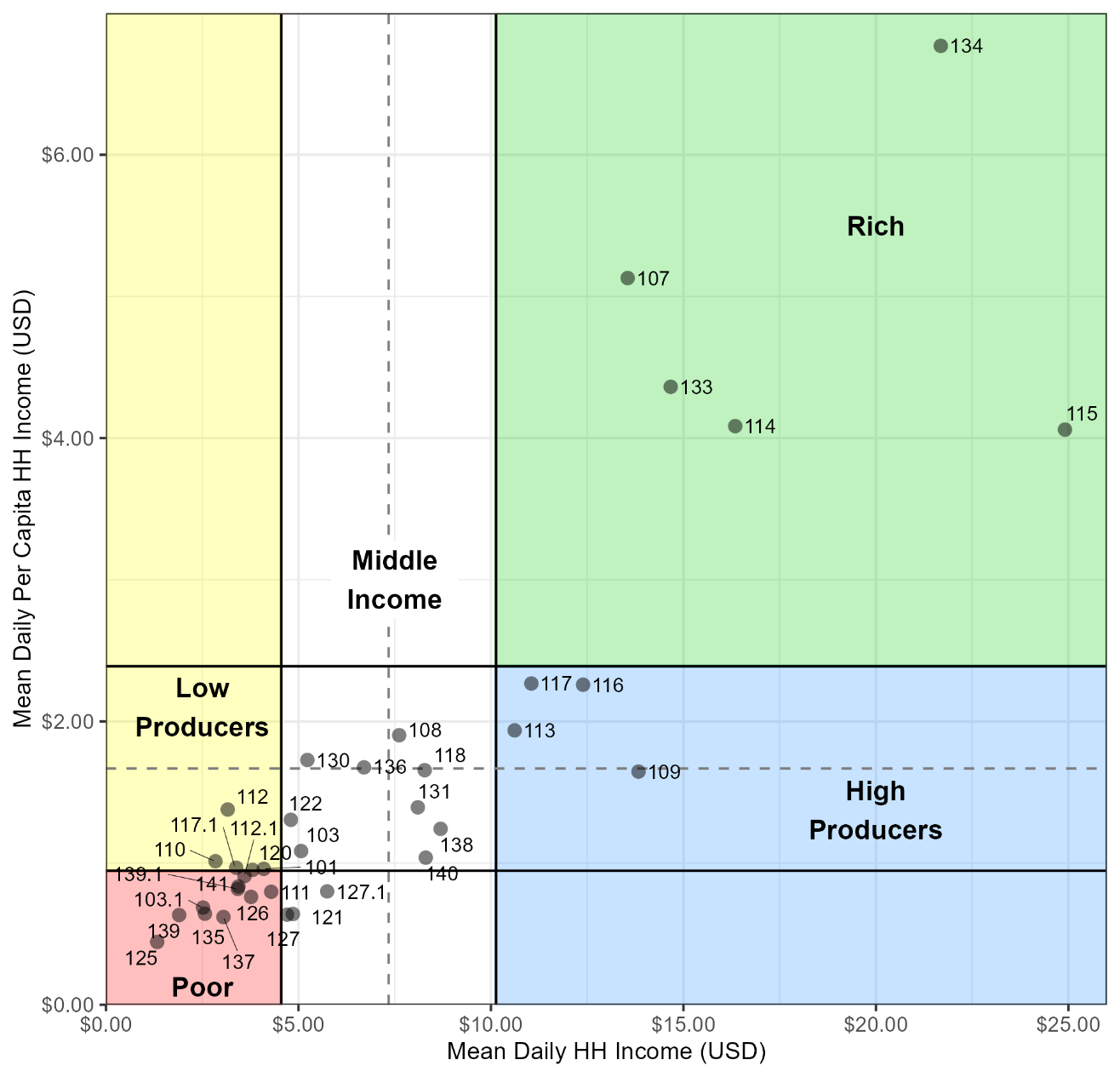


**Figure E.** *The relationship between mean daily HH income and mean daily per capita HH income for 36 HH in Linao. Dashed lines indicate mean income, and solid lines indicate mean income +/- half standard deviation.*

While age-sex distribution of individual income from fishing, intertidal foraging, and total income all suggest that we might expect resource flows from older (high producing) individuals to younger (lower producing) individuals, HH income differences are a function of both the age-sex *structure of individual production* AND the age-sex *composition* of HH members. Because income peaks in middle age, but number of dependent children also peaks in middle age, it is not simple to predict how per capita HH income (family *need*) will change across the lifespan of married couples. Figure F shows the relationship between mean age of HH head (husband and wife for most HHs) and daily total productive income. In general, it appears that HHs of young couples and very old couples have lowest mean daily total income in the village, although the 95% confidence intervals almost overlap across the entire age spectrum of HH heads.


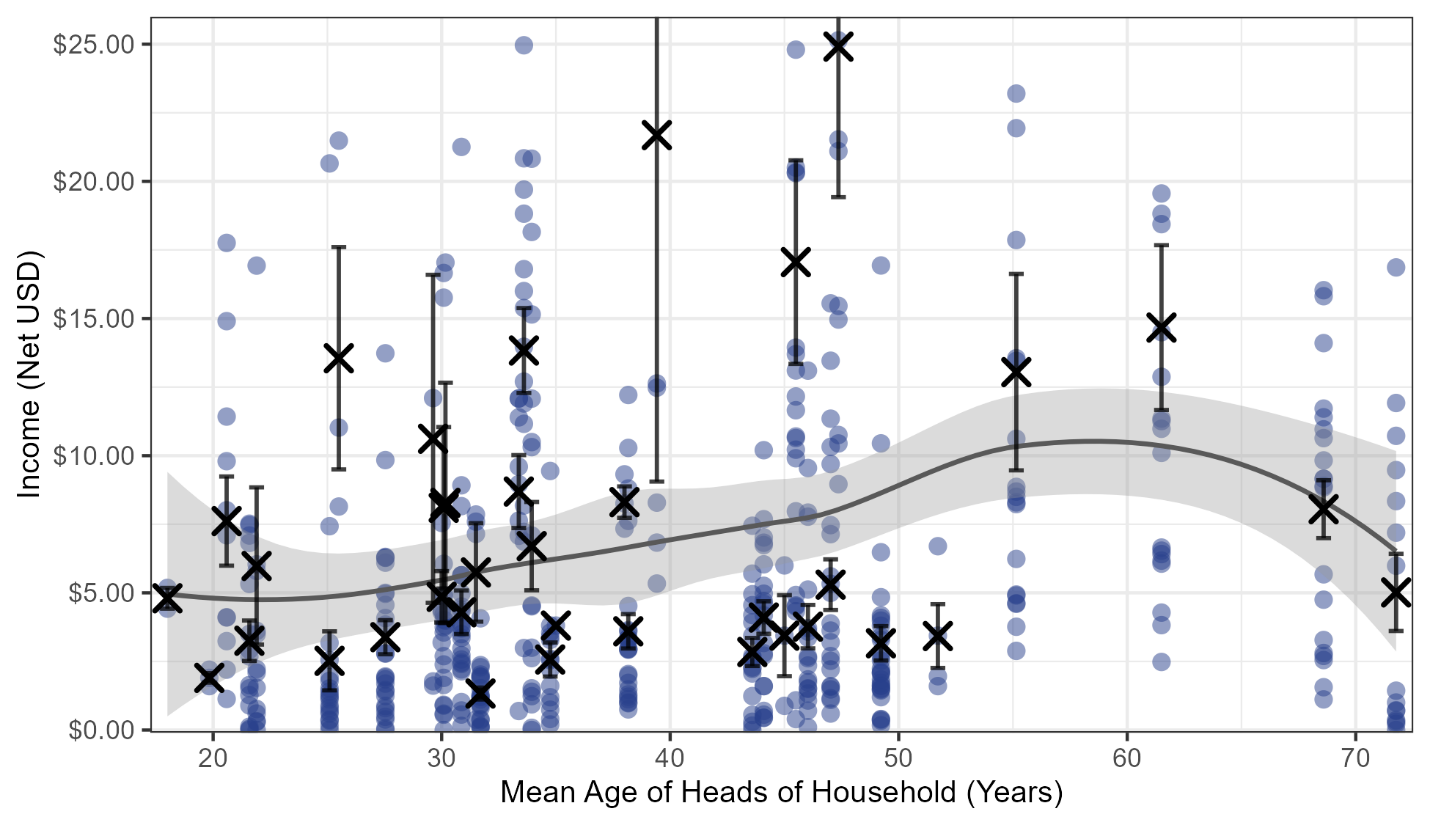


**Figure F.** *HH mean daily income as a function of mean age of heads of HH for 36 HHs in Linao. Xs are HH means, while dots are daily HH observations. Error bars represent +/- one standard error about the mean for all HHs with >1 daily observation, and the line shows a LOESS smooth of the data points. Large daily outliers are not shown, but are included in calculations.*

**An Alternative Income Measure**

While detailed inflow interviews provide a complete measure of ALL types of income for specific HHs on particular interview days (n= 493 HH days), we also performed a “fishing monitoring” interview to record marine resource production for every HH on every day that we visited the Linao community. This interview records all fish acquired by each HH in the community on every day of fieldwork, and consists of about 15,400 person days of resource acquisition during the study period. Because fish make up about 37% of total HH income during the study period (see above), we can use this database to estimate HH income for every HH on every day in our statistical models. This has the advantage that it provides a much larger sample of daily income for each HH, but has the disadvantage that it does not capture all sources of income that were important to some families (especially monetary income). The correlation between daily (Pearson’s *r* = 0.36), weekly (Pearson’s *r* = 0.33, not shown), or study period (Pearson’s *r* = 0.30) income from the two sources suggests a fairly good correspondence (Figure G), but also enough difference to allow us to use these as partially independent measures of income in our models.


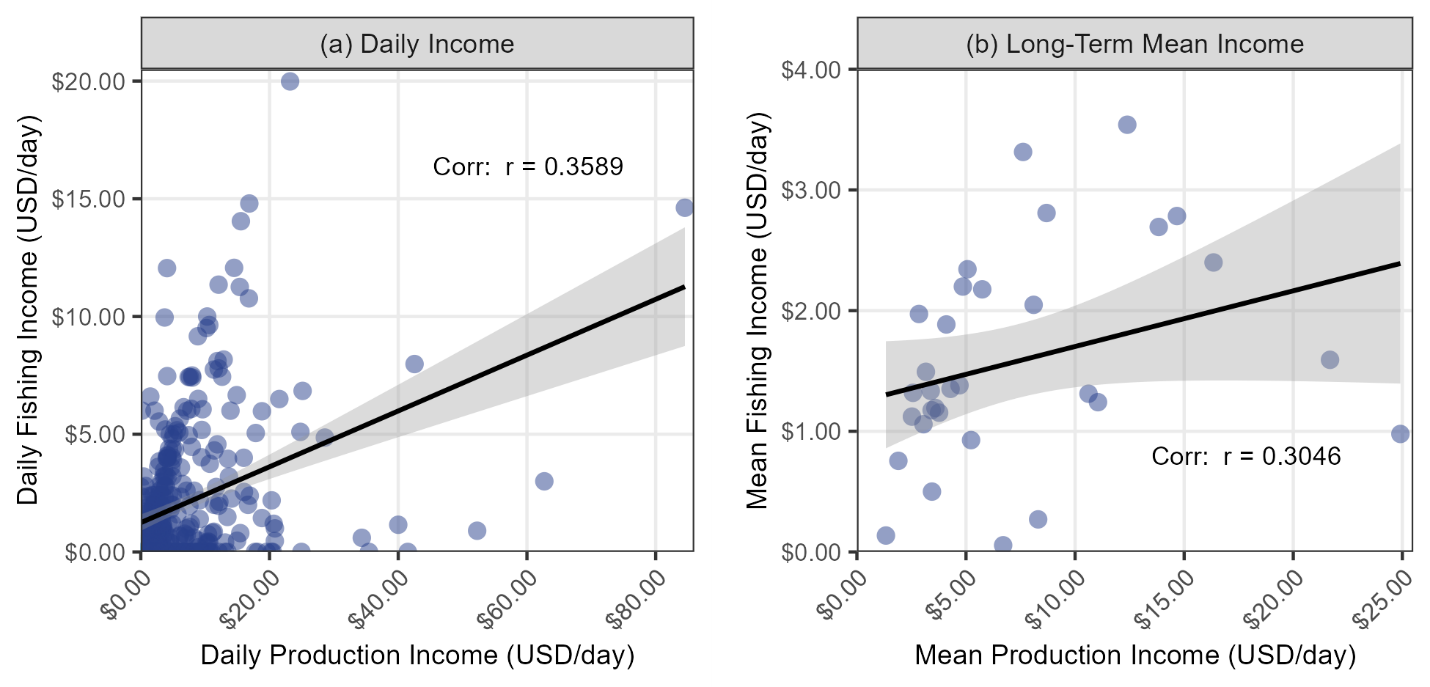


**Figure G.**  *Daily (a) and long-term (b) correspondence between total HH “production” income reported and “fishing monitoring” income reported in independent interviews.*

**Which households give more or receive more?**

The first issue we examined is the relationship between giving and receiving for all HH over the three-year study period. Explanations of resource transfers that assume transfers are HH *assistance* led us to expect that high donor and high recipient HHs should have different characteristics. However, preliminary analyses showed instead that the HHs which reported higher mean daily *outflow* over our study period were the same HH that reported higher mean daily *inflow* over the study period (Figure H). This immediately suggests that *reciprocity* and trade might underly more of the transfer that we observed than does the scenario of helping of needy families.

**
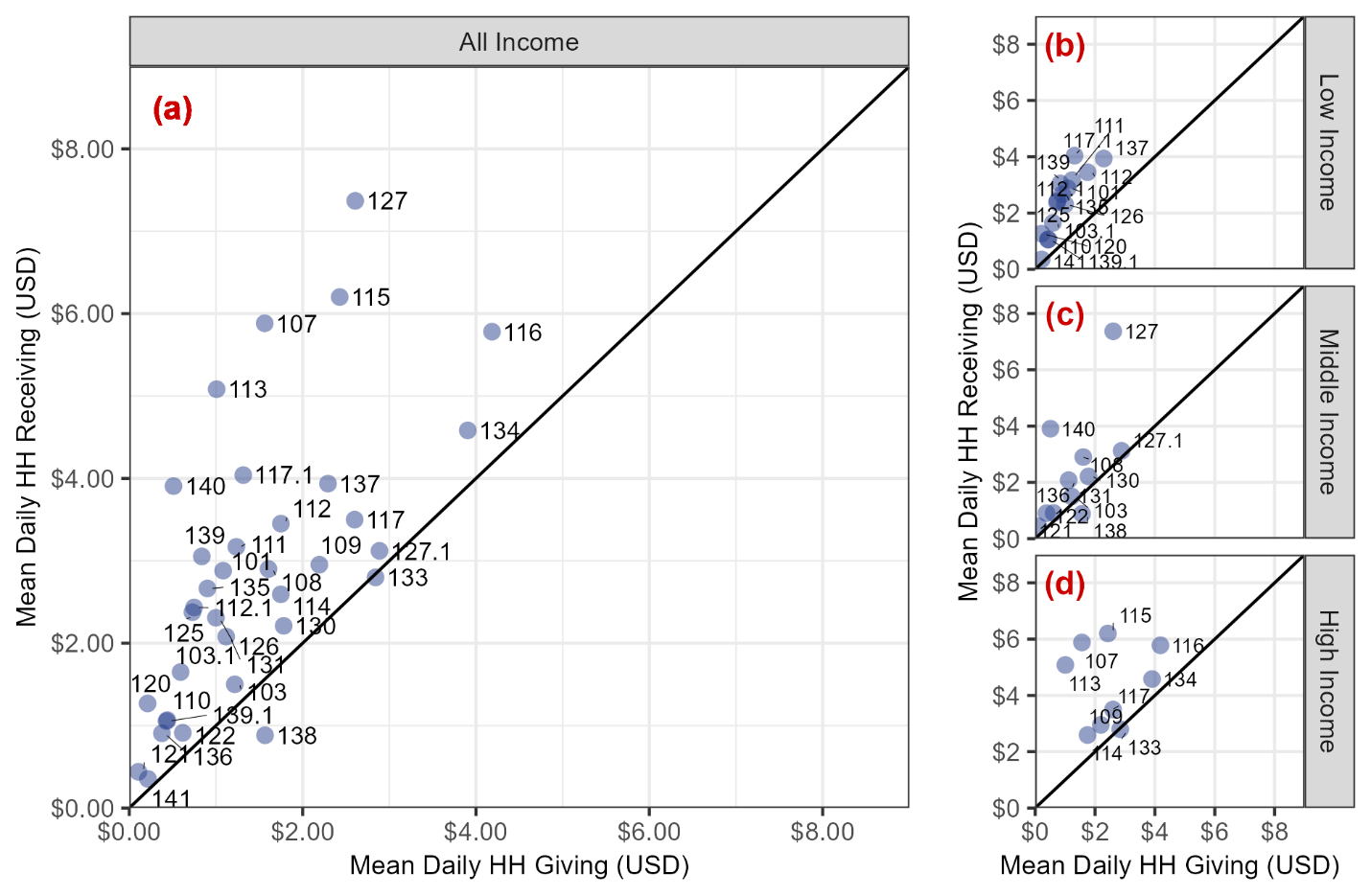
**

**Figure H.** *The relationship between mean daily amount given (outflow), and received (inflow), for 35 out of 36 HHs during the entire study period (a), and the trend within income levels low (b), medium (c), and high (d) daily HH income. The line constitutes perfect correspondence between giving and receipt (slope = 1). Numbers indicate different HH in the study. One outlier HH has been omitted because they were only observed 3 times and made a large one-time gift to another HH.*

Note that high-income families do not cluster in the bottom right corner, nor do low-income families cluster in the top left corner, as would be expected if resources tended to mainly flow from high-income to low-income HHs. Instead, the trend shows positively correlated giving and receiving within and across income levels. The biggest outlier on these graphs is HH 127 – the village “captain” – whose HH received much more than they gave.

**Models of HH giving (outflow)**

In-depth interviews, usually with the female head of a randomly-selected HH, were conducted using a 24-hour recall on each day of fieldwork (493 interviews over 41 months) to solicit information on all transferred *inflows* and *outflows* of material goods to/from every individual in each HH sampled. Because the Linao settlement contained 36 different HHs over the study period, we must be concerned about whether enough interviews were done in order to adequately characterize different HHs as high *givers* or *recipients* of material goods. The sample for our models includes 455 HH interview days across 32 HHs, all of which were interviewed at least 3 times and had complete data for both income indicators. However, a frequency distribution of total interviews conducted on each HH shows that about 1/3 of the HHs were interviewed on less than 10 days over the 1248 day study period (Figure I). Likewise, about 1/3 of all HHs were interviewed 10-20 times, and a similar number were interviewed more than 20 times. Because of this, we decided to repeat analyses using only more frequently interviewed HHs to test for the robustness of reported results; these robusticity checks (not shown) largely agree with the 32-HH analyses presented in the main paper and this supplement. In particular, the estimated effects of all explanatory variables are consistent across the models using the full and reduced samples, and differences in statistical significance between models using the reduced and full samples appear to be driven primarily by lower sample sizes in the reduced models. Hence, observed statistical patterns in the full models are not being influenced by less-sampled HHs, but appear to be consistent across all sampled HHs.


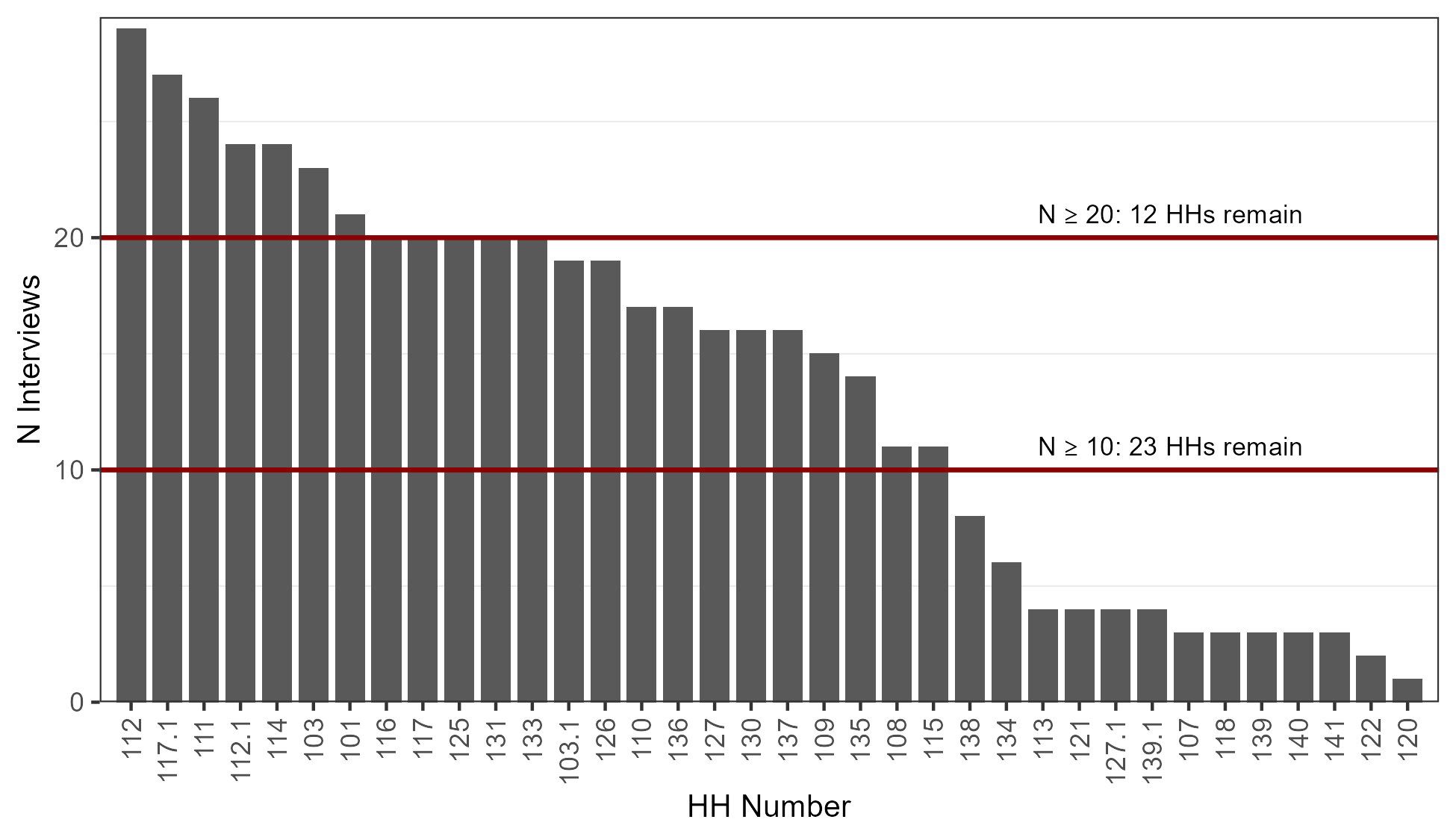


**Figure I.** *Sample size of days interviewed for each HH in Linao, between June 10, 2015 and November 9, 2018.*

Because we suspected non-linear relationships between age, or income and daily giving patterns, we first plotted daily amount given by mean head of HH age or daily production income and performed a LOESS smooth on the array of 455 data points. Plots are show below (Figure J) and confirm that these two variables have non-linear associations with daily giving.

**
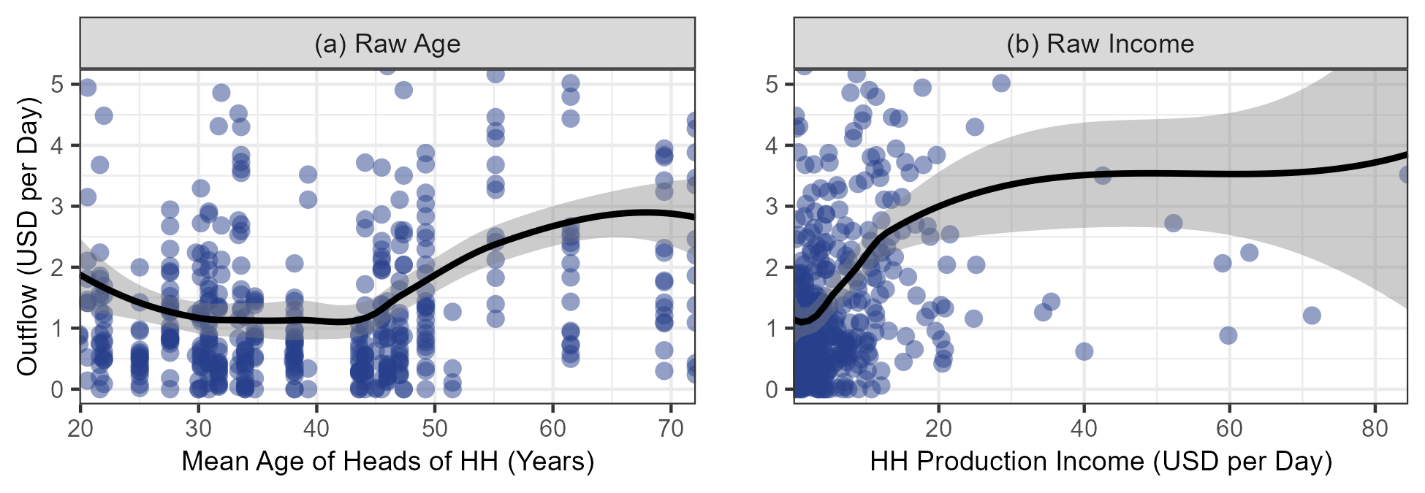
**

**Figure J.** *LOESS smooth (and 95% CI) of (a) age and (b) income by total HH outflow for all interviews in the sample. The smooths suggest that giving is lowest in middle age, and that the income effect on daily giving diminishes at very high daily incomes. Hence both variables were modeled as non-linear effects (see text).*

Our first model examined daily giving as a function of five long term variables: 1) income; 2) income variation; 3) juvenile consumers; 4) age of HH heads; 5) residential permanence. Results show that HHs with higher mean income (average of fishing monitoring income over previous 6 months), younger or older HHs, and HHs with greater residential permanence give more (Table A). Number of juvenile dependents showed a marginal but not significant effect, and fishing income variation showed no significant association with HH giving. This basic model of giving accounts for about 23% of the variance in daily giving (adjusted *R^2^* = 0.23).

**Table A.** GLM of total daily amount given (outflow) by HH characteristics.


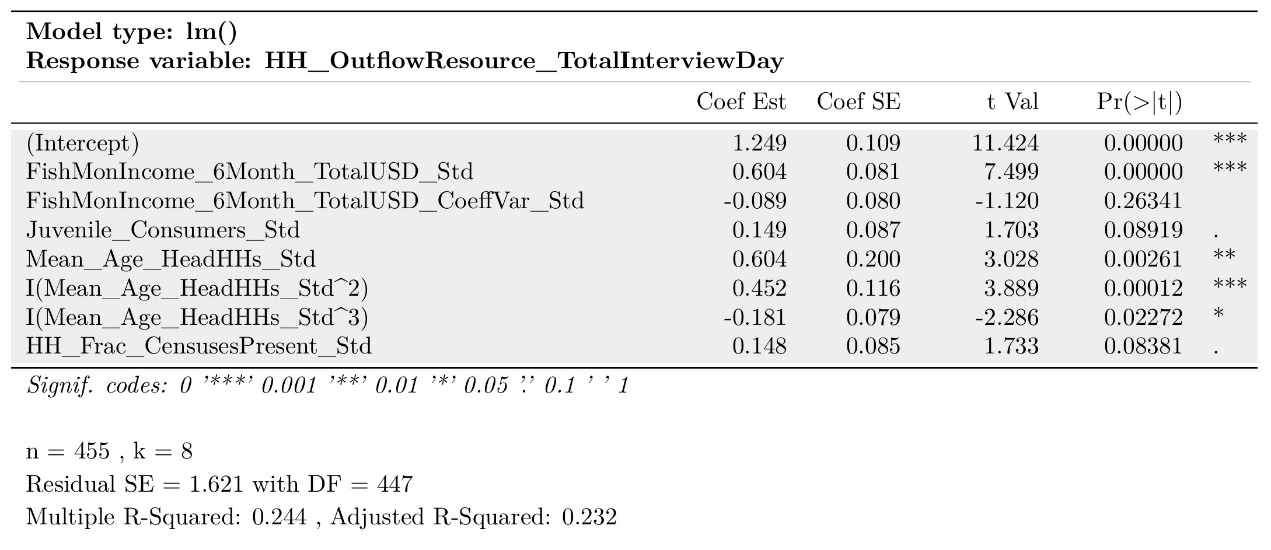


Because single data points in this model represent the giving on only one specific day, we modified the model to try to account for some of the fluctuation (“noise”) observed in the day-to-day giving patterns. Specifically, we examined whether adding daily income (production on the day of the interview), in addition to our long-term fishing income measure, might account for more of the day-to-day fluctuation in reported HH giving. We also examined whether high “receipt” of material goods from other HHs on the day of the interview is associated with higher daily giving. Results in the main text (Table 2) indicate that the new model accounts for 35% of the observed variance in daily HH giving (Table 2, adjusted *R^2^* = 0.35). Plots also show that the age and daily income effects are indeed significantly nonlinear (Figure K).


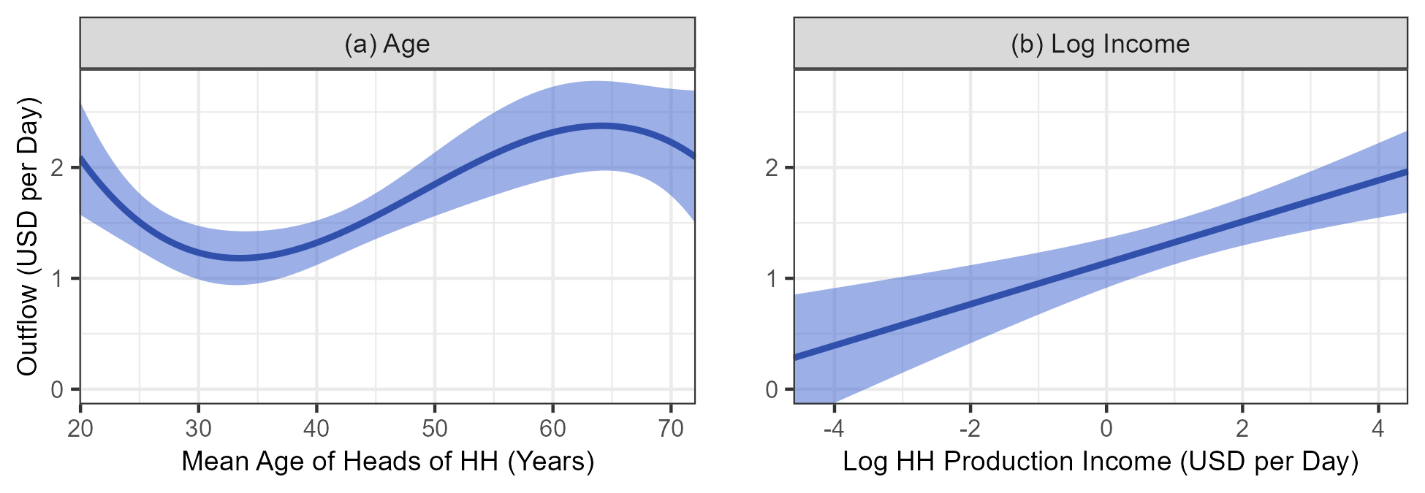


**Figure K.** *The shape (95% CI) of (a) non-linear effect of mean age of HH heads and (b) linear effect of the natural log of production income on daily HH outflows. Independent variables were converted back from standardized to raw variable values, and all other variables were set to the sample mean value. Note good correspondence between model fit and the shape determined by the age LOESS smooth in Figure J.*

In order to further control for the daily variation in giving, we also examined whether there were specific HHs that simply tended to give more than others even with all other variables controlled. This led us to develop a Generalized Linear Mixed-Effects Regression (GLMER) model with random effect variables for each of the 32 HHs in the sample. Table B(a) shows that this model explains only slightly more of the total variation in daily giving than the previous model (Table B(a), conditional *R^2^* = 0.38 vs. Table 2, adjusted *R^2^* = 0.35). Additionally, a large-sample likelihood-ratio test of the random effect of HH number yielded a non-significant *p*-value under the $\chi_{1}^{2}$-$\chi_{0}^{2}$ mixture distribution (Λ=1.006, *df* = 1, *p* = 0.16). Simply knowing which specific HH is the potential donor on a sample day (with other relevant variables controlled) does little to improve the model fit once the already included variables were controlled. As the random effect assumption is not altogether reasonable for a sample from a population of this size, we also tested a GLM with a *fixed* effect for each of the 32 HHs (Table B(b)); however, we found that the fixed effect for HH covaried significantly with the model’s long-term HH characteristics (i.e. 6-month mean fishing income, mean age of heads of HH, and fraction of censuses that HH was present), leading to inestimable levels of the fixed effect when also including the long-term variables. This suggests that HH-level variance is captured just as well by the long-term household effects as it is by the fixed effect for household. Given that HH number is not biologically meaningful, we therefore chose to omit the fixed effect for HH and retain the long-term HH characteristic variables in all subsequent models.

**Table B(a).** GLMER of total daily amount given (outflow) by HH characteristics.


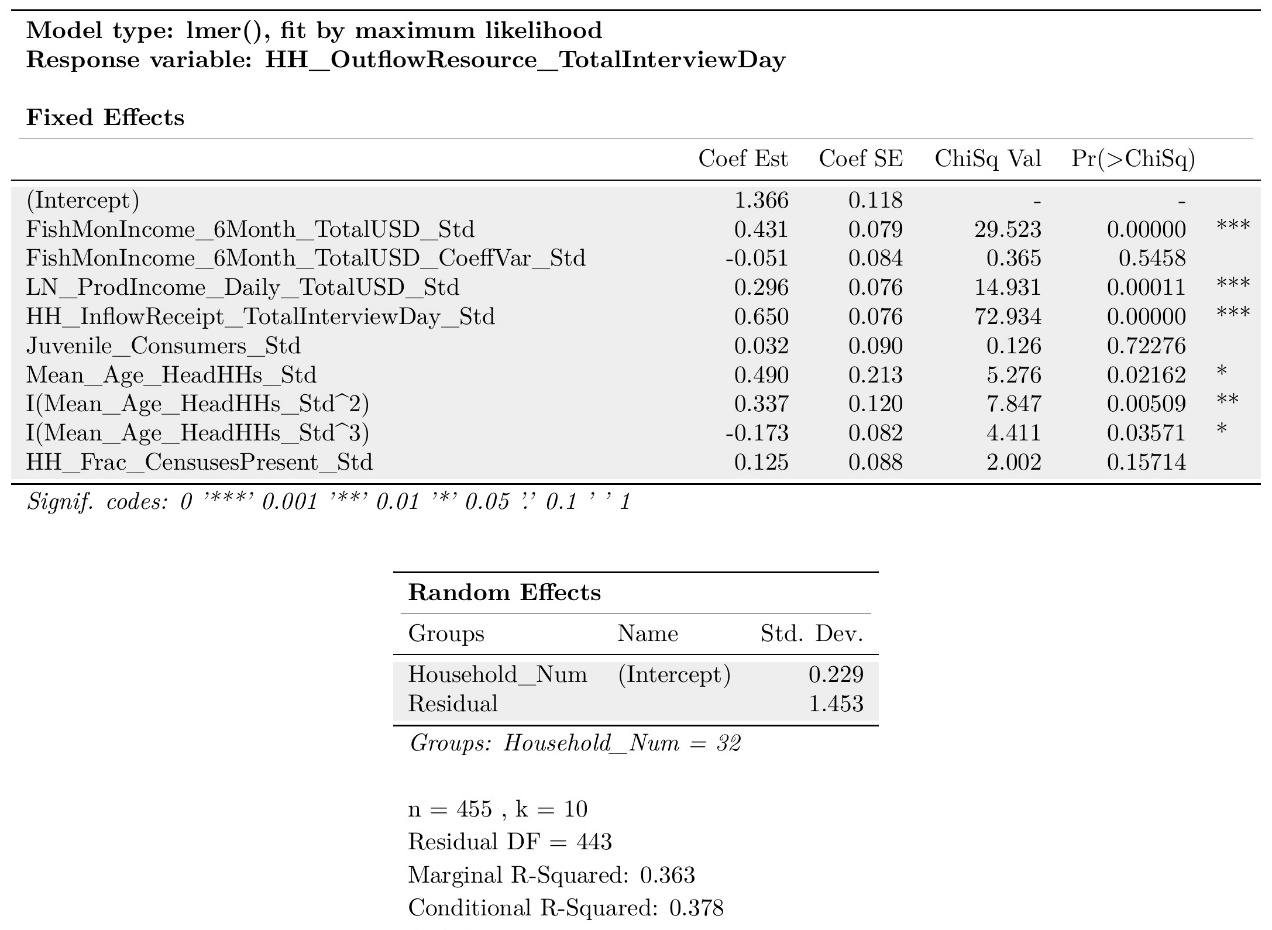


**Table B(b).** GLM of total daily amount given (outflow) by HH characteristics, including fixed effect for HH.


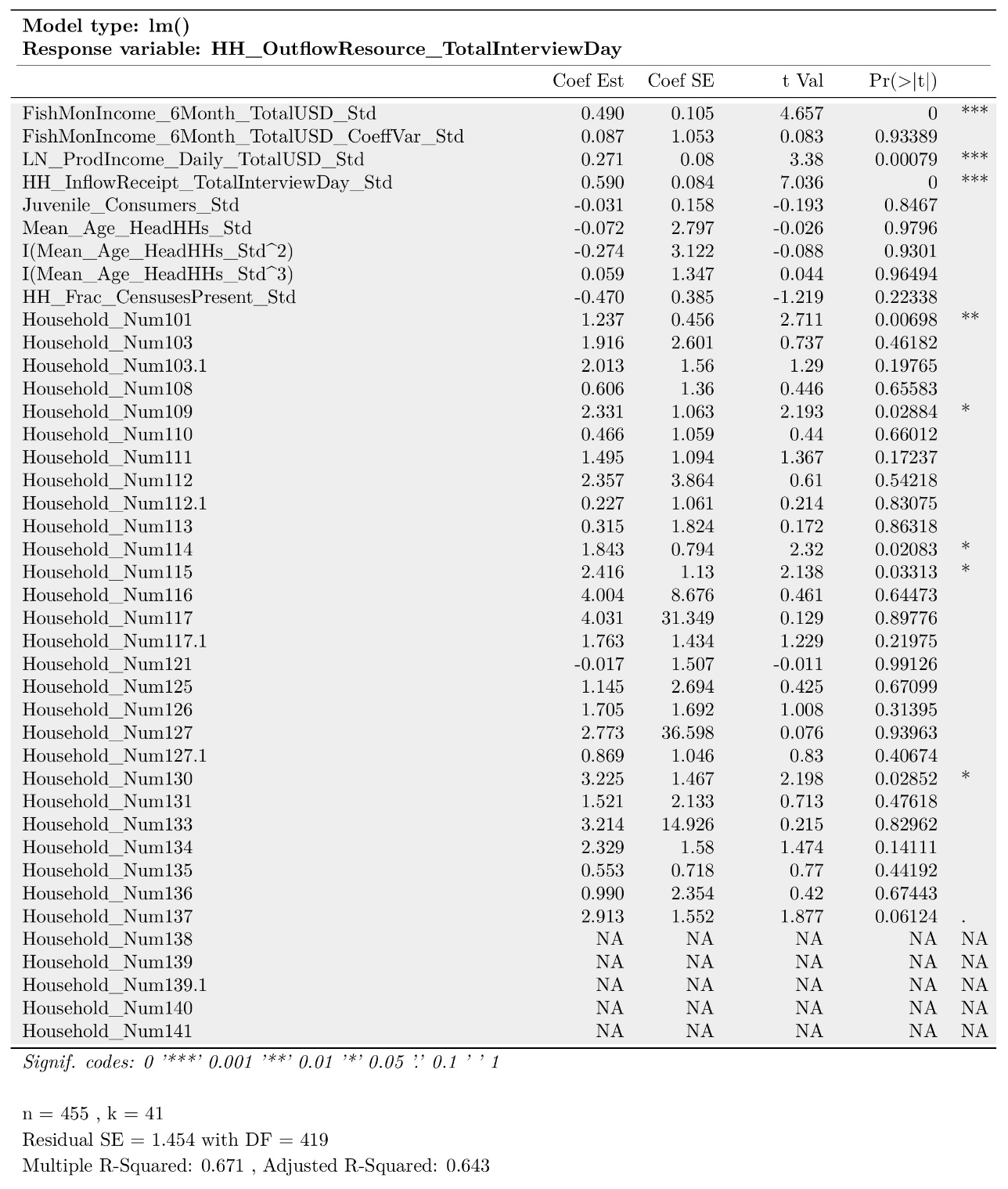


After discovering that the GLM of total outflow over the entire sample period explains more variance in HH giving but with only 2 significant independent variables (Table 2 vs. Table 3, main text), we also examined the long-term outflow of *food only* (Table C), *money only* (Table D), and *other goods* only (Table E). When we examine food outflow over the entire sample period, the long-term model explains more variance than the daily outflows model (Table C vs. Table 4, adjusted *R^2^* = 0.85 vs. 0.39), but fishing income is no longer a significant predictor of mean daily food outflow (Table C).

**Table C.** GLM of HH mean daily outflow of **food** by HH characteristics.


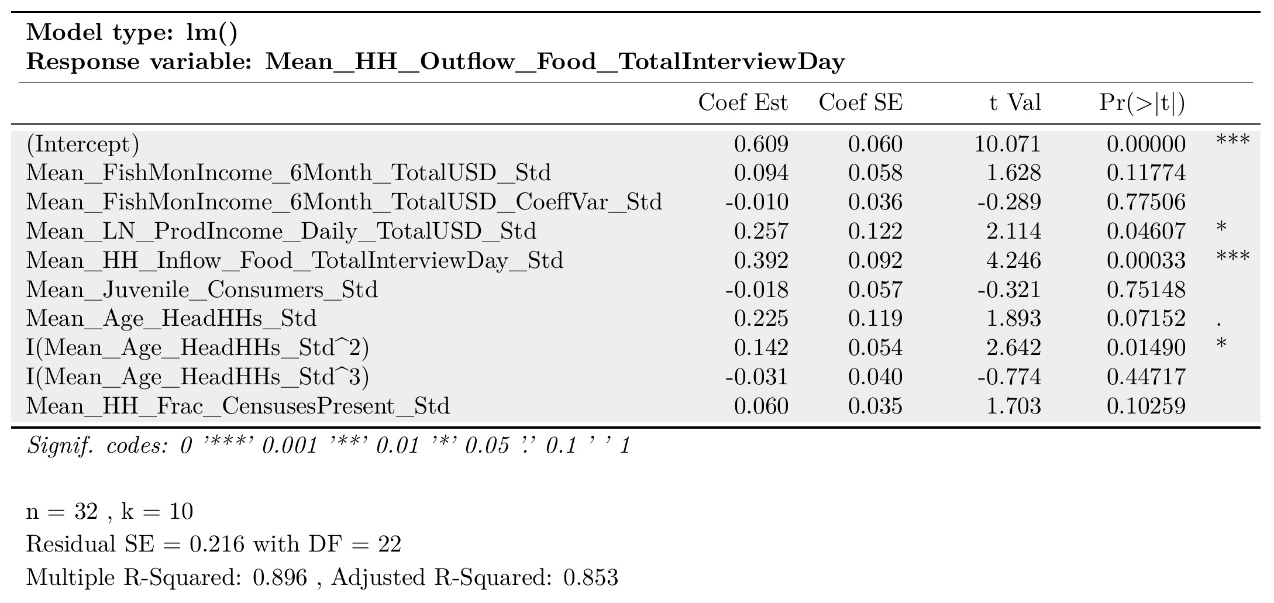


Likewise, the model of HH transfer of *money only* over the entire sample period accounts for considerably more variance than the daily money transfer model in the main text (Table D vs. Table 5, adjusted *R^2^* = 0.53 vs. 0.20), but less variance than does the long term GLM for *food only* or total transfers (Table D vs. Table C vs. Table 3, adjusted *R^2^* = 0.53 vs. 0.85 vs. 0.67).

**Table D.** GLM of HH mean daily outflow of **money** by HH characteristics.


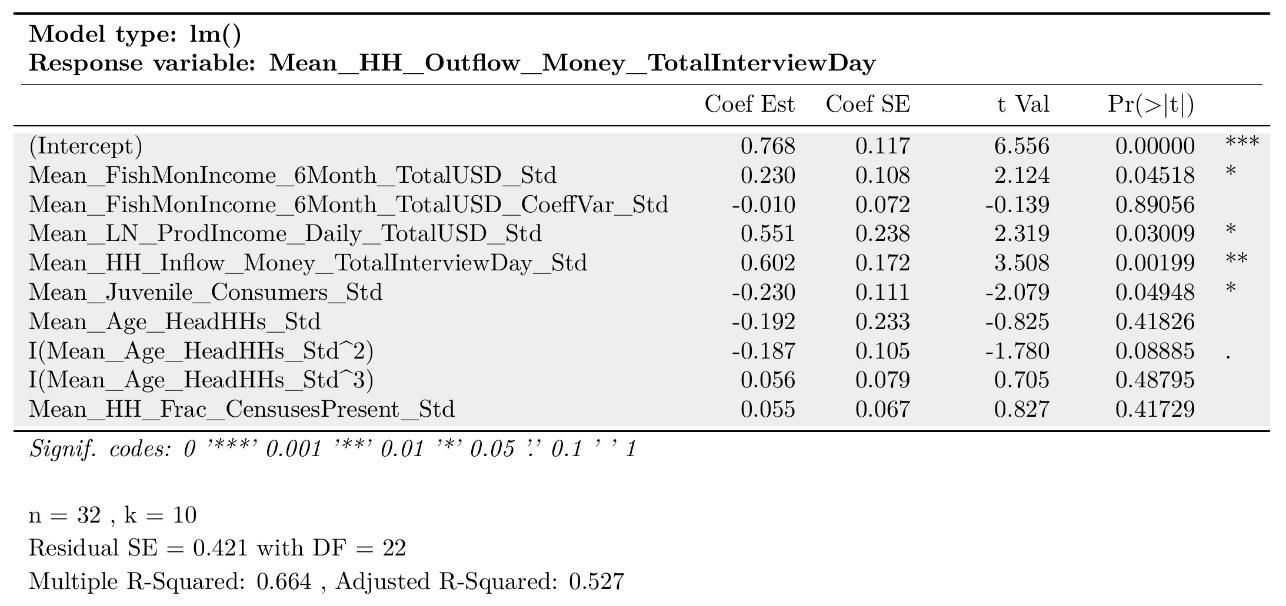


Finally, when we examine transfers of *other* *goods* (clothes, medicine, tools, adornments, etc.), the GLM over the entire sample period accounts for the least variance (only 29%) of any of our long-term models (Table E, adjusted *R^2^* = 0.29). Interestingly, the model suggests that giving of other goods may be negatively associated with mean daily production income over the study period.

**Table E.** GLM of HH mean daily outflow of **other** goods by HH characteristics.


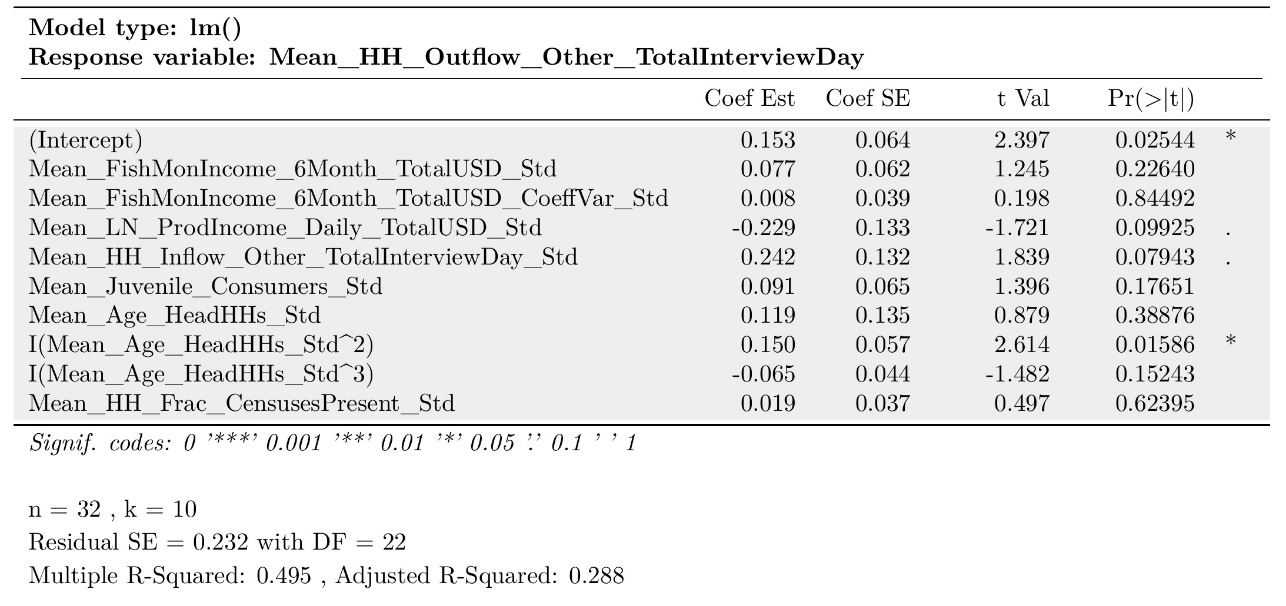


**Models of HH daily receipt (inflow)**

We first examine whether the relationship between age or income and daily receipt of material goods is curvilinear. Value of all received goods on each interview day is plotted by mean age of HH heads on that day, and also by reported production income each day, and both scatterplots are fit with a LOESS smooth. The plots (Figure L) suggest that the age relationship is U-shaped, but the income relationship is perhaps increasing non-linearly (although this is far from certain, given outliers).


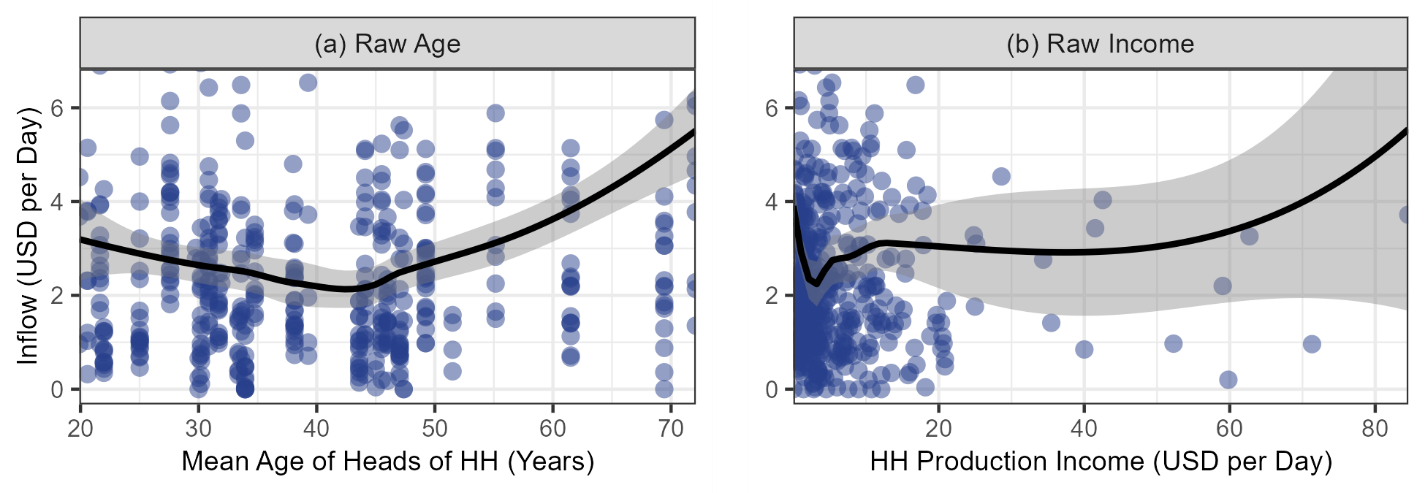


**Figure L.** *LOESS smooth (and 95% CI) of (a) age and (b) income by total HH inflow for all interviews in the sample. The smooths suggest that receipt is lowest in middle age, and that the income effect is positive and approximately linear. Note that plots are cropped to exclude large outliers, but these data points are included in calculation of the LOESS curves.*

Results from the GLM model with only long-term variables suggests that HHs with higher mean income over the previous 6-month period (fishing income provides best fit here) generally receive more daily inflow of all goods (Table F, Figure L). The age effect in this model is approaching significance (*p* = 0.084), showing possibly increasing inflows at older ages. Once again, however, this initial GLM only explains a small amount of the total variance (12%; Table F, adjusted *R^2^* = 0.12) in daily inflows to interviewed HH (455 sample days) because of high daily variation in receipt for any given household during our sample period.

**Table F.** GLM of daily inflow of all goods by HH characteristics.


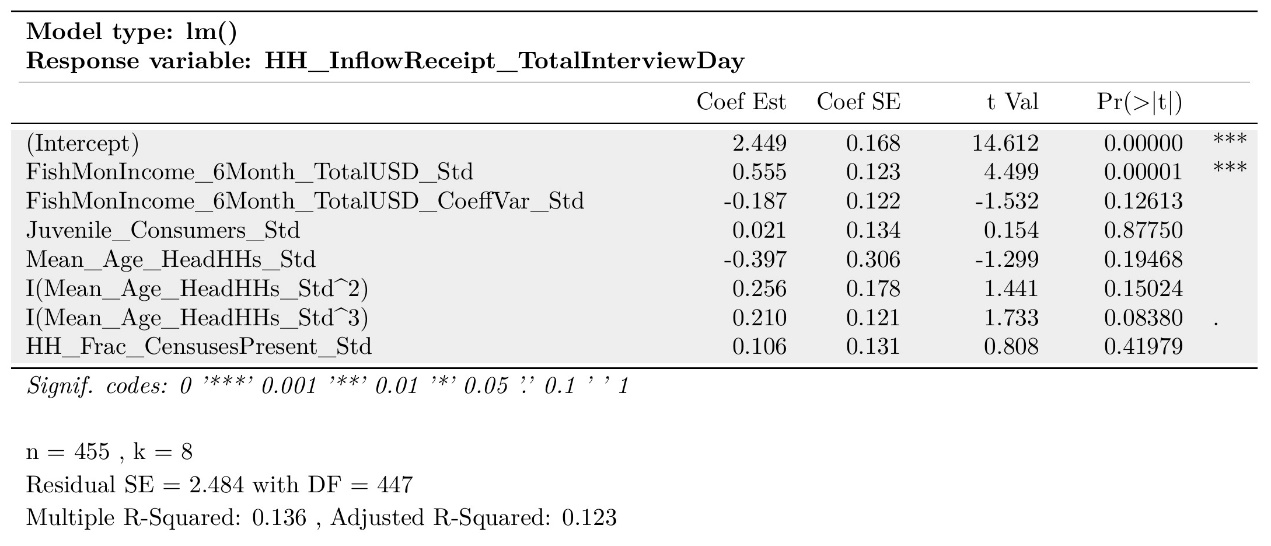


The final GLM model of daily inflows from other HHs shows that long-term income and age are positively associated with daily receipt, whereas income on interview day is negatively associated with daily receipt (Table 7, main text). Amount given on the interview day is also positively associated with amount received on that day (just as we found in the outflows model). Finally, the age effect is complex, with receipt lowest at intermediate ages and higher to younger and older HHs (Figure M).


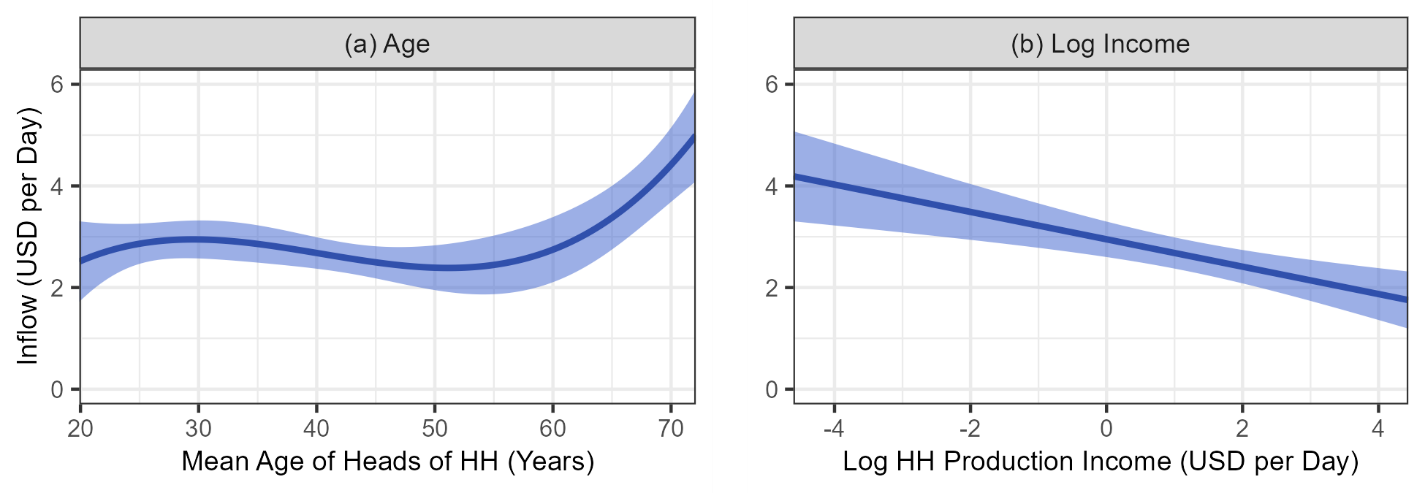


**Figure M.** *The shape (95% CI) of (a) non-linear effect of mean age of HH heads and (b) linear effect of natural log of production income on daily HH inflows. Independent variables were converted back from standardized to raw variable values, and all other variables were set to the sample mean value. Note good correspondence between model fit and the shape of the age LOESS smooth in Figure L.*

In order to further control for the daily variation in receipt, we also examined whether there were specific HH that tended to receive more than others, even with all other variables controlled. This again led us to develop a GLMER model with random effect dummy variables for each of the 32 HHs in the sample. Table G shows that this model explains more of the total variation in daily giving (35% vs. 26%) than we found without a HH random variable (Table G, conditional *R^2^* = 0.35 vs. Table 7, main text, adjusted *R^2^* = 0.26). A large-sample likelihood-ratio test of the random effect of HH number also yielded a significant *p*-value under the $\chi_{1}^{2}$-$\chi_{0}^{2}$ mixture distribution (Λ=23.602, *df* = 1, *p* = 5.92449 $\times{10}^{-7}$). However, a fixed effect for HH was again determined to be ineffective (not shown), and, given that it was non-independent from long-term HH characteristics in the model, we chose to omit the fixed effect from subsequent models.

**Table G.** GLMER of total daily amount received (inflow) by HH characteristics.


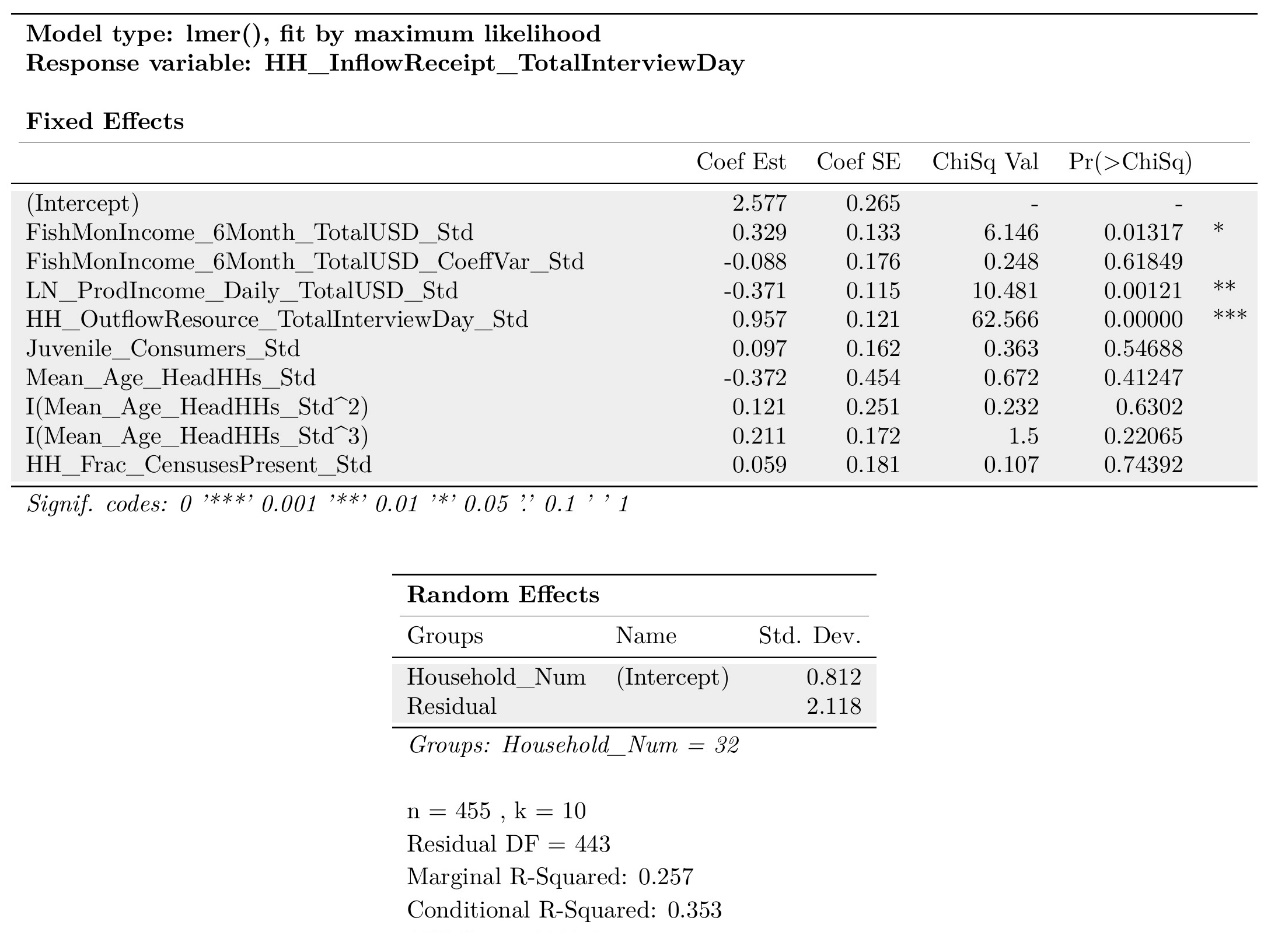


As above with the outflow models, we also divided inflow of resources into three sub-categories (food, money, other goods) in order to determine if they were transferred differently. The model of daily *food receipt* by Linao HH shows nearly the same set of significant predictor variables as the model including all resources (Table 9 vs. Table 7, main text). The model of mean daily food receipt over the entire sample period (Table H) accounts for considerably more variance (adjusted *R^2^* = 0.62) than the daily inflow model (Table 9, main text), but with only mean daily HH outflow of food as a significant predictor of mean daily food inflow.

**Table H.** GLM of HH mean daily inflow of **food** by HH characteristics.


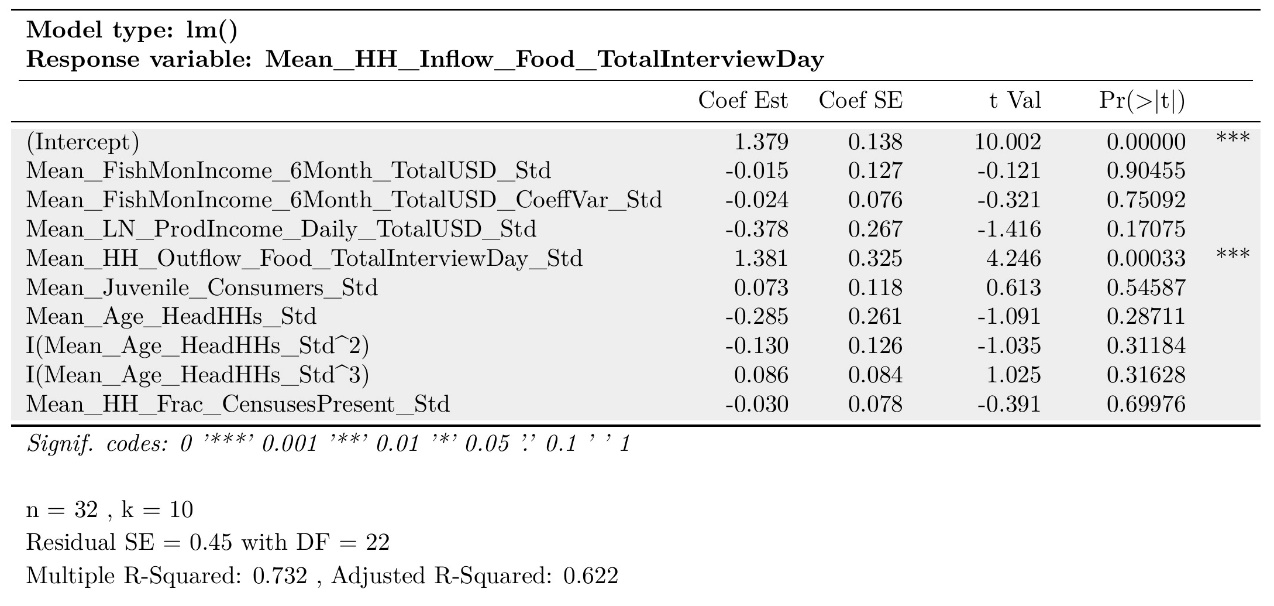


In contrast to the food model, when we examine daily inflow of *money*, we find that daily income from all sources is negatively associated with daily receipt of cash (Table 10, main text). The long-term model of mean daily receipt of money during the entire sample period shows that only mean daily HH outflow of money is statistically associated to mean receipt of money (Table I).

**Table I.** GLM of HH mean daily inflow of **money** by HH characteristics.


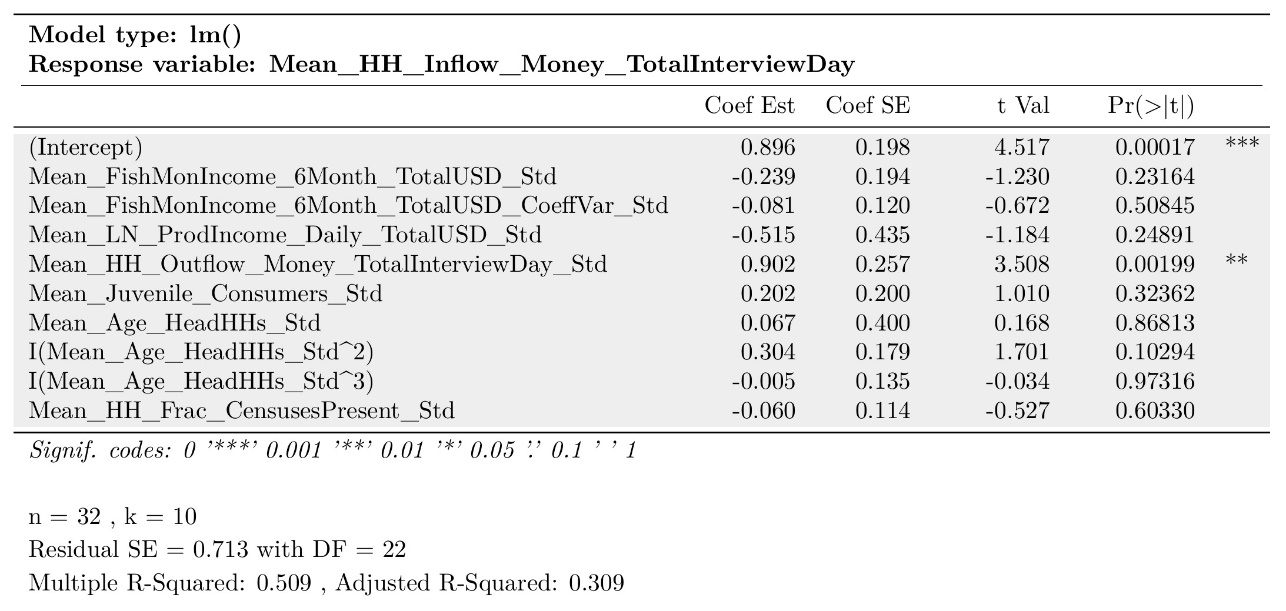


Finally, when we examine inflow of *other goods*, several variables are associated with receiving more (Table 11, main text). In the long-term model, number of juvenile consumers is statistically associated with receipt of other goods over the sample period, and both mean daily giving and age are also marginally statistically associated with receipt of other goods (Table J). However, surprisingly, mean number of juvenile consumers is *negatively* associated with the value of other goods received over the sample period.

**Table J.** GLM of HH mean daily inflow of **other** goods by HH characteristics.


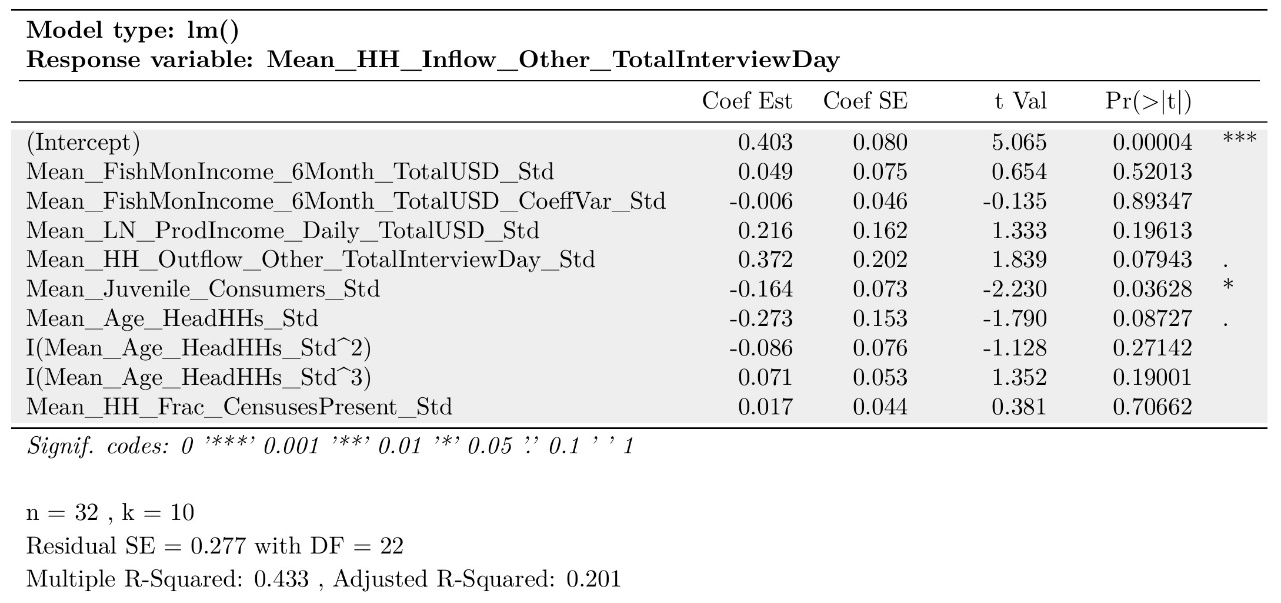


**Models of net outflow (giving)**

The 32 HH that we monitored throughout the study period showed considerable variation in NET outflow, ranging from a mean of about $+1 per day, to about $-5 per day (Figure N). Almost all households reported less outflow than inflow daily over the sample period. We are not sure why this is true, but there seems to be a reluctance to “brag” about how much HH members gave away on each day. In any case, some households have much higher net outflow than others, and some HH received a good deal of help (high net inflow). In general, we might expect that higher income houses show greater net outflow, and lower income houses greater net inflow, if helping the needy is an important part of the resource transfer pattern.


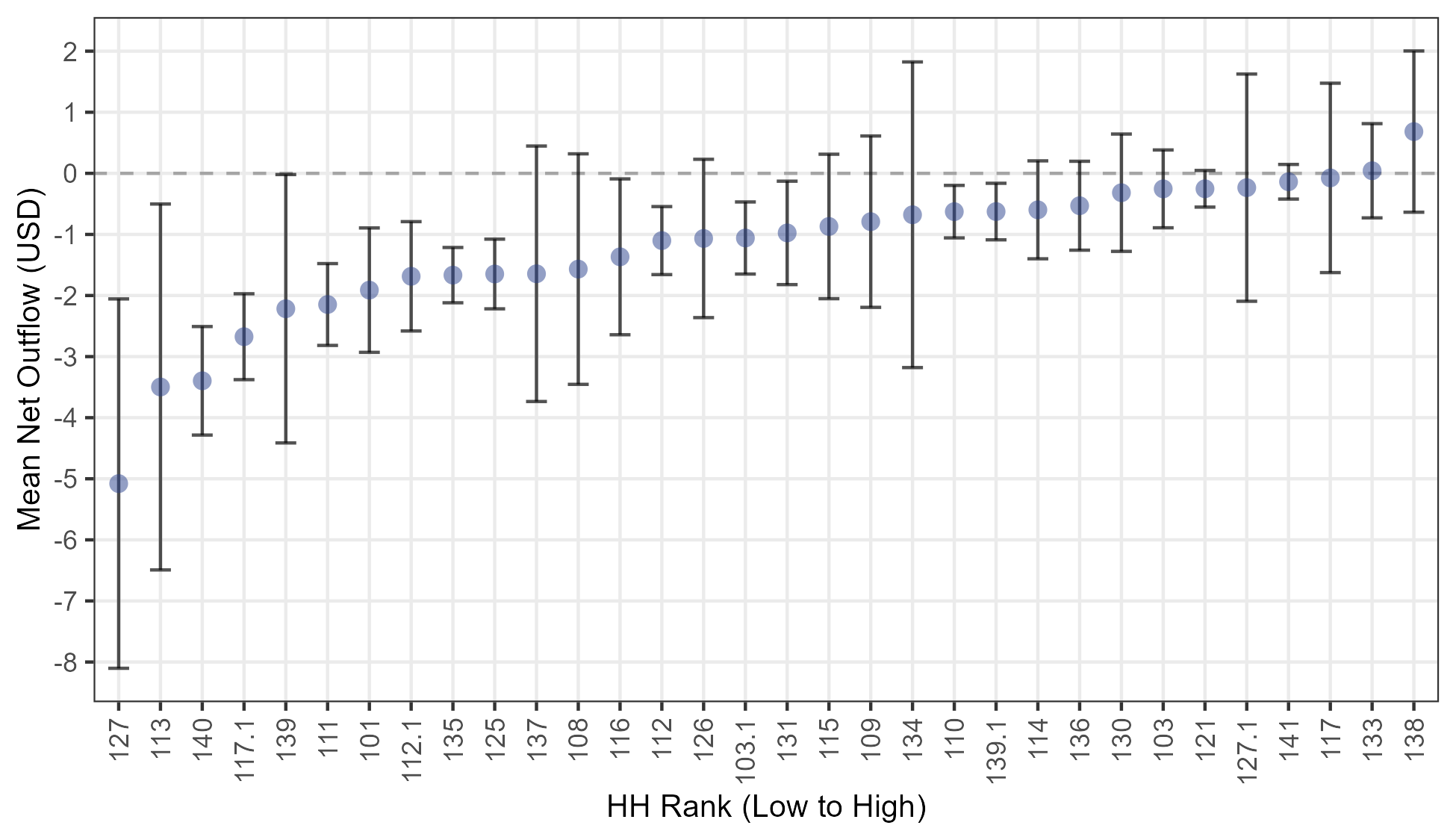


**Figure N.** *Mean daily net outflow by HH ranked from lowest to highest. Net outflow is measured as total daily outflow – total daily inflow on each interview day.*

Before developing statistical models, we examined the shape of net outflow by age and income, so that non-linear effects could be modeled appropriately. Based on LOESS smooths (Figure O), we constructed our models (below) with a third-order polynomial term for age, and the natural log of daily income.


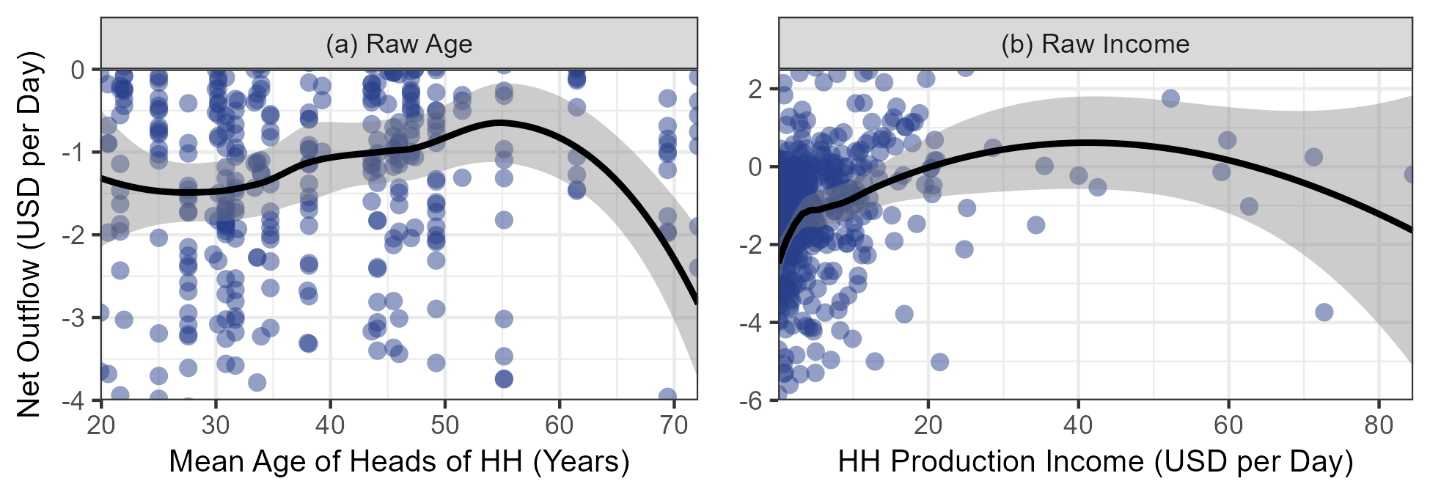


**Figure O.** *LOESS smooth (and 95% CI) of (a) age and (b) income by daily HH NET outflow for all interviews in the modeling sample (32 HHs). The y-axis of each plot has been cropped to enlarge the shape of the curve, but all data is included in LOESS smooth calculations. Range of the underlying daily net outflow data is between -$17.09 and $10.40 (high variability).*

**What determines the amount of daily net transfer to or from each household?**

The inflow and outflow model results seem most consistent with a reciprocity view of resource transfers where some HHs both give and receive more than other HHs (and generally tend to be higher income). Despite this fact, we can still examine whether some helping of needy HHs takes place within this pattern. This can be accomplished by examining what types of HHs were greater *net providers* (gave relatively more than they got) or greater *net recipients* (got relatively more than they gave) over the whole sample period, or on a day-by-day basis. Net outflow is defined as total value of goods given minus total value of goods received on the interview day, or over the whole sample period. Net inflow is the opposite of net outflow. Since the two GLM models are mirror images, they give identical statistical results, but with opposite coefficient signs (daily net outflow = -1 × daily net inflow), so we present only the net outflow model here.

The GLM models of net outflow examine the same HH characteristics as the GLM models for giving and receiving, and they include the same non-linear variables (Figure O). Results of the daily net outflow model (Table K) suggest that higher income on the day of the interview is associated with greater net outflow on that interview day, and that older HHs show low net outflow compared to middle-aged HHs who show the highest levels of daily net outflow (Figure P). Importantly, there is no evidence that HHs with higher mean long-term (6 month) fishing income exhibit higher net outflow on single interview days, or that families with lower long-term income are generally net recipients. However, the explanatory power of this model is very poor (Table K, adjusted *R^2^* = 0.07), possibly because both high-outflow-high-inflow HHs and low-outflow-low-inflow HHs show net outflow around the same low values. When reciprocity is the main cause of transfers, both high- and low-income HHs may experience nearly the same net outflow levels, where giving is balanced by receiving.

**Table K.** GLM of daily net outflow.


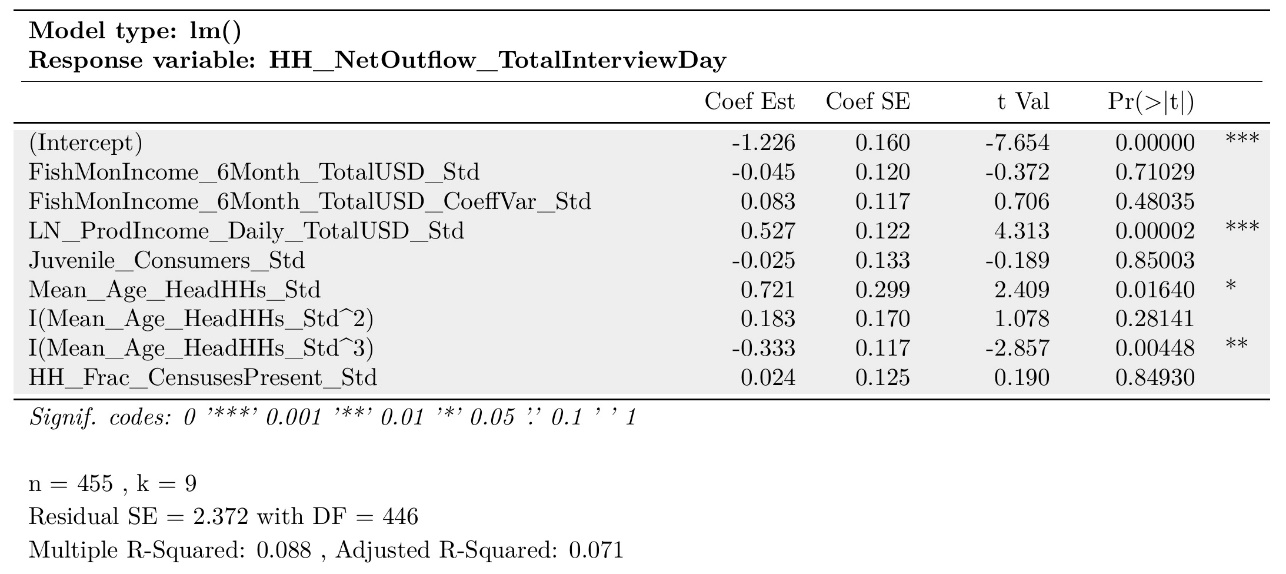


The age effect from the GLM daily net outflows model confirms a statistically significant decrease in net outflows by the most elderly HHs (Figure P), just as the LOESS smooth suggested. These older HHs, showing greatest net receipt (lowest net outflow), generally have younger adult (married) children and grandchildren, as well as elderly heads of HH.


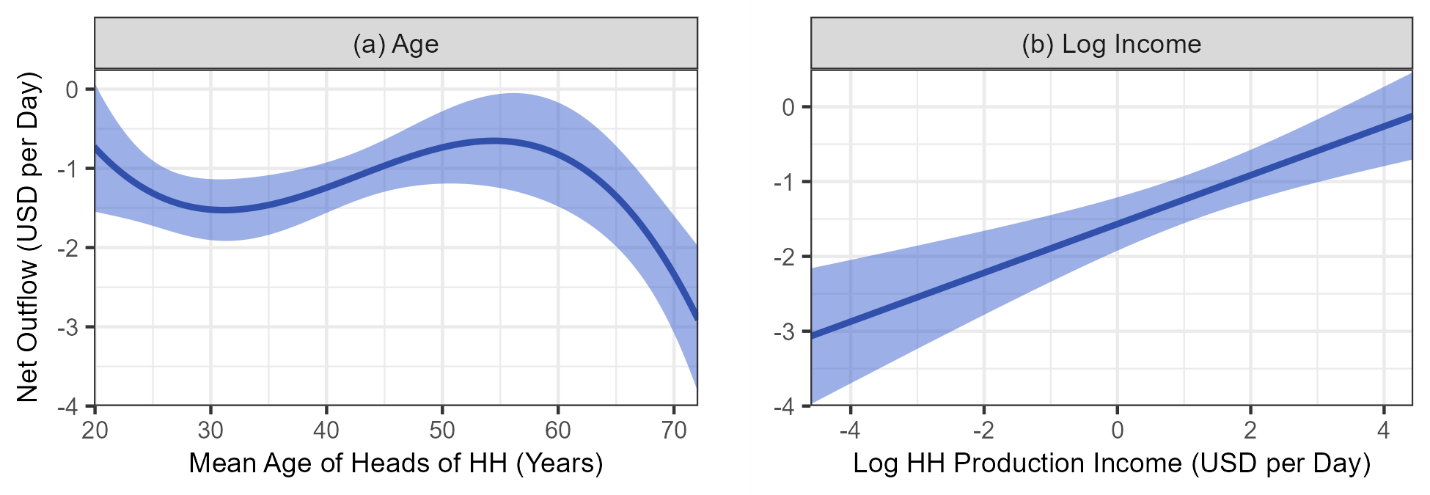


**Figure P.** *The shape (95% CI) of (a) non-linear effect of mean age of HH heads and (b) linear effect of natural log of production income on daily net HH outflow of all goods. Independent variables were converted back from standardized to raw variable values, and all other variables were set to the sample mean value.*

Next, we examined mean daily net giving for each of the 32 HHs over the entire sample period. Predictor variables were *mean* fishing income and production income over the entire sample period (and coefficient of variation of fishing income), *mean* number of juvenile consumers, *mean* age of HH heads, and permanence of residence. The overall fit of this GLM model is still very poor (Table L, adjusted *R^2^* = 0.02), and none of the predictor variables are significantly associated with level of net outflow (or net inflow) over the entire sample period (Table L). However, a plot of mean net outflow by HH, divided into income categories that consider both absolute and per capita income (defined in Figure E) does suggest that “rich” HHs are generally characterized by higher mean daily net outflow, but with a large amount of scatter between HHs within each income category, ranging from “poor” to “rich” (Figure Q).

**Table L.** GLM of mean net daily amount given (outflow) by HH characteristics.


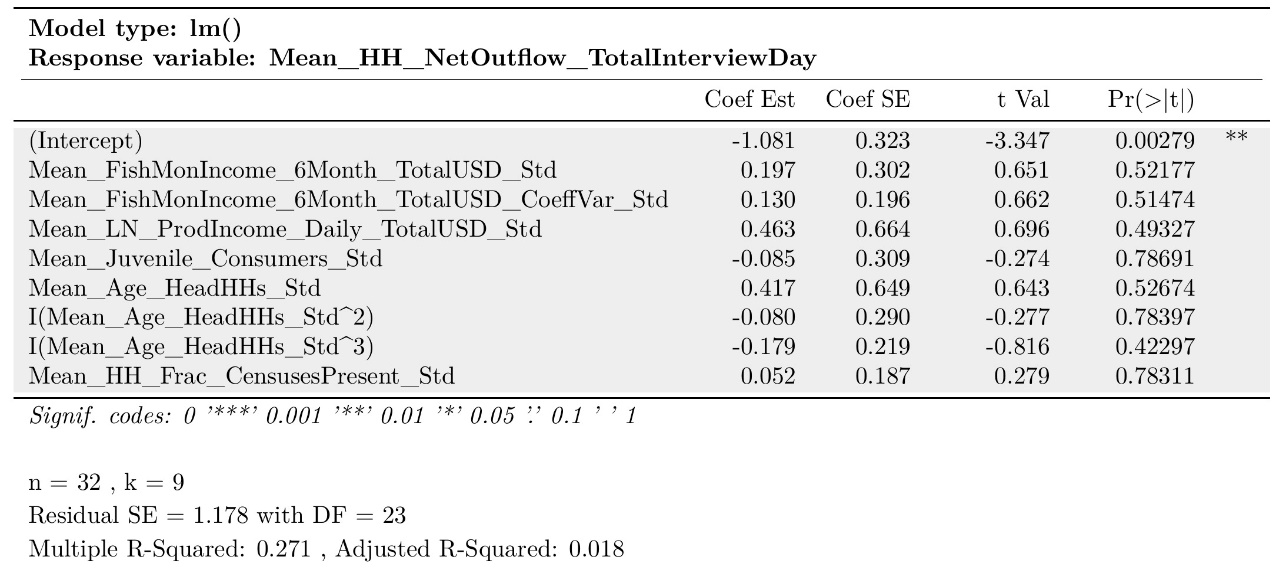


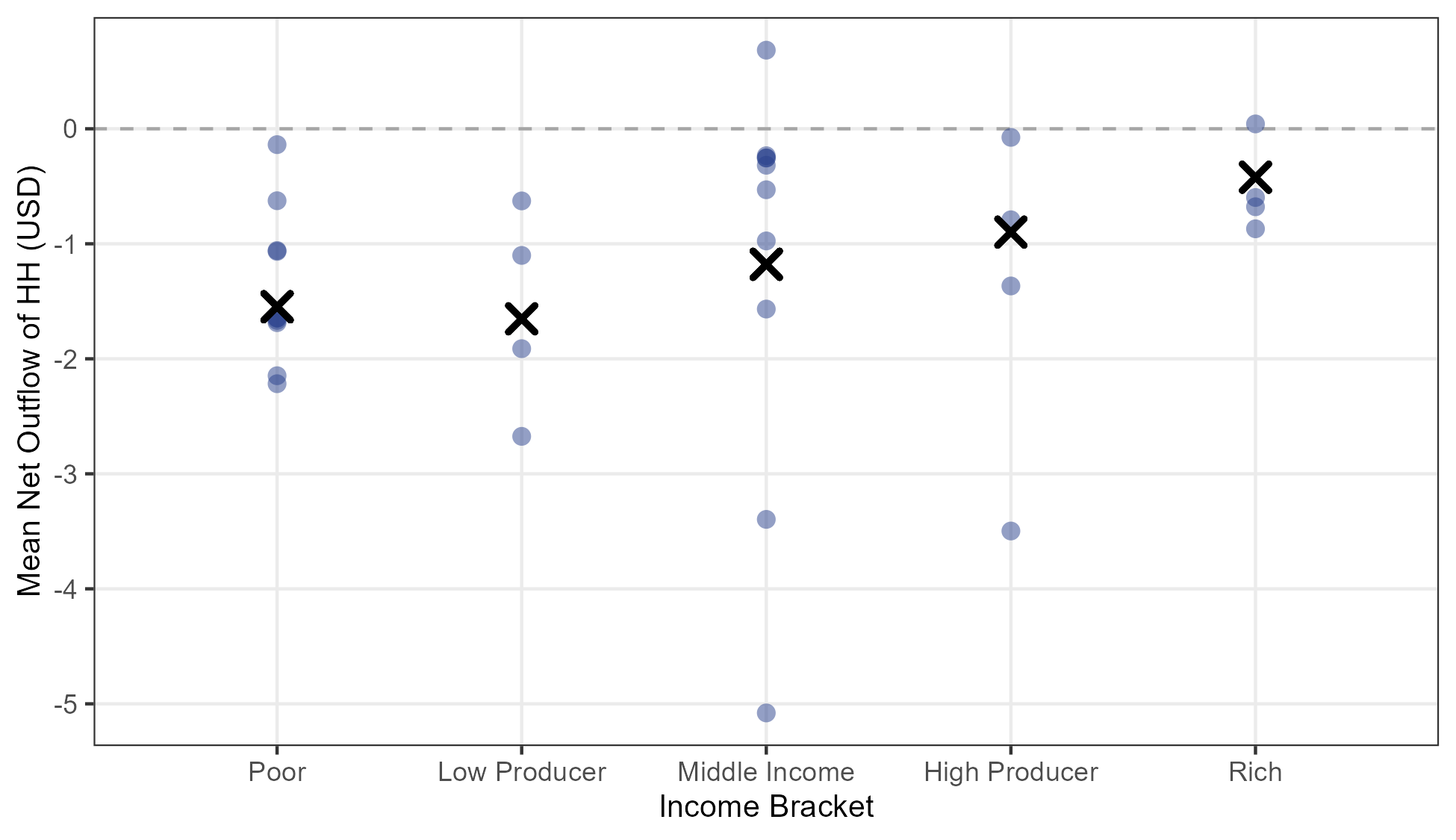


**Figure Q.** *Mean daily net outflow for 32 HHs which were sampled at least 3 days. Small blue circles show the mean for each HH, and large black X indicates the mean for the income category. Income categories include both per capita and absolute measures of production and are defined in Figure E. HHs with high per capita income (rich) show the highest net outflow, and HHs with low per capita income (poor) show lowest mean net outflow.*

**Beliefs about patterns of giving and receiving**

We interviewed 12 men and 24 women concerning what types of HH they believed gave more or received more material goods during our study period. Questions were phrased to elicit a yes/no answer, and we calculated the proportion of *yes* answers for each trait. Results are shown in Table M, where we present the proportion of *yes* answers for each question. For each question, if the sample 95% confidence interval for a *yes* answer was >50%, we considered the community answer to be YES, and if the 95% confidence interval was <50%, we considered the community answer to be NO.Questions for which the statistical estimate of proportion affirmative included the 50^th^ percentile were considered to provide inconclusive results.

**Table M**. Linao opinions about types of HH that give or receive more


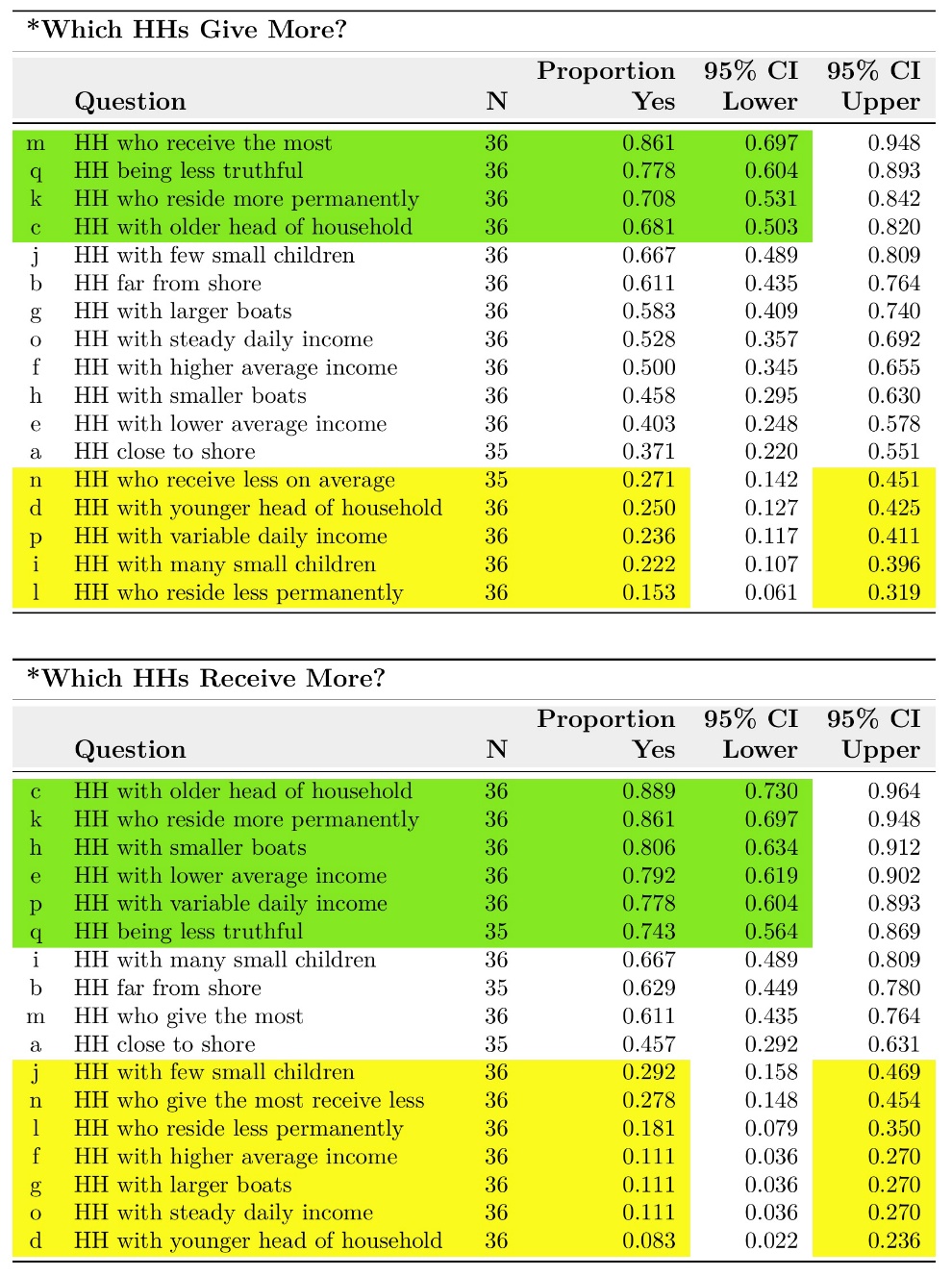


**Summary of model results**

Table N summarizes the results of all models examining which types of HH give most, receive most, and which types of dyads give and receive more from each other.

**Table N**. Results from all models
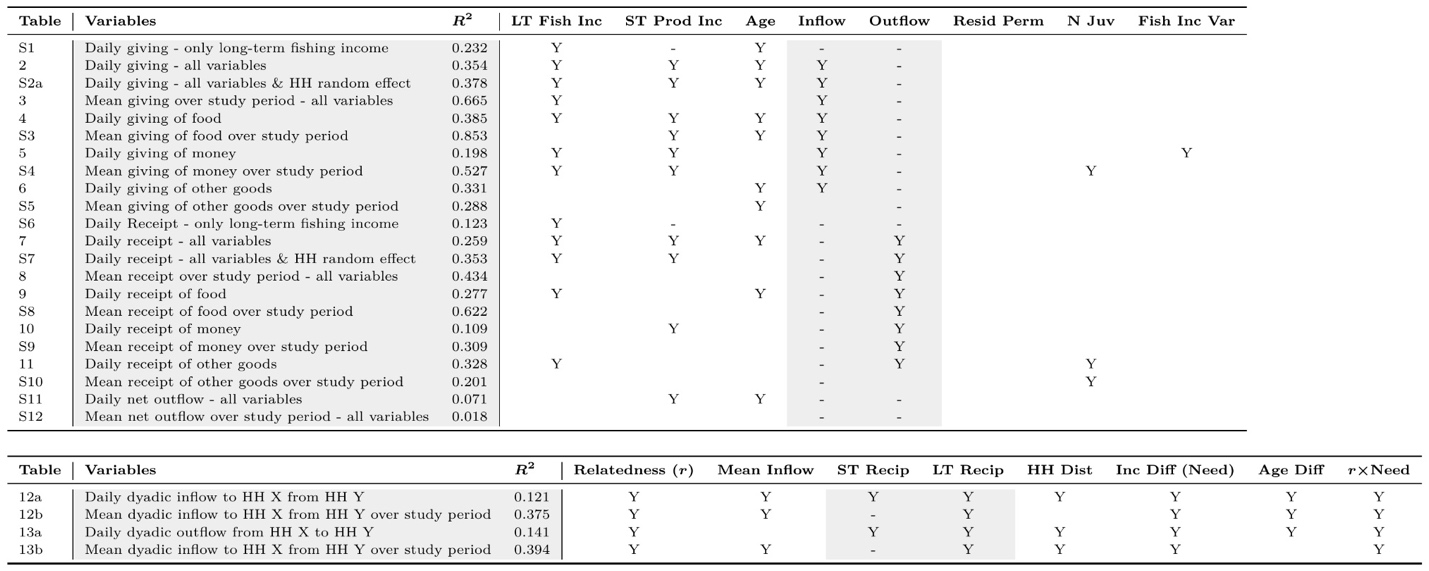


*Results of all models in main text and SOM. ‘Y’ denotes significance of the variable at α = 0.05 or smaller; else, variable is non-significant in the model. Variables not included in a particular model are indicated by ‘-‘. Abbreviations used: LT Fish Inc (Long-Term Fishing Income); ST Prod Inc (Short-Term Production Income); Resid Perm (Residential Permanence); N Juv (Number of Juveniles); Fish Inc Var (Fishing Income Variance); ST Recip (Short-Term Reciprocity); LT Recip (Long-Term Reciprocity); HH Dist (Household Distance); Inc Diff (Income Difference, i.e. relative need); Age Diff (Age Difference between heads of households); r x Need (Relatedness x Need, i.e. kin selection)*

**Kinship and reciprocity**

There is some interest in the question of whether dyadic reciprocity partners are often or mainly kin relations. While standard behavioral ecology once partitioned transfers and exchanges into those that were motivated by inclusive fitness gains (resource transfers to kin) and those that were examples of reciprocal altruism (non-kin transfers), empirical studies have sometimes shown that the coefficient of contingency (giving predicted by receipt, and vice versa) for reciprocal dyadic exchange is as strong between kin as it is between non-kin (e.g. [4]). Here, we reanalyze our data on daily dyadic resource flows (inflows to HH X from HH Y; outflows from HH X to HH Y), dividing all analyses sets into close kin (coefficient of relatedness *r* >= 0.125), distant kin (0.125 > *r* >= 0) and non-kin (*r* = 0). The HH inflow GLM models suggest that the reciprocity coefficients for long-term mean inflow from different HHs are about the same in the close kin, distant kin, and non- kin sample of HHs (Tables O-Q). Analyses using daily outflow to HH Y as a predictor of reciprocity, rather than long-term mean outflow to HH Y, gives the same result (not shown).

**Table O.** Daily inflow from HH Y (CLOSE KIN ONLY)


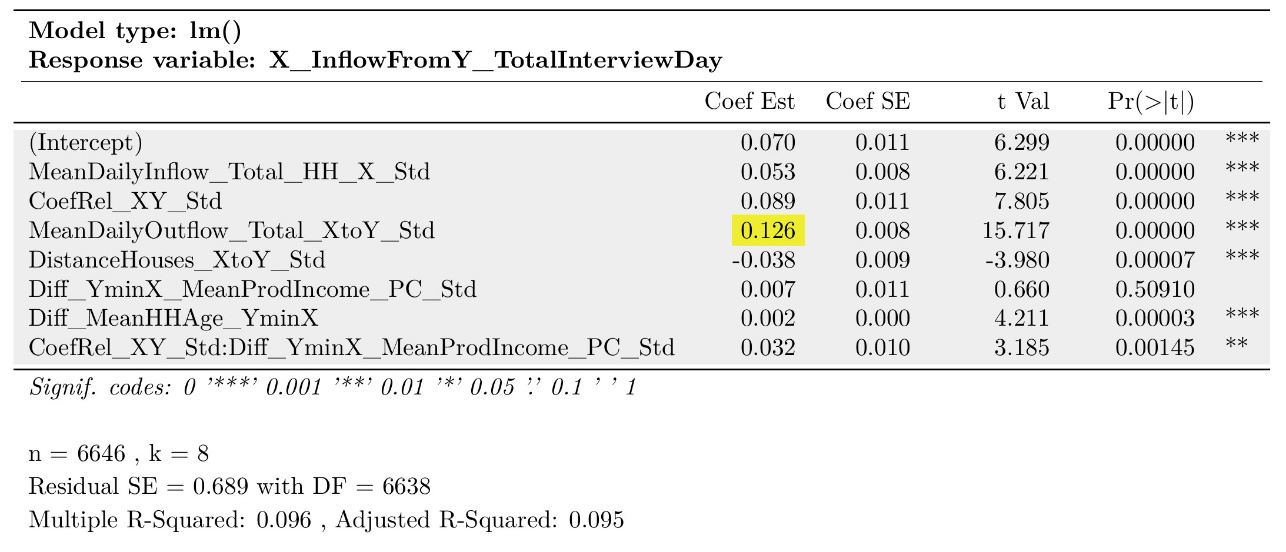


**Table P.** Daily inflow from HH Y (DISTANT KIN ONLY)


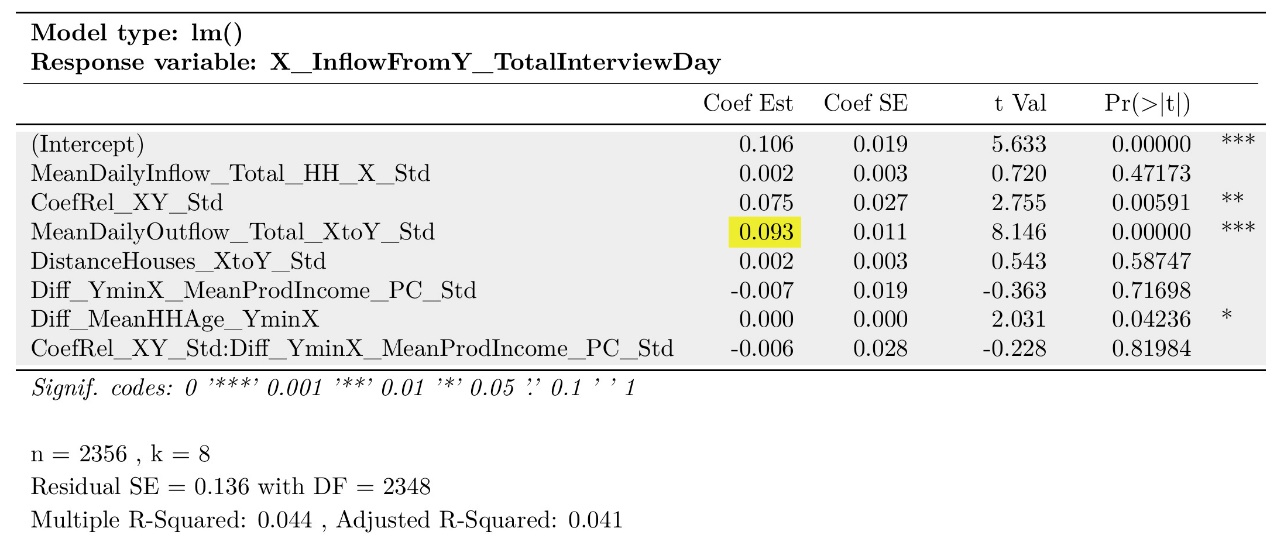


**Table Q.** Daily inflow from HH Y (NON- KIN ONLY). The effect of relatedness and the interaction term between relatedness and mean per capita income difference are omitted, since relatedness is fixed at 0.


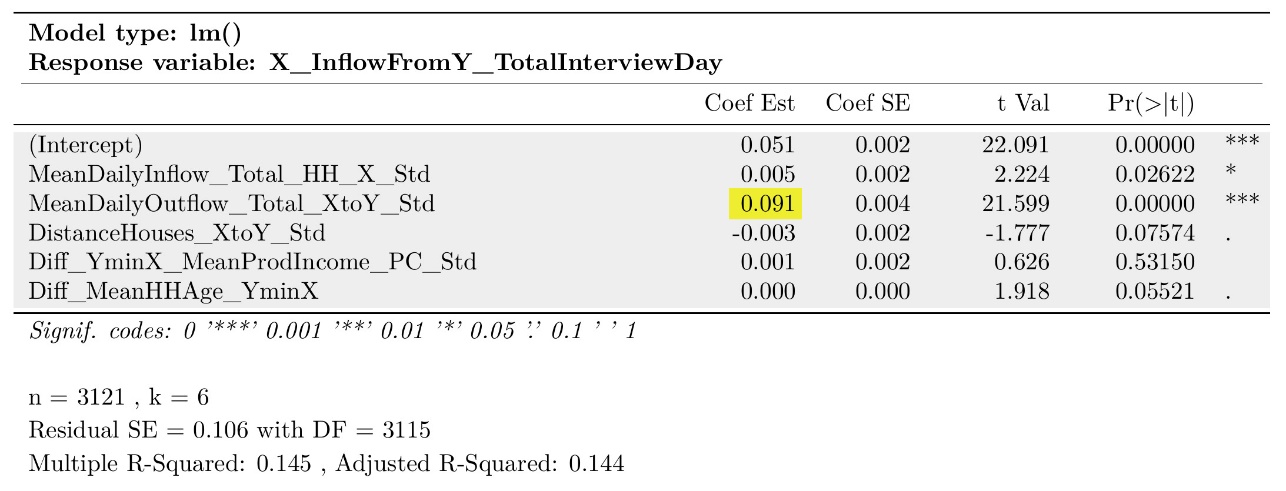


On the other hand, the GLM models of daily outflow to HH Y suggest that the reciprocity coefficients are slightly higher for the non-kin sample of HHs than for the close kin and distant kin subsets of the village (Tables R-T). Analyses using daily inflow from HH Y, rather than long term mean inflow from HH Y, give the same result (not shown).

**Table R.** Daily outflow to HH Y (CLOSE KIN ONLY)


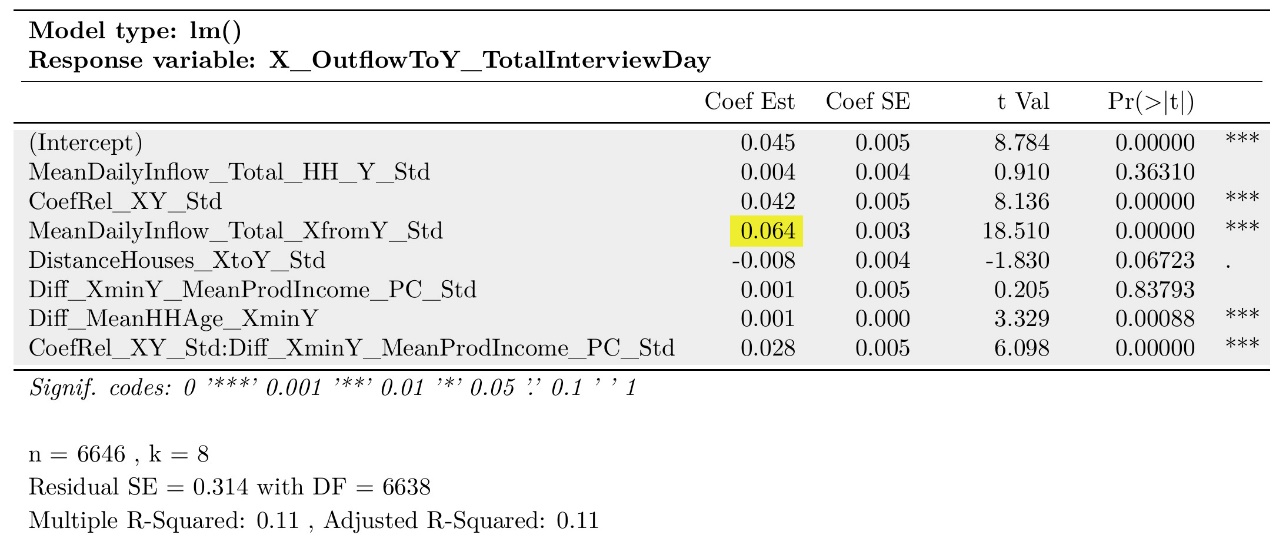


**Table S.** Daily outflow to HH Y (DISTANT KIN ONLY)


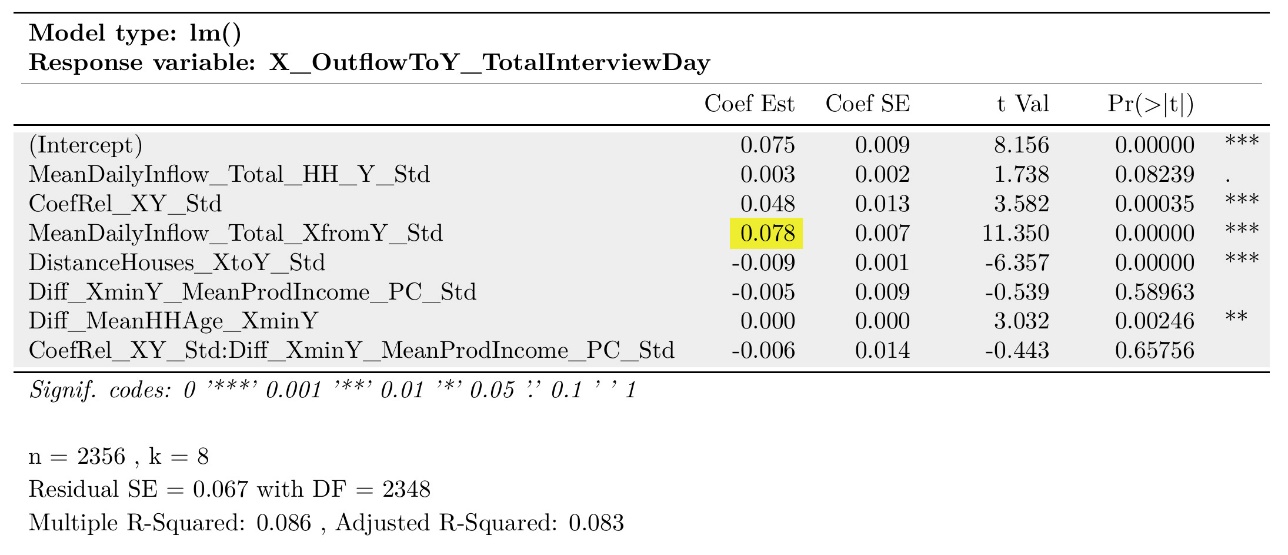


**Table T.** Daily outflow to HH Y (NON- KIN ONLY). The effect of relatedness and the interaction term between relatedness and mean per capita income difference are omitted, since relatedness is fixed at 0.


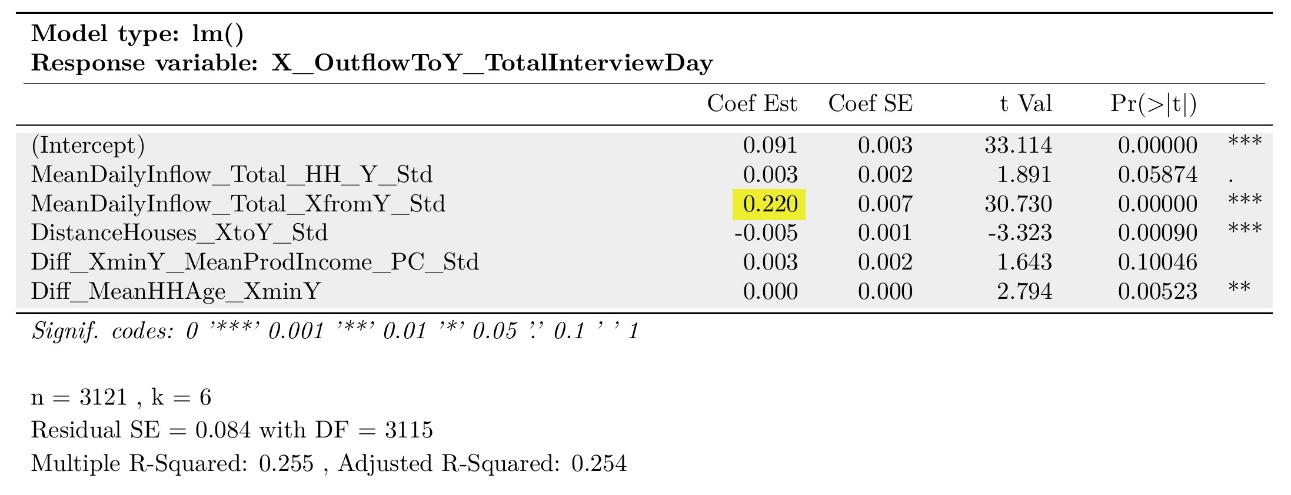


The fact that inflow patterns show no difference in the importance of reciprocity for resources received by kin or non-kin, but that outflow patterns do, suggest that reciprocity may be somewhat more important among non-kin, but we cannot yet draw strong conclusions about whether reciprocity is as important between kin as it is between non-kin as a motivator of resource transfers. Nevertheless, as we show below, high levels of symmetrical transfer AND asymmetrical transfer of goods between households were highest between close-kin.

**Discussion**

Giving by distantly-related kin was impressive and varied. Informant statements implied that any recognizable relationship between two individuals can provide justification for transfers of resources. Approximately 10% of the resource receipt events listed in interviews (956 out of 9315 total events) were reported to have come from *distant kin*, defined as individuals with a genetic coefficient of relatedness *r* greater than 0 but less than 0.125 (first cousins). This includes donors of goods in 67 different specified relationships to the recipient (Table U) and separated by up to 7 specified kinship links (e.g. “fa mo mo sis so so so”).

**Table U.** All events of received goods (inflow) by distant kin according to kinship links reported.


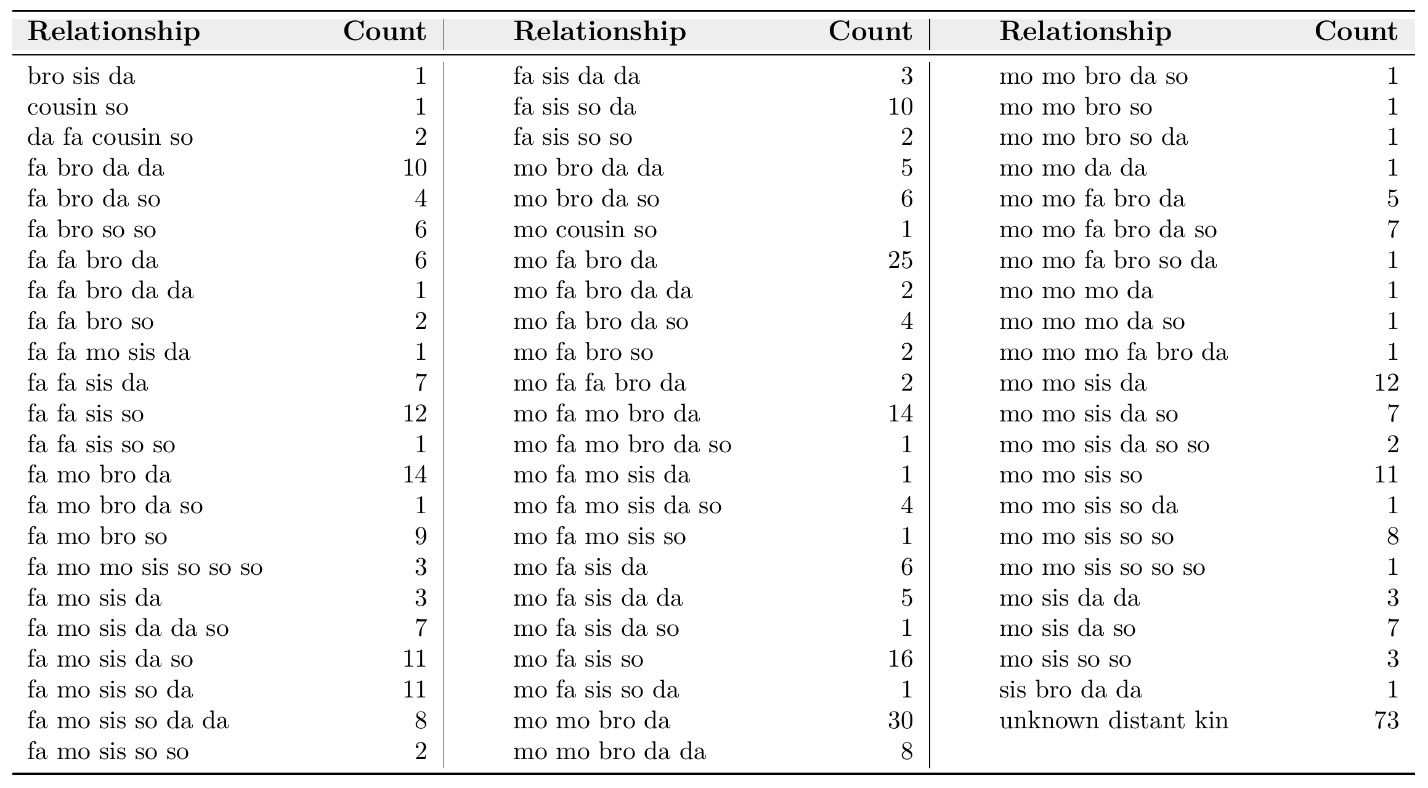


Likewise, giving by distant affines was commonly reported and even more highly varied. Aside from the closely-related affines (father and mother in-law, brother and sister in-law, son and daughter in-law), there were 146 reported categories of kin related by a combination of marriage links and consanguineal links who were listed as donors of material goods received by individuals in Linao (Table V). This included individuals who were related by up to 7 specified links of marriage and genes (e.g. “fa mo sis da sp bro so”).

**Table V.** All events of received goods (inflow) by distant affines according to links reported by interviewees.


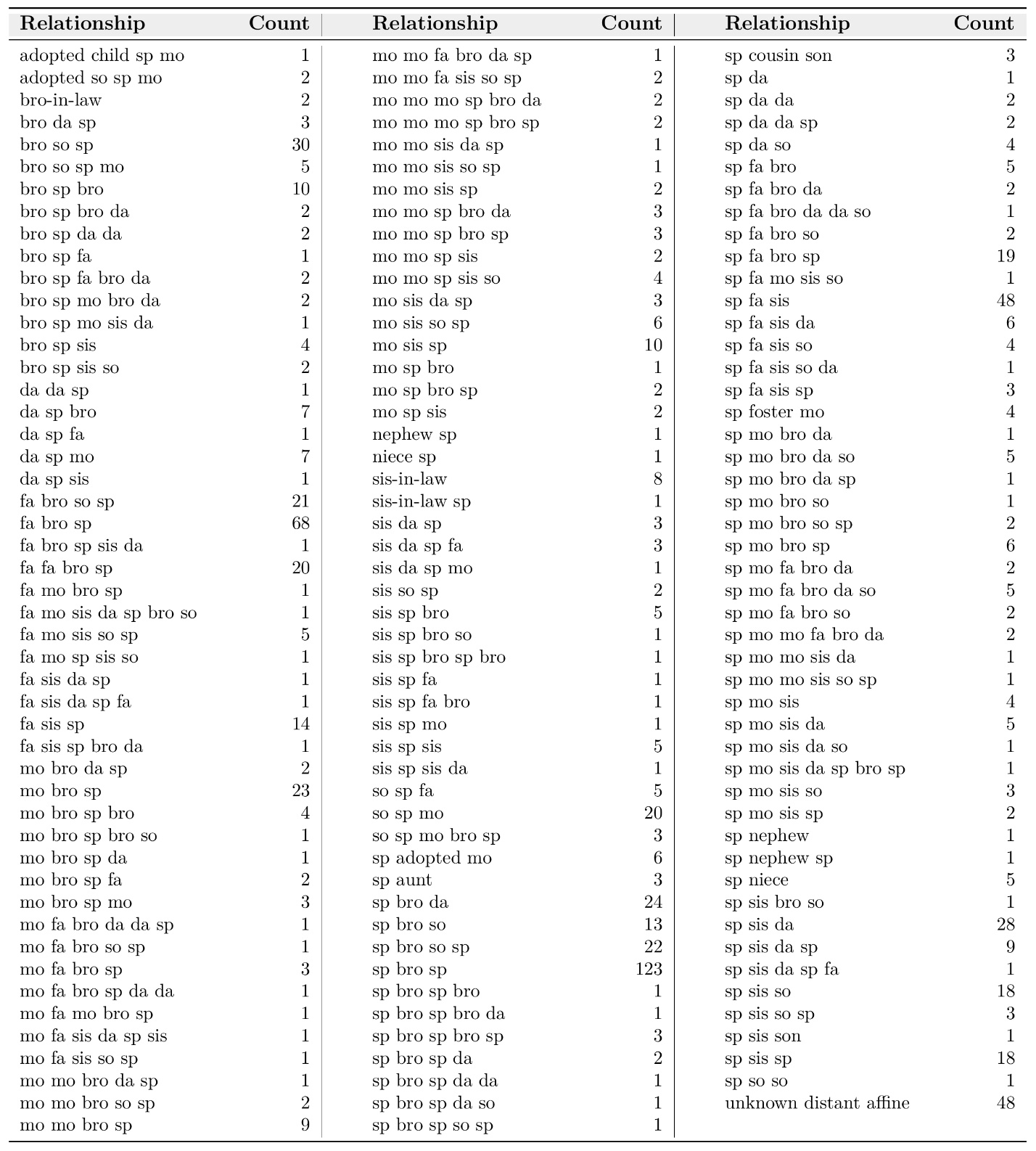


**Time scale of reciprocity**

There is some concern over whether we were actually observing an informal reciprocity system in our study village, or whether we simply detected flows due to more formalized daily trade or even commercial exchanges. We consider the main difference between *reciprocity* and *trade* to be one of time scale of repayment, and whether exchange prices are formalized and based clearly on agreed-upon repayment. For this reason, we explained to interview subjects that we wished them to list only resource transfers that had no agreed-upon dates of repayment, nor prices (exchange value of goods given and received). Nevertheless, because reciprocity coefficients between dyads are fairly high (Tables 12a, 12b, 13a, 13b in main text), we decided to examine the daily value of goods given and received by specific HH dyads. If transfers are indeed *commercial exchange* or *trade*, we expect the value of goods given and goods received to be nearly equivalent for most HH dyads on most days. Instead, what we found was a great deal of daily disparity, with many single-day transfers including a huge asymmetry in value of what was given by one HH on that day and shared in turn by the other HH on that same day (Figure R). However, the dyadic balance in value given and received is considerably more symmetrical when we examine the entire study period (Figure H, S), just as we might expect from an informal reciprocity system of cooperative resource transfers.


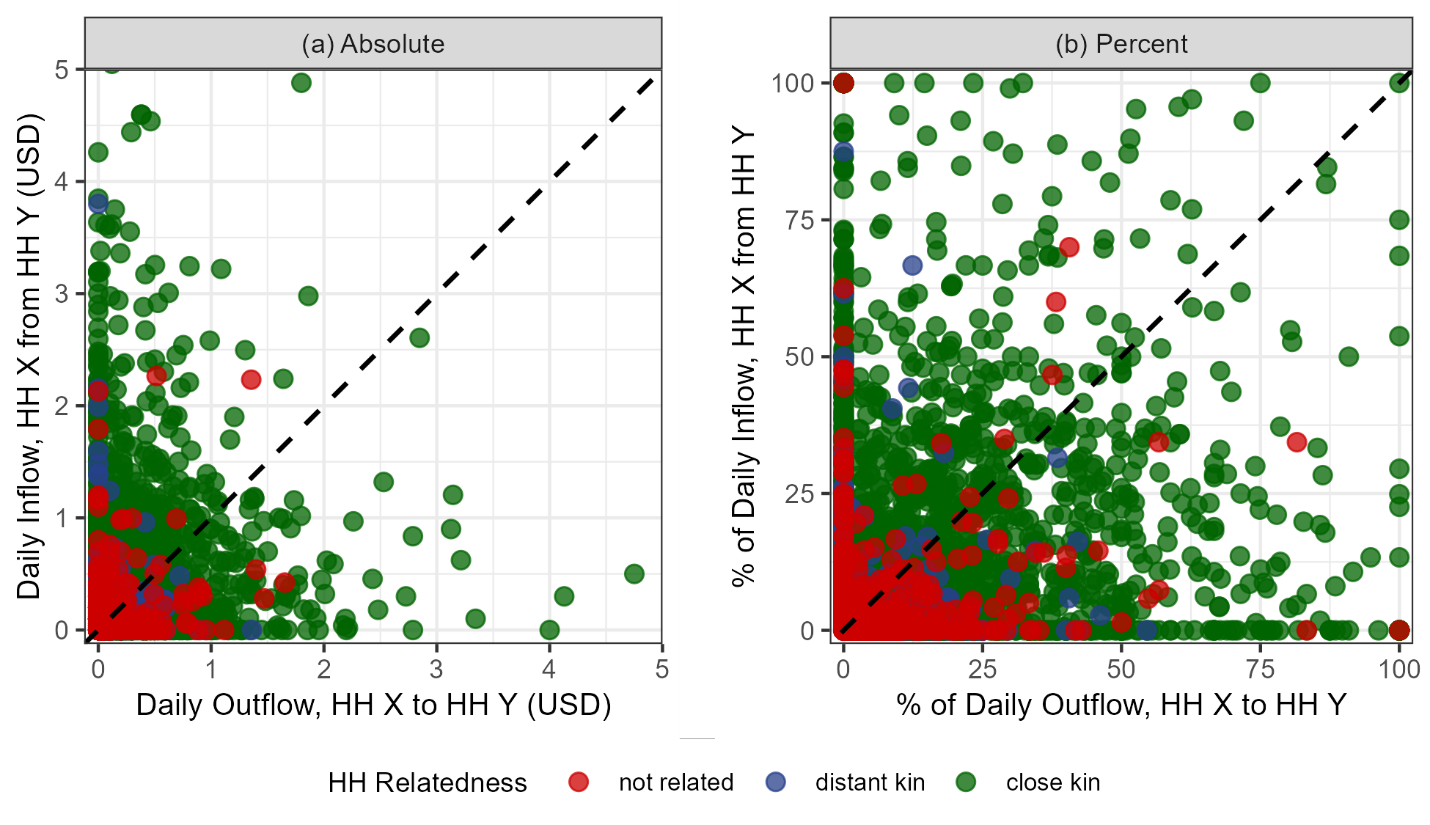


**Figure R.** *The daily balance in (a) absolute HH value and (b) percentage of HH total value of goods given and received for each pair of HH in our study sample. Fourteen outlier points have been cropped out of (a) to focus on main pattern of data; all are close kin. Note the large number of data points where one HH either gave or received nothing on a day when the other HH gave or received a considerable value of goods.*


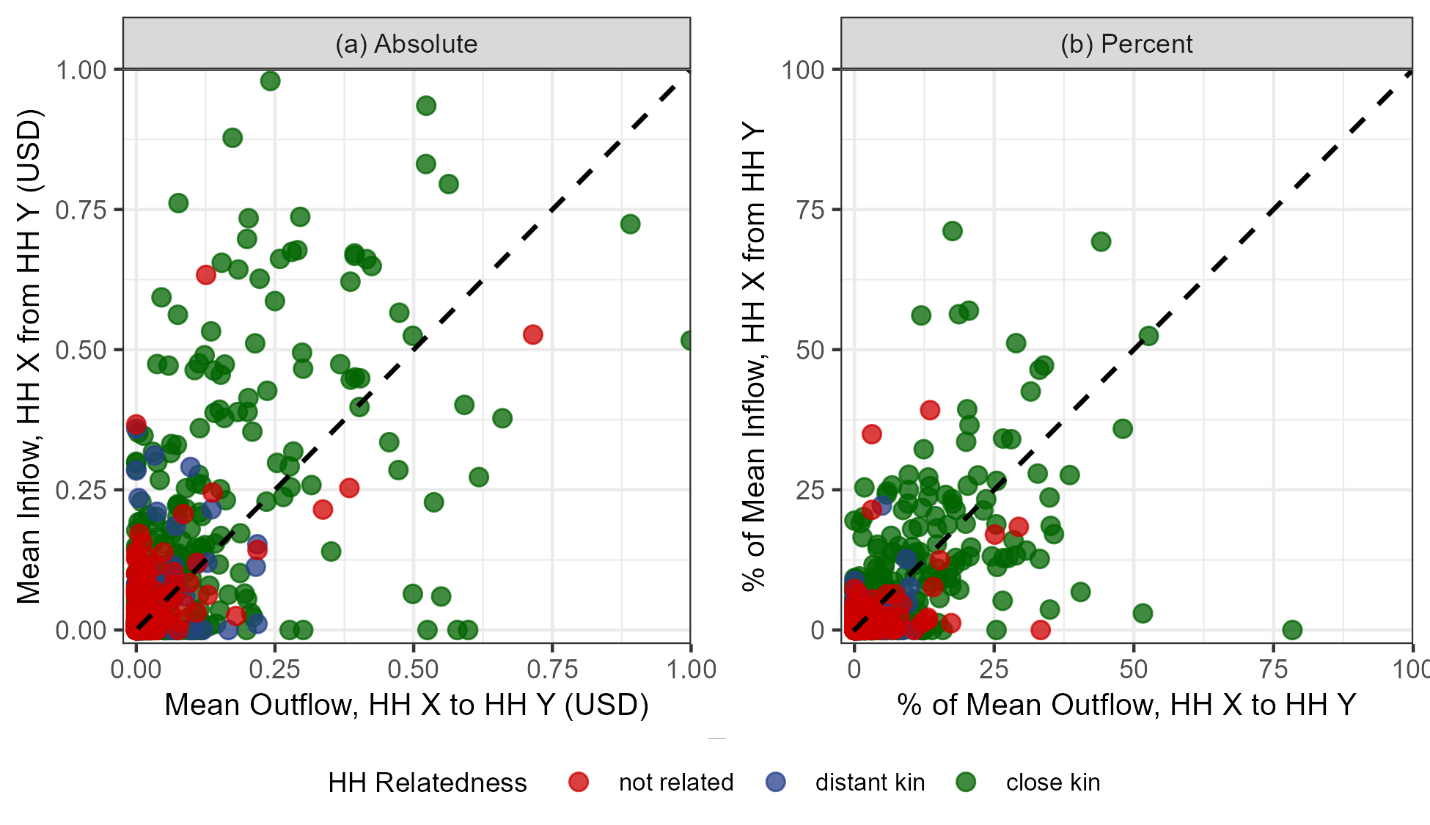


**Figure S.** *The mean long-term balance in (a) absolute HH value and (b) percentage of HH total value of goods given and received for each pair of HH in our study sample. Sixteen outlier points have been cropped out of (a) to focus on main pattern of data; all are close kin. Note the data points where one HH either gave or received nothing whereas the other HH gave or received a considerable value of goods. Note also that high levels of reciprocal transfer are generally limited to close kin HH.*

**References**

1. Sather C. The Bajau Laut: adaptation, history, and fate in a maritime fishing society of south-eastern Sabah. Kuala Lumpur ; New York: Oxford University Press; 1997. 359 p. (South-East Asian social science monographs).

2. Sather C. Sea Nomads and Rainforest Hunter-Gatherers: Foraging Adaptations in the Indo-Malaysian Archipelago. In: Bellwood P, Fox JJ, Tryon D, editors. The Austronesians: Historical and Comparative Perspectives [Internet]. 1st ed. ANU Press; 2006 [cited 2022 Sep 11]. Available from: http://press-files.anu.edu.au/downloads/press/p69411/pdf/ch1315.pdf

3. Philippines GDP per capita - 2022 Data - 2023 Forecast - 1960-2021 Historical - Chart [Internet]. Trading Economics.com. 2019. Available from: https://tradingeconomics.com/philippines/gdp-per-capita

4. Allen-Arave W, Gurven M, Hill K. Reciprocal altruism, rather than kin selection, maintains nepotistic food transfers on an Ache reservation. Evol Hum Behav. 2008 Sep;29(5):305–18.
